# Electrocatalytic Nitrate and Nitrite Reduction toward Ammonia Using Cu_2_O Nanocubes: Active Species and Reaction Mechanisms

**DOI:** 10.1021/jacs.3c13288

**Published:** 2024-04-01

**Authors:** Lichen Bai, Federico Franco, Janis Timoshenko, Clara Rettenmaier, Fabian Scholten, Hyo Sang Jeon, Aram Yoon, Martina Rüscher, Antonia Herzog, Felix T. Haase, Stefanie Kühl, See Wee Chee, Arno Bergmann, Roldan Cuenya Beatriz

**Affiliations:** Department of Interface Science, Fritz-Haber-Institute of Max-Planck-Society, Faradayweg 4-6, 14195 Berlin, Germany

## Abstract

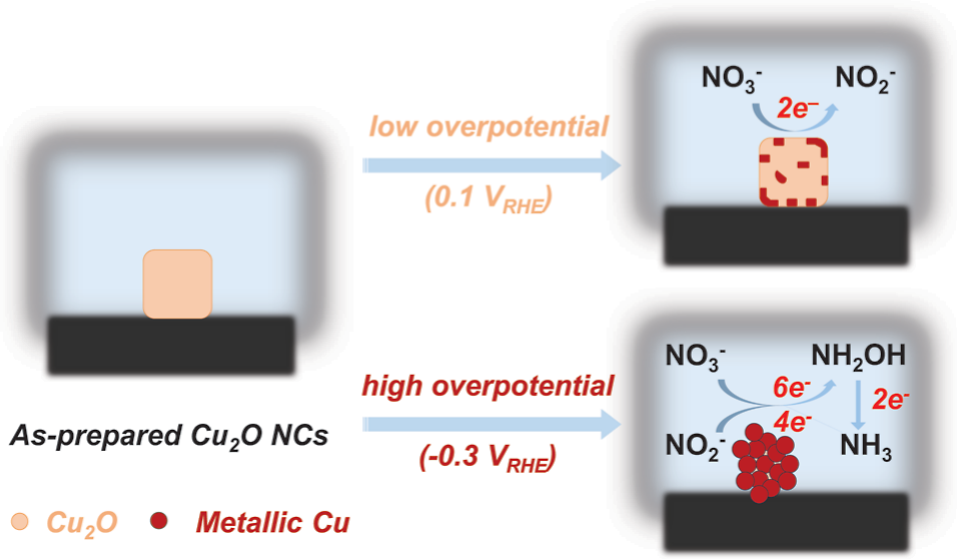

The electrochemical reduction of nitrate (NO_3_^–^) and nitrite (NO_2_^–^) enables sustainable, carbon-neutral, and decentralized routes to produce ammonia (NH_3_). Copper-based materials are promising electrocatalysts for NO_*x*_^–^ conversion to NH_3_. However, the underlying reaction mechanisms and the role of different Cu species during the catalytic process are still poorly understood. Herein, by combining quasi in situ X-ray photoelectron spectroscopy (XPS) and operando X-ray absorption spectroscopy (XAS), we unveiled that Cu is mostly in metallic form during the highly selective reduction of NO_3_^–^/NO_2_^–^ to NH_3_. On the contrary, Cu(I) species are predominant in a potential region where the two-electron reduction of NO_3_^–^ to NO_2_^–^ is the major reaction. Electrokinetic analysis and in situ Raman spectroscopy was also used to propose possible steps and intermediates leading to NO_2_^–^ and NH_3_, respectively. This work establishes a correlation between the catalytic performance and the dynamic changes of the chemical state of Cu, and provides crucial mechanistic insights into the pathways for NO_3_^–^/NO_2_^–^ electrocatalytic reduction.

## Introduction

Ammonia (NH_3_) is an essential feedstock for the industrial production of various chemicals, including fertilizers, synthetic fibers, and pharmaceuticals.^1−3^ It also serves as a potential carbon-free energy carrier with high energy density and excellent hydrogen storage capacity.^4,5^ Nowadays, large-scale NH_3_ production is supplied by the Haber–Bosch process, which is largely dependent on fossil fuels (used for the generation of H_2_ from CH_4_) and contributes significantly to the emissions of carbon dioxide.^1−3^ Moreover, the increasing rate of anthropogenic nitrogen fixation has led to a persistent imbalance in the nitrogen cycle,^6,7^ ultimately resulting in an accumulation of harmful nitrate (NO_3_^–^) and nitrite (NO_2_^–^) in groundwater, which could have serious negative impact on both the environment^3,8−10^ and the human health.^3,11,12^ The electrochemical reduction of nitrate (NO_3_RR) and nitrite (NO_2_RR), powered by renewable energy sources, has recently attracted growing interest as a sustainable and carbon-neutral route to produce NH_3_ directly from the industrial and agricultural wastewater.^3,6,13−15^ This approach would favor a decentralized NH_3_ production in local communities.^13−15^

Due to the extreme complexity of the overall NO_3_RR and NO_2_RR processes, which involve multiple proton/electron-transfer steps and intermediates, suitable catalysts are essential to control the selectivity toward a specific target product and to drive an efficient NH_3_ production for practical and technological applications.^3,15−17^ Copper-based materials are promising catalysts for electrochemical NO_3_RR and NO_2_RR due to the properties of low cost, high selectivity for NH_3_ production and relatively low overpotentials.^3,16,17^ Several strategies including alloying,^18−20^ doping,^21−23^ facet control^24−27^ and defect engineering^24,28−30^ were employed to further improve the catalytic performance of Cu catalysts. However, the underlying reaction mechanisms and the identity of active sites responsible for NH_3_ formation of Cu-based catalysts remain under debate.^21,31−33^ For instance, CuO nanowire arrays were reported to undergo an in situ electrochemical reconstruction to generate a mixed Cu/Cu_2_O active phase, which serves as a catalyst for NH_3_ production.^31^ Several highly active Cu-based bimetallic catalysts were reported to be in metallic state under NO_3_RR conditions.^18,21^ Elsewhere, highly active defective CuO nanostructures were converted to mixed CuO/Cu_2_O under catalytic NO_*x*_RR conditions, with no evidence reported for metallic Cu.^28^ Some recent studies have also considered cuprous(I) oxide (Cu_2_O) to be the real catalyst for ammonia formation, which is supposed to reduce the energy barrier for NH_3_ formation and suppress the competing hydrogen evolution reaction (HER).^32,33^

Here, we use Cu_2_O nanocubes (NCs) as model precatalysts for both NO_3_RR and NO_2_RR due their well-defined structures and initial composition, combined with the absence of surface-adsorbed ligands, and track the chemical state of Cu as a function of the applied potential to establish a correlation with the NH_3_/NO_2_^–^ selectivity. A combination of grazing incidence X-ray diffraction (GI-XRD), transmission electron microscopy (TEM), quasi in situ X-ray photoelectron spectroscopy (XPS), and operando X-ray absorption spectroscopy (XAS) measurements were used to follow the dynamic changes of Cu_2_O NCs occurring both in the bulk (beneath the surface) and on the surface under electrocatalytic NO_3_RR and NO_2_RR conditions. We reveal that Cu(I) species are predominant during the initial two-electron NO_3_^–^-to-NO_2_^–^ reduction step at low overpotentials, whereas metallic copper decisively contributes to multielectron stepwise reduction of NO_3_^–^/NO_2_^–^ to NH_3_ at more cathodic potentials. Possible reaction pathways are further proposed based on in situ Raman spectroscopy and electrokinetic analysis.

## Results and Discussion

### Characterization and Electrocatalytic NO_3_RR and NO_2_RR Performances of Cu_2_O NCs

**Figure 1:**
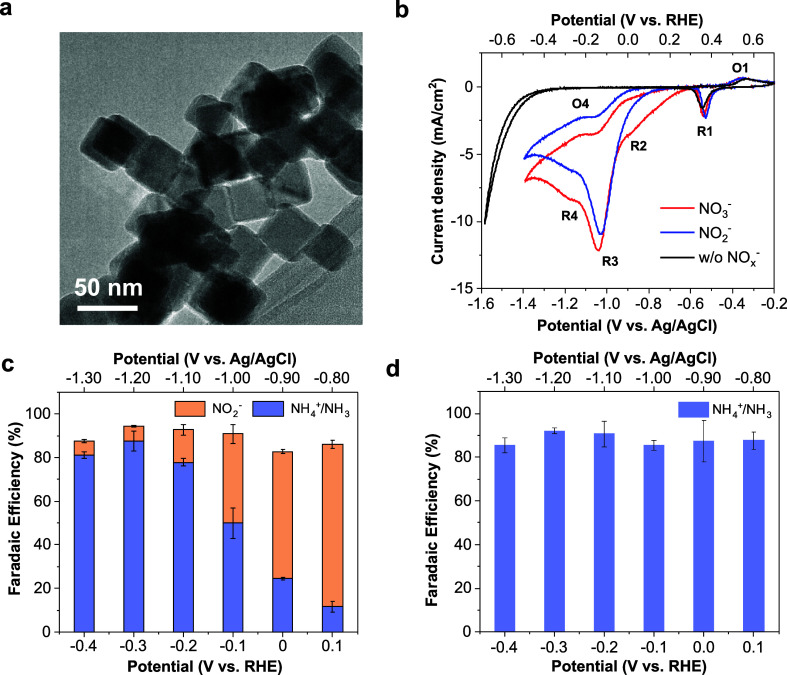
Characterization and electrocatalytic NO_3_RR and NO_2_RR performances of Cu_2_O NCs. (a) TEM image of the as prepared Cu_2_O NCs. (b) Linear scan voltammograms (LSV) of Cu_2_O NCs without adding NO_3_^–^/NO_2_^–^ sources (black) and in the presence of NaNO_3_ (red) and NaNO_2_ (blue), respectively, using a scan rate of 50 mV/s. (c,d) Potential-dependent FEs for NH_3_/NO_2_^–^ production during 2 h chronoamperometric NO_3_RR (c) and NO_2_RR (d). The error bars represent the standard deviation from three independent experiments. Experimental conditions: 0.1 M Na_2_SO_4_ electrolyte (pH 12) + 8 mM NaNO_3_ or NaNO_2_.

The Cu_2_O NCs were prepared via a wet-chemistry method, where copper sulfate was reduced by l-ascorbic acid in alkaline condition (see the Experimental section).^34^ The cubic morphology of the particles is confirmed by TEM (Figures 1a and S1a,b) and high angular annular dark field scanning transmission electron microscopy (HAADF-STEM, Figure S1c). The size of the Cu_2_O NCs is in the range of 30–40 nm (Figures 1a and S1a,c) and energy dispersive X-ray (EDX) elemental mapping images show uniform distribution of copper and oxygen (Figure S1c–e). The powder X-ray diffraction (PXRD) pattern (Figure S1f) reveals a crystallographic profile matching with only that of the cubic Cu_2_O phase (JCPDS no. 00-005-0667), which is also in accordance with the corresponding fast Fourier-transfer (FFT) pattern (Figure S1b) of the high-resolution (HR) TEM image (Figure S1a).

The NO_3_RR and NO_2_RR performances of the Cu_2_O NCs were initially evaluated in an H-type electrochemical cell, using 0.1 M Na_2_SO_4_ electrolyte with added NaNO_3_ or NaNO_2_ (8 mM) (Figures S2–S8 and Supporting Information Note 1). Despite the initial close-to-neutral pH of the electrolyte, ammonia formation and proton consumption occurring during NO_3_RR and NO_2_RR inevitably lead to a pH increase of the unbuffered bulk solution.^3,14^ In order to minimize the pH variation during the catalytic tests, the electrochemical behavior of Cu_2_O NCs was also investigated at alkaline pH (Supporting Information Note 1). An analogous electrochemical behavior was observed for all the explored pH conditions.

The cyclic voltammograms (CVs) of Cu_2_O NCs show the presence of quasi-reversible **R1**/**O1** peaks assigned to Cu(I)/Cu(0) transition, which are observed regardless of the addition of NO_3_^–^/NO_2_^–^ (Figure 1b). **R1** is followed by two irreversible reductive features (**R2** and **R3**) occurring at more cathodic potentials (Figure 1b, red). The **R2** wave exhibits an onset just after the **R1** peak and is absent when NO_3_^–^ is replaced by an equimolar amount of NO_2_^–^ in the electrolyte, suggesting the reduction of NO_3_^–^ to NO_2_^–^.^35,36^ The **R3** wave occurring at more negative potentials with a maximum current at ca. −0.14 V_RHE_, accounts for the further reduction of NO_3_^–^/NO_2_^–^ to products with lower oxidation state of nitrogen, as revealed by the similarity of the curves recorded in NO_3_^–^- and NO_2_^–^-containing electrolytes, respectively (Figure 1b, red and blue). The quasi-reversible **R4**/**O4** peak probably originates from the desorption/adsorption of *OH from metallic Cu,^37^ which are formed during the catalytic NO_3_^–^ and NO_2_^–^ reduction (see the Electrokinetic Analysis section for a detailed discussion). Moreover, a direct comparison with the polarization curve performed in the absence of NO_3_^–^/NO_2_^–^ (Figure 1b, black) indicates that the NO_3_^–^/NO_2_^–^ activation on Cu_2_O NCs occurs at considerably milder potentials than HER.

Electrolysis experiments were carried out at varying applied potentials and the spent electrolyte solution was analyzed by UV–vis spectroscopy to quantify the product distribution (Figures 1c and S9, S10). The accuracy of the UV–vis spectroscopy method is corroborated by ^1^H NMR analysis and the related ^15^N isotopic labeling experiments confirm that NO_3_^–^/NO_2_^–^ are the sources of the produced ammonia (Figures S11–S13, Supporting Information Note 1). At mild cathodic potentials, NO_3_RR catalyzed by Cu_2_O NCs results in a selective NO_3_^–^ conversion into NO_2_^–^, with a maximum faradaic efficiency (FE) of 75 ± 2% at 0.10 V_RHE_. However, NH_3_ formation progressively increases with overpotential and becomes the predominant product at the expense of NO_2_^–^ with a FE(NH_3_) > 80% between −0.20 and −0.40 V_RHE_. In particular, a maximum FE of 88 ± 5% and a molar yield rate of 45 ± 3 μmol h^–1^ cm^–2^ (or 7570 ± 473 μg_product_ h^–1^ mg_catalyst_^–1^), respectively, are obtained for ammonia formation at −0.30 V_RHE_, whereas the FE for NO_2_^–^ production is below 10% (Figures 1c and S14a,b).

The electrocatalytic performance of Cu_2_O NCs was also explored in the presence of an equimolar amount of NO_2_^–^. The catalyst exhibits close to or exceeds 90% FE for NH_3_ formation in a wide range of applied potentials, from 0.10 V to −0.40 V_RHE_ (Figure 1d). The higher FE(NH_3_) of NO_2_RR compared to those of NO_3_RR suggests faster kinetics for NO_2_^–^ activation and reduction by Cu_2_O NCs.

A comparison of the NO_3_RR performances of Cu_2_O NCs at different pH values shows analogous trends of activity and selectivity, with higher applied overpotential leading to higher FE(NH_3_) (Figures 1c, S4a, S5a,b, S14a,b and S15). A slight improvement of NH_3_ selectivity is observed at 0.0 and −0.1 V_RHE_ when the pH is increased to 12.9 (Figures 1c and S4a, S15a, Supporting Information Note 1). The Cu_2_O NCs also exhibit excellent NO_2_RR performances in both pH 12.9 and initially neutral electrolyte, leading to FE(NH_3_) > 90% in a wide potential range (Figures S4b,S5c,d and S16).

It is noteworthy that the Cu_2_O NCs exhibit better FE(NH_3_) compared to other Cu-based materials such as electropolished Cu foil^38^ and electrodeposited Cu catalyst^39^ under identical conditions (Figure S17). The excellent NO_3_RR performance and well-defined initial structures/compositions make our Cu_2_O NCs a good candidate for mechanistic insight.

### Correlation of the Oxidation State to Catalytic Performance

**Figure 2:**
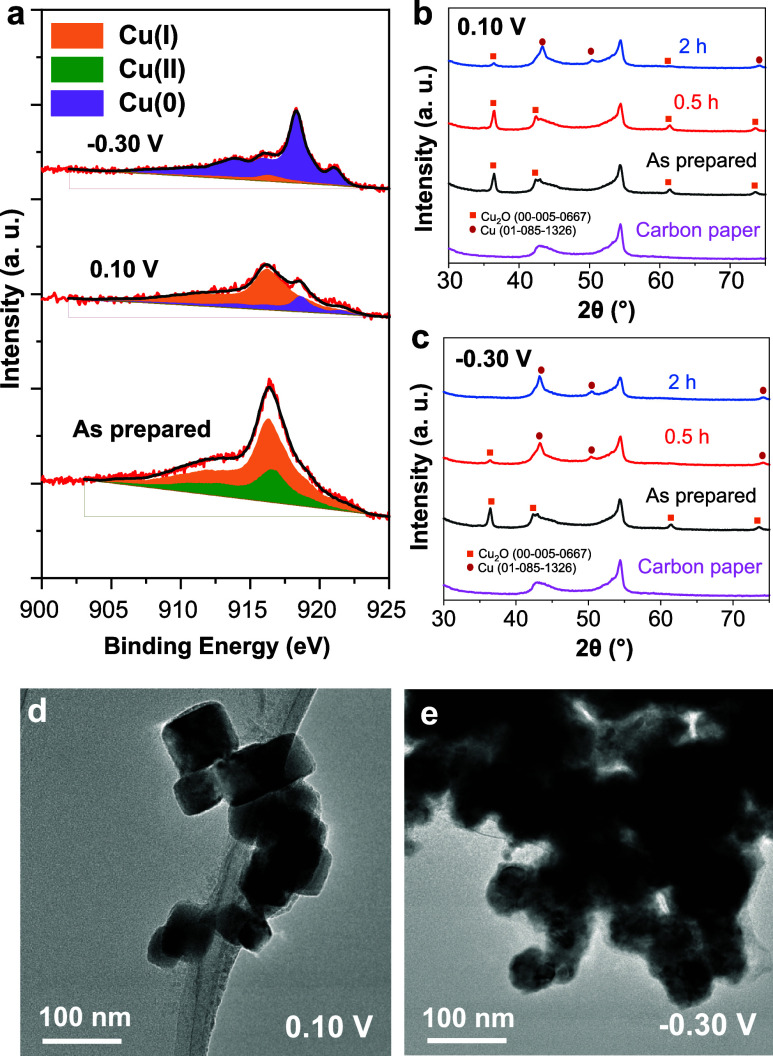
Quasi in situ and postreaction ex situ characterization of Cu_2_O NCs after NO_3_RR. (a) Quasi in situ XPS Cu LMM AES of Cu_2_O NCs after NO_3_RR (1 h) at different potentials. (b,c) Ex situ GI-PXRD of Cu_2_O NCs before and after NO_3_RR at 0.10 V_RHE_ (b) and −0.30 V_RHE_ (c). TEM of Cu_2_O NCs after NO_3_RR at 0.10 V_RHE_ (d) and −0.30 V_RHE_ (e). Electrolyte: 0.1 M NaSO_4_ + 8 mM NaNO_3_, pH 12.

In order to elucidate the evolution of structure and chemical state of Cu_2_O NCs during the electrocatalytic NO_3_RR, and to correlate these changes to the catalytic properties, we investigated the catalyst using complementary ex situ and in situ/operando techniques. Quasi in situ X-ray photoelectron spectroscopy (XPS) was employed to analyze the evolution of surface chemical state of the Cu_2_O NCs upon NO_3_RR in pH 12 electrolyte. Two potentials with distinct corresponding main NO_3_RR products: 0.10 V_RHE_ for NO_2_^–^ or −0.30 V_RHE_ for NH_3_ were chosen for the XPS measurements, respectively. According to the fitting results of Cu LMM X-ray Auger Electron spectra (XAES, Figure 2a, Table S1), Cu(I) still constitutes the majority of the species present in the catalyst near-surface region (70% content) after NO_3_RR at 0.10 V_RHE_ (Figure 2a, Table S1). The rest of the near-surface region is formed by metallic copper. On the other hand, at more negative potentials (−0.30 V_RHE_), where ammonia is predominantly formed, an almost complete reduction of Cu(I) to Cu(0) is observed on the catalyst surface, as indicated by the presence of 92% metallic copper (Figure 2a, Table S1). These findings indicate the presence of a mixture of Cu/Cu_2_O in the near-surface of the catalyst layer in a low overpotential regime, where NO_2_^–^ is the main product, while metallic Cu is predominant in the NH_3_ forming regime.

Consistent results were also obtained by grazing incidence X-ray diffraction (GI-XRD) measurements of Cu_2_O NCs after NO_3_RR. After 0.5 h of reaction at 0.10 V_RHE_, the catalyst is mostly in the form of Cu_2_O, whereas only a small peak at ∼43° corresponding to the metallic Cu(111) Bragg peak is observed. Longer reaction times (2 h) tend to generate more metallic and crystalline Cu species, as indicated by the growth of the Cu(200) Bragg peak at ∼50°, while simultaneously the intensity of the Cu_2_O-related Bragg peaks decrease (Figure 2b). After NO_3_RR at a more negative applied potential (−0.30 V_RHE_), the enhanced reduction of Cu_2_O ultimately leads to a significant formation of metallic Cu(0) already after short reaction times (0.5 h, Figure 2c), whereas the characteristic Bragg peaks corresponding to the Cu_2_O phase can be barely seen after 2 h (Figure 2c).

The transition from Cu_2_O NCs to metallic copper is paralleled by the change of morphology and crystallinity. After NO_3_RR at 0.1 V_RHE_, most of the Cu_2_O NCs are preserved (Figures 2d, and S18a–c). Some of the cubes are distorted, while the FFT patterns corresponding to both Cu_2_O and metallic copper are observed (Figure S19a–c). At −0.3 V_RHE_, the cubes are completely fractioned and changed to particulate aggregates (Figures 2e, S18d,e, S19d,e). The FFT patterns show the features of mixed polycrystalline Cu_2_O and Cu (Figures S18f and S19f).

**Figure 3:**
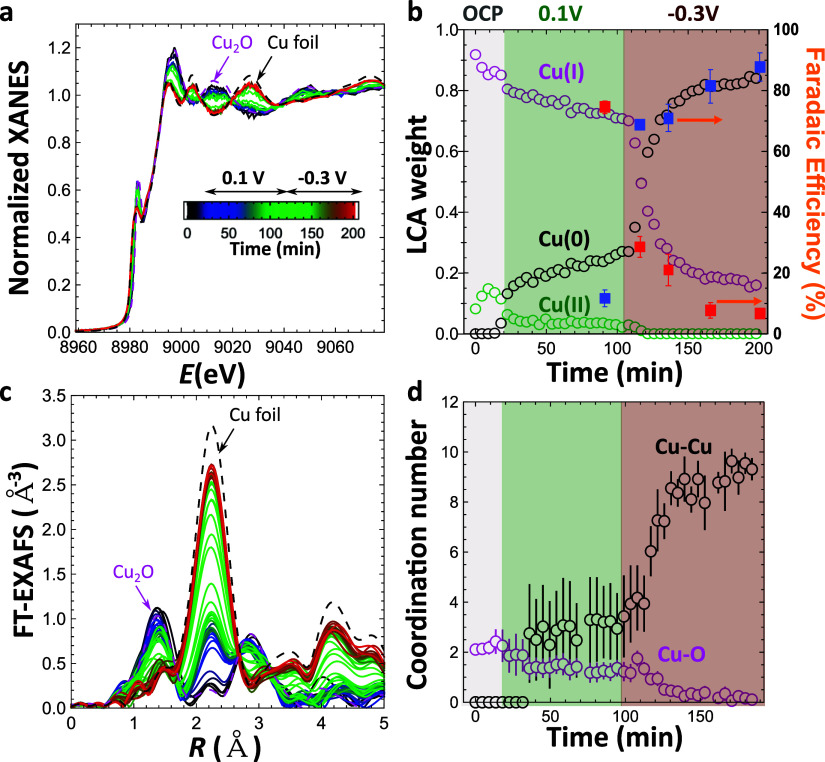
Operando XAS data for Cu_2_O NCs during NO_3_RR. (a) Normalized Cu K-edge XANES spectra collected at OCP, 0.10 V_RHE_ and −0.30 V_RHE_. (b) Linear combination analysis (LCA) results of the XANES spectra, showing the variation of the Cu oxidation state. The full squares indicate the time-dependent FE(NO_2_^–^) (red) and FE(NH_3_) (blue) obtained by NO_3_RR electrolysis experiments. The first data point is measured at 0.10 V_RHE_ for 2 h, while the other data points are measured at −0.30 V_RHE_ with the corresponding reaction times. The error bars are calculated based on three independent experiments. (c) Fourier-transformed (FT) Cu K-edge EXAFS spectra of Cu_2_O NCs at OCP, 0.10 V_RHE_, and −0.30 V_RHE_. (d) EXAFS fitting results showing the evolution of Cu–Cu and Cu–O coordination numbers. The error bars indicate the fitting uncertainty. Electrolyte: pH 12, 0.1 M Na_2_SO_4_ + 8 mM NaNO_3_.

To identify the Cu local atomic structure and chemical state during NO_3_RR, the dynamic structural changes of Cu_2_O NCs were investigated by means of operando XAS. Cu K-edge X-ray absorption near edge structure (XANES) data were analyzed using linear combination fitting (Figure 3a). Spectra for bulk CuO, Cu_2_O and Cu foil were used as references. In the NO_3_^–^ containing electrolyte (pH 12), the XANES data reveal that the as-prepared sample contains almost exclusively Cu(I) species. Nonetheless, we observed a gradual conversion of Cu(I) to Cu(II) species at open circuit potential (OCP), as also observed in the literature.^40^ This can be explained at least partially by the interactions with the X-ray beam and the presence of the electrolyte. In the regime of two-electron conversion of NO_3_^–^ to NO_2_^–^ at 0.10 V_RHE_ (Figure 1c), the Cu(II) species are reduced. However, the reduction of Cu(I) to Cu(0) is sluggish and in the bulk nearly 70% of Cu(I) remains after more than 1 h electrolysis (Figure 3b). When the potential is shifted to optimal conditions for NH_3_ production (−0.30 V_RHE_, Figure 1c), the remaining Cu(I) is rapidly reduced to Cu(0), resulting in more than 70% of copper being in the Cu(0) state after 15 min (Figure 3b). The fraction of the Cu(0) reaches ca. 85% after 1 h (Figure 3b), suggesting that our NCs remain partially oxidized. A direct comparison of the XANES results with the NO_3_RR selectivity data displays a strong correlation between the FE(NH_3_) and the formation of metallic Cu (Figure 3b). The extended X-ray absorption fine structure (EXAFS) analysis confirms the findings from XANES. Raw Cu K-edge EXAFS spectra are shown in Figure S20a. Figure 3c shows the Fourier transformed EXAFS spectra. EXAFS fitting results are summarized in Figures 3d and S20b. At OCP, the Fourier-transformed EXAFS spectra for our catalyst show two distinct peaks with a peak at 1.5 Å (phase uncorrected) corresponding to the Cu–O bond, while the peak at 2.9 Å we attribute to Cu–Cu distances in a Cu_2_O-like structure. Upon longer reaction times at 0.10 V_RHE_, the intensity of both peaks slowly decreases, while a new peak emerges at 2.1 Å, suggesting the formation of Cu–Cu distances in the metallic Cu phase. The Cu–Cu coordination number (CN) at 0.10 V_RHE_ is around 4, that is, approximately three times lower than the expected Cu–Cu CNs in bulk Cu metal. This suggests that only ca. one-third of the Cu species is in a metallic state, which agrees well with the XANES analysis results. At −0.30 V_RHE_, the Cu–Cu contribution increases rapidly, with a Cu–Cu CN reaching around 10 after 30 min. At the same time, the EXAFS features related to Cu_2_O are diminished. Hence, the EXAFS analysis results confirm that the Cu(I) species dominates in a potential region where NO_2_^–^ is the main product of NO_3_RR, while Cu(0) is the main species at more negative potential where the FE(NH_3_) is maximized.

The potential-dependent evolution of Cu_2_O NCs under NO_3_RR was further investigated using operando XAS data collected under a stepwise-applied potential in the range from 0.3 V to −0.5 V_RHE_, Figure S21). At 0.3 V_RHE_, where the **R1** peak emerged in the CV (Figures 1b and S21), the oxidation state of the catalyst still remains apparently unchanged, suggesting that the reduction of the catalyst bulk phase lags behind the reduction of the catalyst surface. The conversion of Cu(I) to Cu(0) starts between +0.1 V_RHE_ and −0.1 V_RHE_ and the reduction becomes faster at more negative applied potentials, where the increase of FE(NH_3_) was observed (Figures 1c and S21). Notably, the Cu_2_O NCs cannot be fully reduced even at more negative applied bias (−0.5 V_RHE_, Figure S21). An analogous behavior has been recently reported by us for similar Cu_2_O nanocubes during the electrocatalytic CO_2_ reduction.^41^ Notably, even the catalyst surface that is directly in contact to the electrolyte still contains a small amount of oxidized Cu (∼8%) when subjected to −0.3 V_RHE_ for 1 h according to our quasi in situ XPS data (Figure 2a, Table S1). Therefore, the observed changes in oxidation state of Cu and its concentration distribution during reaction predominantly reflects the intrinsic kinetic properties of the catalyst, although we cannot completely rule out that a minor fraction of the Cu_2_O are partially electrochemically inaccessible in the operando XAS experiments due to the higher catalyst loading on the porous gas diffusion electrode.

The Cu_2_O NCs display similar potential-dependent dynamic behavior at different electrolyte pH values (Figures S21–S24). Nonetheless, while the onset potential for Cu(I) reduction is almost pH-independent, a faster transformation of Cu(I) to Cu(0) is observed in alkaline electrolytes, consistent with the improvement of the apparent FE(NH_3_) at 0.0 and −0.1 V_RHE_ when the pH is increased (Figures 1c and S4a, S15a, S23).

Similar trends are obtained also from operando XAS measurements and quasi in situ XPS carried out under NO_2_RR conditions (Figures S25 and S26, Table S1), as well as TEM images after NO_2_RR (Figures S27 and S28). While the FE(NH_3_) of NO_2_RR was found to be less sensitive to copper oxidation state change, being >90% in a wide range of potentials (Figure 1d), the ammonia production rate rapidly increases as more metallic copper species are generated (Figure S14c,d).

### Electrokinetic Analysis

**Figure 4:**
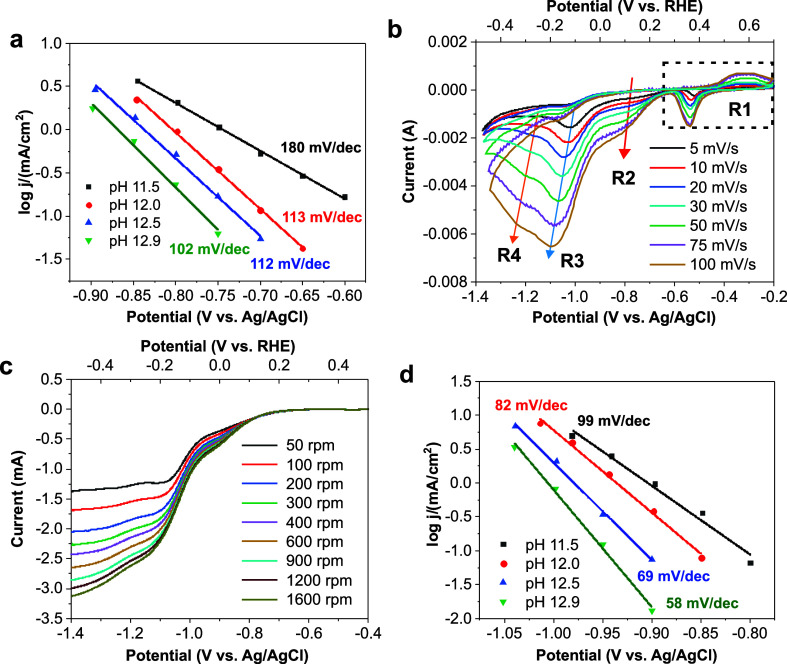
Electrokinetic analysis of NO_3_RR and NO_2_RR in alkaline conditions. (a) Tafel plots of Cu_2_O NCs at different pH (11.5–12.9) derived from staircase voltammetry. Electrolyte: 0.1 M Na_2_SO_4_ with 8 mM NaNO_3_ at different pH values. (b) Scan rate-dependent CVs of Cu_2_O NCs at pH 12.0. Typical reductive peaks are labeled. (c) Rotating rate dependent LSVs of Cu_2_O NCs on rotating disk electrodes at pH 12.0. (d) Tafel plots of Cu_2_O NCs at different pH (11.5–12.9) derived from staircase voltammetry. Electrolyte: 0.1 M Na_2_SO_4_ with 8 mM NaNO_2_ with different pH values.

To get deeper information on the underlying reaction mechanism of NO_2_RR/NO_3_RR, we performed a comprehensive electrokinetic study. Therefore, we recorded the polarization curves (Figure S29) of Cu_2_O NCs in the presence of 8 mM NaNO_3_ at pH values from 11.5 to 12.9, to determine the pH-dependent reaction kinetics. The quasi-reversible peaks before catalytic onset shift negatively with the electrolyte pH by ca. −80 mV/pH, implying that more than one proton is involved per Cu site during the Cu(I)/Cu(0) transition (Figure S29c,d). The Tafel slopes are in the range of 102–180 mV/dec (Figures 4a and S30), indicating that the first electron transfer for the activation of nitrate is the rate-determining step (RDS) in the investigated pH range at low overpotentials, according to the quasi-equilibrium Langmuir adsorption model assumption (Supporting Information Note 2). Moreover, from −0.75 to −0.84 V_Ag/AgCl_, the logarithm of the current density exhibits −0.70 ± 0.12 order on the pH values (Figure S31), indicating the RDS mostly consumes one proton, while water may partly be the hydrogen source (Supporting Information Note 4). Due to the nitrate adsorption equilibrium (Supporting Information Note 4), the reaction order of nitrate is less than one (0.64 ± 0.02), in the similar potential range (Figure S32). These results suggest that the RDS of NO_3_RR at low overpotential is a proton-coupled electron transfer (PCET) step.

To get mechanistic insight into the redox events occurring at higher overpotentials, scan rate dependence studies were performed in the presence of nitrate (pH 12) to complement the Tafel slope analysis (Figure 4b). The peak current of the quasi-reversible **R1** feature shows a linear dependence on the square root of the scan rate, both in a static solution and under stirring, while the currents are almost unchanged in the same scan rate (Figures 4b and S33, S34). This implies that the Cu(I)/Cu(0) redox transition is not restricted to the catalyst’s surface^42^ and is limited by the charge transfer in the bulk material.^43,44^ Unlike **R1**, both the **R2** and **R3** waves are irreversible and the scan rate dependence can be eliminated under stirring (Figures 4b and S33), manifesting that the peak current at high overpotentials is limited by mass transport of the reactant to the electrode–electrolyte interface.^42^ In a solution without forced convection, the shift of the peak potential (*E*_p_) linearly varies with the logarithm of the scan rate (log ν) based on the following equation (Supporting Information Note 3)^42,45^
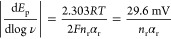
1where *n*_r_ is the number of transferred electrons in RDS (typically 1) and α_r_ is the transfer coefficient of RDS. For **R2**, a peak shift of 47.7 mV/dec (Figure S35a) leads to an α_r_ equal to 0.62, which is consistent with a Tafel slope value of ∼100 mV/dec regarding the first electron activation of nitrate as the RDS. By using the Nicholson–Shain equation (Supporting Information Note 3),^42,45^ the number of electrons of the **R2** wave is determined to be ∼1.7 (Figure S35b), suggesting rather a two-electron reduction of NO_3_^–^ to NO_2_^–^. A similar kinetic analysis for **R3** leads to an estimated number of transferred electrons equal to 5.5, which suggests a six-electron reduction of NO_3_^–^ to hydroxylamine (NH_2_OH), commonly reported as a key intermediate involved in the NO_3_RR route to ammonia on copper (Figure S36).^26,27,46^ Although the uncertainty in the electrode area determination due to the electrode porosity is expected to induce prominent errors in the *n* value, the ratio of *n*(R3)/*n*(R2) is always around 3 (2.9 ± 0.2). The reaction kinetics of NO_3_RR were also investigated using rotating disk electrode (RDE) and by analyzing the Koutecký–Levich plots^42^ (Figures 4c and S37, Supporting Information Note 3), the number of transferred electrons at 0.05 V and −0.30 V_RHE_ are calculated as 2.3 and 6.1, respectively, in accordance with the scan rate dependence analysis. Unlike **R1–R3**, the quasi-reversible **R4**/**O4** peak is observed only in the presence of NO_3_^–^/NO_2_^–^ (Figure 1b), and the peak current of **R4** shows linear dependence on the scan rate (Figure S38), corresponding to surface adsorbed species.^42^ We assigned **R4**/**O4** as desorption/adsorption of *OH on metallic Cu,^37^ which is generated during NO_3_^–^/NO_2_^–^ reduction.

In agreement with previous reports,^32,47^ NH_2_OH is not detected as a reaction product after NO_3_RR on Cu_2_O NCs in alkaline electrolytes, suggesting that it rapidly converts to ammonia under electrochemical reductive conditions. This is confirmed by the nearly quantitative ammonia formation [FE(NH_3_) > 90%] upon electrolysis carried out in the presence of 8 mM NH_2_OH (pH 12) at potentials from 0.1 to −0.3 V_RHE_ (Figure S39a). These results validate that NH_2_OH is a key intermediate species involved in the pathway for the multielectron conversion of NO_3_^–^/NO_2_^–^ to ammonia. Interestingly, the presence of NH_2_OH in solution induces a strong loss of reversibility of **R1** with an increased current density compared to the analogous voltammetric profiles in the presence of NO_3_^–^, which indicates a facile catalytic reduction of NH_2_OH on metallic Cu(0) (Figures S39b,c). However, the very large Tafel slope value (435 mV/dec, Figure S39d) suggests a chemical reduction of NH_2_OH on Cu(0) without involving interfacial charge transfer. According to the observed *OH desorption/adsorption **R4**/**O4** peaks (Figures 1b, 4b, and S38), we propose that the RDS of NH_2_OH reduction is the dissociation of NH_2_OH to adsorbed *NH_2_ and *OH, consistent with the observed apparent 6-electron reduction of NO_3_^–^, despite the final product being NH_3_ (Figures S36 and S37b).

The pH-dependence of NO_2_RR (from pH 11.5 to 12.9) was found to be similar to that of NO_3_RR (Figures 4d, and S40–S48). However, upon increasing the pH value, the Tafel slope gradually decreases from ∼100 to ∼60 mV/dec (Figures 4d and S41), suggesting a change of the mechanism from PCET activation of nitrite to a single-electron transfer pre-equilibrium step (PES) preceding a pure chemical RDS.^48^ This change is probably due to more available adsorbed hydrogen (*H) generated from the Volmer step in alkaline conditions (assumed as PES), with the chemical hydrogenation of nitrite becoming rate-determining (Supporting Information Note 2 and 4).^49^ Similar as NO_3_RR, one proton is involved in RDS or PES in total (Figures S31 and S42). Furthermore, both, the scan rate dependence and the RDE studies suggest the involvement of four electrons during NO_2_RR, in agreement with the generation of NH_2_OH as an intermediate species in the route to ammonia (Figures S46–S48, Supporting Information Note 4).

### Reaction Intermediates

**Figure 5:**
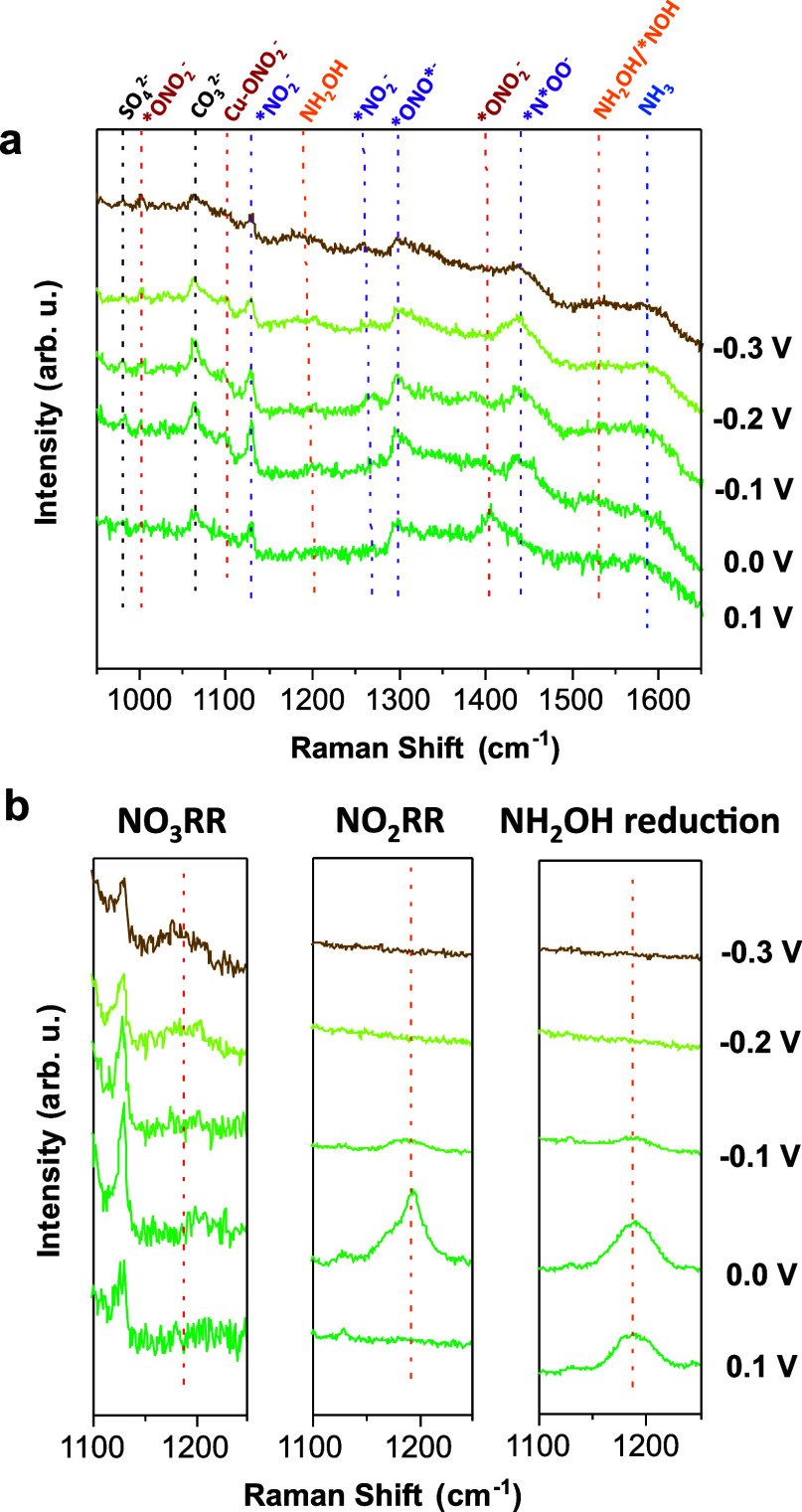
In situ Raman spectroscopy data of Cu_2_O NCs. (a) Enlarged in situ Raman spectra during NO_3_RR in the range of 950–1650 cm^–1^ Raman shift, with the applied potentials ranging from the 0.1 to −0.3 V_RHE_. Full spectra are shown in Figure S49a, Supporting Information. Electrolyte: pH 12, 0.1 M Na_2_SO_4_ + 8 mM NaNO_3_. The NO_3_^–^, NO_2_^–^, NH_2_OH and NH_3_ related peaks are labeled with red, violet, orange, and blue dotted lines, respectively. The asterisk denoted the atoms adsorbed on the catalyst surface. Peaks at 983 and 1065 cm^–1^ correspond to SO_4_^2–^ and CO_3_^2–^ in solution (labeled with a black dotted line),^34,36^ respectively, as they always appeared in the electrolyte containing Na_2_SO_4_ (pH 12). (b) In situ Raman spectra in the range of 1100–1250 cm^–1^ during NO_3_RR (left, pH 12, 0.1 M Na_2_SO_4_ + 8 mM NaNO_3_), NO_2_RR (middle, pH 12, 0.1 M Na_2_SO_4_ + 8 mM NaNO_2_), NH_2_OH reduction (right, pH 12, 0.1 M Na_2_SO_4_ + 8 mM NH_2_OH). The NH_2_OH related peaks are labeled with orange dashed lines. The intensity of NO_3_RR Raman spectra is manually increased five times for clarity.

To verify the proposed formation of reaction intermediates during NO_3_RR and NO_2_RR, we performed in situ Raman spectroscopy of the Cu_2_O NCs (Figures 5 and S49–S51). The primary goal was to prove the formation of the NH_2_OH intermediate and therefore, we additionally performed control in situ Raman spectroscopy experiments using NH_2_OH as reactant in the absence of nitrate/nitrite (Figure S52) and also under HER conditions (Figure S53). For all cases (Figures S49b, S50b, S52b, and S53), only Cu_2_O characteristic peaks (415, 530, and 625 cm^–1^)^34^ and peaks related to the G-band of the carbon substrate (1580 cm^–1^)^34^ can be observed under OCP and at potentials more positive than 0.3 V_RHE_. Cu_2_O characteristic peaks started to disappear at a cathodic potential of 0.3 V_RHE_, which agrees well with the Cu(I)/Cu(0) redox peaks in the CVs (Figure 1b). At more negative potentials than 0.1 V_RHE_, the oxide-related spectroscopic region (300–800 cm^–1^) significantly changed, showing broad features that suggest the presence of adsorbed hydroxyl ions on metallic Cu and remnant disordered Cu_2_O.^50,51^ The oxide features can still be observed when the potential is decreased to −0.3 V_RHE_, which agrees well with our findings based on operando XAS (Figure 3b). Note that upon the reduction of the Cu_2_O nanocubes, the intensity of the Raman signals is significantly enhanced due to surface plasmonic effects of nanostructured metallic Cu.^34^

To determine the Raman bands induced by the NH_2_OH intermediate and its reduction to NH_3_, we performed in situ Raman spectroscopy in a NH_2_OH-containing electrolyte (Figures 5b and S52) and identified the peaks at 870, 1190, and 1526 cm^–1^ corresponding to NH_2_ rocking, symmetric wagging vibration of NH_2_ and antisymmetric N–H bending in NH_2_OH^26,52^ respectively, in the potential regime of 0.3 to −0.1 V_RHE_. Notably, we also identified several peaks related to NH_3_, including NH_3_ rocking (785 cm^–1^),^36^ symmetric bending vibrations of the HNH (1320 and 1371 cm^–1^),^53^ ν(T_4_) antisymmetric NH_2_ deformation of NH_4_^+^ (1455 cm^–1^),^26,54^ and the antisymmetric bending vibration of the HNH of NH_3_ (1550 and 1590 cm^–1^).^36,53^ A further decrease in the potential results in attenuated peak intensity, probably due to much faster conversion rate of NH_2_OH and production rate of NH_3_. The in situ Raman results are consistent with our electrochemical kinetic data, indicating the reduction of NH_2_OH to NH_3_ on metallic Cu.

In situ Raman spectra acquired during NO_3_RR between 950 and 1650 cm^–1^ served to monitor the most important adsorbates under electrocatalytically relevant potentials between 0.1 and −0.3 V_RHE_ (Figure 5a). Adsorption of nitrate is evidenced between 0.1 and −0.3 V_RHE_ from Raman peaks at 1005 cm^–1^, corresponding to the NO stretching vibration from the unidentate nitrate (*ONO_2_^–^).^53^ The peak at 1098 cm^–1^ that resembles those observed for copper-nitrate complexes also implies interaction between copper and nitrate.^55^ Broad peaks at 1400 cm^–1^ are assigned to antisymmetric vibrations of the NO_2_ group in nitrate.^36^

Furthermore, we have identified adsorbed NO_2_^–^ species between 0.1 and −0.3 V_RHE_ following the Raman bands at 1128 and 1266 cm^–1^.^36,53^ These bands are typically assigned to the symmetric and antisymmetric stretching vibration of the adsorbed NO_2_ in a nitro configuration (*NO_2_^–^). Additionally, the Raman band at 1298 cm^–1^ is typically assigned to the chelating nitrito configuration (*ONO*^–^), while the peak at 1440 cm^–1^ is related to bridging nitro configuration on the Cu surface (*N*OO^–^).^36,53^ Concurrently with the NO_2_^–^-related bands, broad features between 1500 and 1630 cm^–1^ appeared cathodically at 0.1 V_RHE_. These broad peaks might consist of several peaks related to intermediates or products of NO_2_^–^ reduction, such as ν(N=O) of HNO* (1530 cm^–1^),^36^ antisymmetric bending vibration of the HNH of NH_3_ (1550, 1590 cm^–1^),^36,53^ and O–H bending of water (1610 cm^–1^).^56^ The broad peaks from 2000 to 2100 cm^–1^ indicate that *NO species might be involved in NO_3_RR (Figure S49c).^57^

Most importantly, we identified weak and broad Raman bands assigned to the N–H bending (1526 cm^–1^) and the NH_2_ symmetric wagging vibration of NH_2_OH (1190 cm^–1^)^26,54^ during NH_3_ formation below 0.0 V_RHE_, proving the presence of NH_2_OH intermediate during NO_3_RR to NH_3_ (Figure 5a,b). Qualitatively, we found that the relative spectral weight of the nitrite-related bands decreases below −0.1 V_RHE_, while the intensity of the NH_2_OH- and NH_3_-related bands increases below 0.0 V_RHE_, consistent with NO_2_^–^ as the main product in the low overpotential region, while NH_3_ is observed as the main product at high overpotential.

In terms of NO_2_RR (Figures S50–S51), nitrite-related peaks at 1128 cm^–1^ were observed.^53^ Small broad peaks from 1280 to 1410 cm^–1^ might consist of several peaks related to adsorbed *ONO*^–^ (1298 cm^–1^, chelating nitrito)^36^ and ammonia (1320 and 1371 cm^–1^, symmetric bending vibrations of the HNH).^36,53^ The overlapping peaks at 1440 and 1458 cm^–1^ are related to bridging nitro (*N*OO^–^) and NH_4_^+^,^26,36,53,54^ respectively, according to Raman spectroscopy of NO_3_RR and NH_2_OH reduction. ν(N=O) of HNO* (1530 cm^–1^)^36^ and antisymmetric bending vibration of the HNH of NH_3_ (1550, 1590 cm^–1^)^36,53^ appeared as well. These findings suggest that NO_2_RR and NO_3_RR share common intermediates and reaction steps. Notably, the peaks related to NH_2_OH at 1190 cm^–1^ emerge at more positive potential and they are more obvious under NO_2_RR condition (Figure 5b), comparing to those of NO_3_RR, manifesting more facile reaction kinetics for NO_2_RR.

### Active Species and Reaction Mechanisms

**Figure 6:**
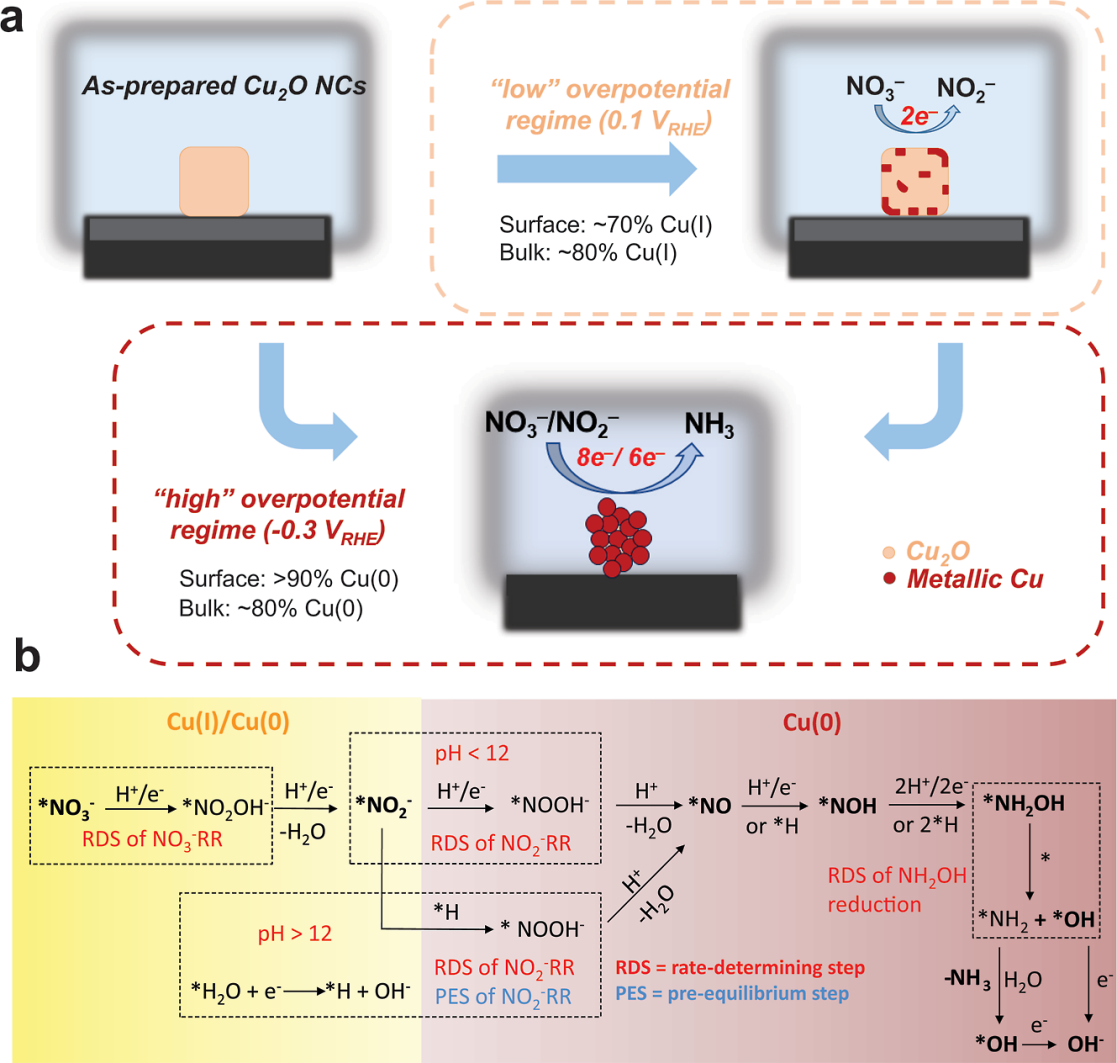
Active species and possible reaction mechanisms. (a) Schematics of the structural and oxidation state change of the catalyst under NO_3_RR/NO_2_RR conditions. (b) Proposed mechanisms for NO_3_RR/NO_2_RR to ammonia over Cu_2_O NCs. The species that been determined based on experimental evidence (either detected by in situ Raman or deduced from electrokinetic analysis) are in bold. The left side (yellow background) correspond to reaction conditions where Cu(I)/Cu(0) species are present, while on the right-hand side (red background) the catalyst is mostly metallic.

Based on the aforementioned experimental results, the evolution of the catalyst and the active species can be schematically depicted in Figure 6a. The overall eight-electron conversion of NO_3_^–^ to NH_3_ takes place via two main potential-dependent steps. The initial two-electron activation and reduction of NO_3_^–^ to NO_2_^–^ occurs in a potential region (ca. 0.1 V_RHE_) where the original cuprous(I) oxide layer in the as-prepared Cu_2_O NCs is partially and slowly reduced to metallic Cu(0), while remaining predominantly Cu(I) in both the near-surface and in the bulk of the sample. Hence, the catalyst surface features a mixed Cu/Cu_2_O phase composition at mild applied voltages, where both, Cu(I) and Cu(0) species can act as active sites for NO_3_^–^ adsorption and activation toward NO_2_^–^ formation.

At larger overpotentials (ca. −0.3 V_RHE_), NH_3_ is produced with high efficiency and selectivity by further NO_2_^–^ reduction (Figure 6a). In this applied potential range, the catalyst is mainly metallic, whose formation rate from the initial Cu_2_O precatalyst strongly correlates with the FE(NH_3_) formation (Figure 3b). While we cannot rule out that the small amount of residual Cu(I) species may play some roles in the process, these data indicate that metallic Cu(0) represents the main catalytic sites for the NO_3_^–^/NO_2_^–^ conversion to NH_3_. We hypothesize that in addition to chemical reasons, the enhanced electrical conductivity of metallic copper is beneficial for the multielectron reduction of NO_3_^–^/NO_2_^–^, thus favoring the selectivity toward NH_3_. In this regard, the observation that increasing overpotentials and electrolysis times lead to an improved NH_3_-to-NO_2_^–^ selectivity is consistent with a sluggish reduction of the initial semiconducting cuprous(I) oxide centers to the active Cu(0). Notably, a rapid increase of the Cu–Cu coordination number observed during operando XAS (Figure 3d) is indicative of severe surface restructuring and particle aggregation occurring during NO_3_RR to NH_3_. An analogous behavior has been recently reported for single-atom Cu precatalysts undergoing a dynamic reconstruction driven by the applied potential during NO_3_RR to form the real Cu(0) NP catalyst.^58^

We note that previous studies led to contrasting conclusions about the oxidation states and active phases of copper-based catalysts for NO_3_RR.^21,31−33^ Such discrepancies may not only originate from the different nature of the catalysts (or precatalysts), but are also related to the interpretation of the data based in many prior examples on an ex situ postreaction characterization of the catalysts, usually influenced by the rapid oxidation of surface Cu(0) back to Cu(I) upon even short time exposures to ambient conditions. A single characterization technique, even under working conditions, also does not provide a complete view of the chemical and structural evolution of complex electrocatalysts. Thus, our data and conclusions highlight the importance of combining various in situ/operando techniques to reveal the evolution of electrocatalysts and formation the catalytically active species upon exposure to different chemical environments, different applied potentials and during various reaction times. Although some reports previously showed the existence of a mixed Cu/Cu_2_O under NO_3_RR conditions,^31^ we unambiguously show here that the emergence of metallic Cu correlates with NH_3_ selectivity and activity, while Cu(I) species can only catalyze the two-electron reduction of nitrate.

Based on the results of the electrokinetic analysis and spectroscopic data, we propose that the reduction of NO_3_^–^ to NH_3_ proceeds via the main pathways depicted in Figure 6b, and Supporting Information Note 4. The RDS of the NO_3_RR is first PCET activation of adsorbed NO_3_^–^, while the second PCET further generates *NO_2_^–^. These two steps can be promoted by either Cu(I) or Cu(0) species while further reduction of *NO_2_^–^ requires Cu(0). A recent work^59^ demonstrated that the conversion of NO_3_^–^ to NO_2_^–^ can be easily realized by the spontaneous redox reaction between metallic Co and NO_3_^–^. To check whether the generated metallic Cu can be oxidized by nitrate in our case, we performed similar in situ Raman experiments (Figure S54). We first applied −0.3 V_RHE_ for 15 min in 0.1 M Na_2_SO_4_ electrolyte at pH 12 to reduce the Cu_2_O to metallic Cu. After this prereduction, we quickly added 0.5 M NaNO_3_ to the 0.1 M Na_2_SO_4_ electrolyte, resulting in 8 mM NaNO_3_ in the electrolyte and monitored the evolution of the Raman bands under OCP. We observed the characteristic Raman peaks related to Cu_2_O quickly emerge and stabilize after 1 min in both, the nitrate-containing (Figure S54a) and nitrate-free electrolyte (Figure S54b). We also did not detect any NO_2_^–^ by UV–vis spectrometry for 4 min after adding NO_3_^–^. Therefore, we conclude that the spontaneous chemical reaction between Cu and nitrate does not happen in our catalyst.

The RDS of NO_2_RR can either be a PCET reduction of *NO_2_^–^ or the hydrogenation of *NO_2_^–^ via *H, which is probably generated from water dissociation PES, depending on the pH values. The exposure of metallic Cu(0) sites on the surface may favor the *H adsorption, thus enabling the hydrogenation step.^49,60^ According to the number of transferred protons and electrons estimated by the electrokinetic analysis, *NO_2_OH^–^ and *NOOH^–^ can be proposed as probable intermediate adsorbed species formed after activation of nitrate and nitrite, respectively. These intermediates were also suggested by several computational studies.^61,62^

Finally, in agreement with the determination of six electrons for NO_3_RR and four electrons for NO_2_RR by scan-rate dependence and RDE studies, we propose the formation of NH_2_OH as a possible intermediate involved in the route to NH_3_ in alkaline electrolyte,^22,26,31,63^ which is also confirmed by in situ Raman spectroscopy (Figure 5). The reduction of NH_2_OH to NH_3_ likely occurs on metallic Cu via a chemical step which involves the dissociation of *NH_2_OH to form the adsorbed *NH_2_ and *OH. The *NH_2_ intermediate can further react with water to produce ammonia and generate another *OH, while the *OH is rapidly reduced to release OH^–^. The reduction of NO_2_^–^ to NH_2_OH possibly involves *NO, *HNO as the intermediates.

It should be however noted that the mechanistic insights obtained here are only applicable to the NO_3_RR in an unbuffered alkaline electrolyte, and that alternative mechanism might be possible when working under acidic media (Figure S55, and Supporting Information Note 4).

## Conclusions

In summary, we have presented a detailed picture for a fundamental understanding of the ammonia electrosynthesis reaction from NO_*x*_^–^ sources on copper, taking well-defined Cu_2_O nanocubes as model precatalysts. Combining electrochemistry and in situ/operando spectroscopy, we found a direct correlation between the potential- and time-dependent changes of the chemical state of the catalyst during NO_3_RR/NO_2_RR and the catalytic selectivity, providing insights into the main active species involved in the process. The sluggish reduction of Cu(I) to metallic Cu at low overpotentials results in low NH_3_ production rates and efficiencies, suggesting that the Cu(I) species can only catalyze the two-electron NO_3_^–^-to-NO_2_^–^ reduction. On the other hand, the increased formation rate of Cu(0) at high overpotentials strongly correlates with improved NH_3_ Faradaic efficiency and/or yield rate, thus reflecting the crucial role of metallic Cu toward stepwise multielectron reduction of NO_3_^–^ and NO_2_^–^ to NH_3_. We proposed an electrokinetic model consistent with the experimental data, highlighting the main pathways possibly leading to NO_2_^–^ and NH_3_, respectively. Our data suggest that the RDS of NO_3_RR involves a PCET activation of nitrate, while the rate of NO_2_RR is limited by either a PCET activation or a direct hydrogenation of nitrite, depending on the electrolyte’s pH. We also proposed that the final step leading to NH_3_ is limited by a chemical reaction involving NH_2_OH.

This study highlights the importance of using in situ/operando spectroscopic tools to unravel the chemical evolution process and active species of the catalyst at different potentials and reaction times. The mechanistic understanding provided here for the ammonia electrocatalytic synthesis offer several clues for future advanced catalyst design. In particular, we have demonstrated that it is crucial to stabilize copper in the metallic state under lower overpotential as well as to facilitate the generation and stabilization of adsorbed *H.

## Methods and Experimental Details

### Synthesis of Cu_2_O NCs

The Cu_2_O NCs were synthesized by using a slight modification of a previously reported surfactant-free procedure.^34^ Briefly, 1 mL of a 0.1 M CuSO_4_ solution (prepared from CuSO_4_·4H_2_O, Sigma-Aldrich, 99.995% trace metals basis) was added to 91.5 mL of Milli-Q water (18.2 MΩ) and the mixture was stirred for 10 min. Then, 1 M sodium hydroxide (3.5 mL, Alfa Aesar, >97%) was poured to the mixture under stirring, followed after 10 s by the addition of a freshly prepared 0.25 M l-ascorbic acid solution (4 mL, Sigma-Aldrich, reagent grade). The mixture was further stirred at room temperature for 15 min. The particles were retrieved by centrifugation and washed with an ethanol/water mixture (1:1) three times and ethanol one time. The solid sample was finally dispersed in 6 mL ethanol and stored in the fridge. Inductively coupled plasma mass spectrometry (ICP-MS, iCAP RQ, Thermo Fisher Scientific) data indicated that the mass concentration of catalyst suspension is 1.2 ± 0.1 mg/mL.

### X-Ray Diffraction

X-ray diffraction (XRD) was performed on a Bruker D8 Advance diffractometer equipped with a Cu source, a primary Goebel mirror and a secondary equatorial Soller slit as well as an energy-dispersive detector operated in 0D mode. To investigate the electrode samples of this study, grazing incident mode was applied with an incident angle of 1° and a measurement range of 10–85° with a step size of 0.04°. All the manipulations were conducted with minimal but inevitable air exposure, which may cause partial reoxidation of the surface but not of the bulk. The sample was placed on a special sampler holder having an airtight dome to minimize air contact during the measurement. Furthermore, a low-background silicon plate was used as sample support in the holder. Immediately after the experiments, the catalyst containing carbon paper electrode (geometric active area of 0.5 × 0.5 cm) was removed from the reaction vessel and rinsed thoroughly with Milli-Q water. After carefully drying with an Ar flow, the electrode was fixed to the sample holder with the help of some stripes of Kapton tape. To minimize the contact to air, the holder was transferred to a N_2_-filled glovebox as fast as possible. With the closed dome the sample was measured immediately afterward. Between the end of the electrolysis experiment and the transfer of the sample into the glovebox, ca. 5–10 min passed.

### Transmission Electron Microscopy

The size and morphology of the Cu_2_O NCs were determined by transmission electron microscopy (TEM) using an FEI Talos F200X microscope. TEM samples were prepared by placing 10 μL of the as-synthesized Cu_2_O NCs suspensions (with a five-time dilution with pure ethanol) on a nickel grid (400 mesh with lacey carbon film, PLANO GmbH) and allowing it to dry in air overnight. For investigating the catalysts after reaction, the carbon paper electrodes that are loaded with the catalyst were initially subjected to 2 h electrolysis at a certain potential. Then the electrodes are quickly washed in ultrapure water and dried under nitrogen flow. After that, the dry electrodes are put in ethanol and sonicated for 1 h. Finally, 10 μL suspensions are drop-casted on nickel grids for the TEM measurements.

### Quasi In Situ X-Ray Photoelectron Spectroscopy

Quasi in situ XPS measurements were carried out using a sample to analyzer angle of 90°. The system employed was a commercial hemispherical analyzer (SPECS GmbH, Phoibos 150 equipped with a MCD-9 Detector) using a pass Energy of 15 eV and a monochromatic XRM X-ray source (SPECS GmbH) utilizing an Al anode (*E* = 1486.7 eV). A source power of *P* = 300 W was used. The electrochemical cell employed was directly attached to the ultrahigh vacuum (UHV) system, allowing for a sample transfer from electrochemical conditions to the UHV in a protective Ar atmosphere.^64^

### Operando X-Ray Absorption Spectroscopy

Operando XAS measurements were performed at the Cu K-edge (8979 eV) in fluorescence mode at the SOLEIL SAMBA beamline. Supporting measurements at different applied potentials were also done at ALBA CLAESS beamline.

For experiments at SOLEIL, a Si(220) monochromator was used for energy selection. Intensity of incident X-ray radiation was measured with an ionization chamber filled with a 50/50 mixture of N_2_ and He. Fluorescence from the sample was detected using an energy-selective 35-channel Ge detector. For experiments at ALBA, a Si(111) monochromator was used, and 6-channel energy-selective silicon drift detector was used to collect fluorescence data.

For operando XAS measurements, the samples were deposited on carbon paper, which acted both as a working electrode and X-ray window in our in-house built single compartment electrochemical cell.^65^ Pt mesh was used as a counter electrode, and a leak-free Ag/AgCl electrode was used as a reference electrode. The applied potential was controlled by a BioLogic potentiostat. During the experiment, continuous circulation of the electrolyte was ensured by a peristaltic pump, and Ar was bubbled through the cell.

XAS data extraction, alignment, and normalization were done using the *Athena* software.^66^ Linear combination fitting of XANES spectra was done by using an in-house developed set of *Wolfram Mathematica* scripts. EXAFS data fitting was done using the FEFFIT code.^66^ Fitting was carried out in the R-space in the range between 1.0 and 2.8 Å (between 1.0 and 2.0 Å for the as-prepared fully oxidized samples). Fourier transform for EXAFS data fitting was carried out in the k-range between 3.0 and 10.0 Å^–1^. The EXAFS signal was modeled as sum of Cu–O and Cu–Cu contributions, where the corresponding bond lengths, coordination numbers, and disorder factors were treated as fitting parameters. For the as-prepared fully oxidized samples the Cu–Cu bond contribution was not included. An additional fitting parameter was the correction to the photoelectron reference energy value (Δ*E*_0_) value. The amplitude reduction factor (*S*_0_^2^ factor) was obtained by fitting the EXAFS spectra for Cu_2_O and Cu foil references (the obtained values are 0.7 and 0.92, respectively). The *R*-factors, characterizing the EXAFS fit quality, were lower than 0.5% for the all nearly phase-pure spectra (i.e., spectra, corresponding to the fully oxidized as-prepared samples, and to nearly fully reduced samples after prolonged exposure to NO_3_RR). For samples in the intermediate state the *R*-factors were higher, but still below 3%. The increased *R*-factor in this case is due to the presence of a remaining Cu_2_O-like phase, where the Cu–Cu bond from the second coordination shell (not included in the fit) partially overlaps with the contribution of the first shell Cu–Cu bond in metallic Cu phase.

### In Situ Raman Spectroscopy

The in situ Raman spectra were obtained by using a Renishaw (InVia Reflex) Raman instrument with confocal Raman microscope. To perform the in situ experiments in an electrolyte, a water immersion objective with a long working distance (Leica microsystems, 63×, numerical aperture of 0.9) was chosen. During the experiments, the objective was protected from the electrolyte by a Teflon film (DuPont, 0.013 mm thickness). Fifteen μL of water was used between the film and the objective to match the refractive index, which ensures efficient collection of the Raman signal. The objective with long working distance is necessary to avoid diffusion hindrance problems during the Raman measurements. A 633 nm laser was employed and the laser power was 5%. The extended mode was chosen and the acquisition time was 10 s. A Si wafer (520.6 cm^–1^) was used to calibrate the Raman frequency before the experiment. The electrochemical measurements were performed in a home-built spectroelectrochemical cell made of Teflon and controlled by a Biologic SP-240 potentiostat. The cell was equipped with a reference electrode (leak-free Ag/AgCl, Alvatek), a counter electrode (Pt ring), and a working electrode with the catalyst drop-casted on carbon paper. Typically, a 12.5 mL electrolyte was filled in the cell, with continuous Ar bubbling during the experiments, to remove oxygen and alleviate mass transport issues. For each applied potential, the spectra were collected after 30 s and the final data were obtained using the averaged results of 5–8 tests to improve the quality.

### Electrochemical Experiments

All the electrochemical experiments were carried out by using an Autolab potentiostat (PGSTAT 302N) and a custom-made H-type electrochemical cell, whereby the cathodic and anodic compartments were separated by an anion exchange membrane (Selemion AMV, AGC Inc.). A platinum gauze (MaTecK, 3600 mesh cm^–2^) and a leak-free Ag/AgCl electrode (LF-1, Alvatek) were used as the counter and the reference electrodes, respectively. In a typical experiment, each compartment was filled with 18 mL 0.1 M Na_2_SO_4_ electrolyte (anhydrous, 99.99%, Suprapur). For nitrate electroreduction tests, the cathodic electrolyte solution contained 8 mM NaNO_3_ (112 ppm of NO_3_–N, Sigma-Aldrich, ReagentPlus, ≥99.0%). For ^15^N labeling nitrate electroreduction experiments, equimolar concentrations of Na^14^NO_3_ and Na^15^NO_3_ (Sigma-Aldrich, ≥98 atom % ^15^N, ≥99%) were employed, respectively. Before each experiment, both, the anodic and cathodic solutions were deaerated by continuously bubbling Ar (grade 6.0, 99.9999%) with a 20 mL min^–1^ flow rate (Bronkhorst). During the chronoamperometric measurements, a constant Ar flow (20 mL min^–1^) was kept to maintain the inert atmosphere. The potential values were reported vs Ag/AgCl electrode (V_Ag/AgCl_) or vs the reversible hydrogen electrode (V_RHE_) for experiments carried out in unbuffered near-neutral or alkaline electrolytes, respectively. For all the electrolytes, the cyclic voltammograms (CV) were recorded at a scan rate of 50 mV s^–1^ and five consecutive cycles between −0.20 V and −1.40 V_Ag/AgCl_ were performed to achieve steady-state polarization curves and to achieve a stable and reproducible state of the electrocatalyst prior to the catalytic testing. For the electrolysis experiments, a linear sweep voltammogram (5 mV s^–1^) was performed from −0.30 V_Ag/AgCl_ to the cathodic potential followed by chronoamperometry at the latter potential for 2 h. The LSV and electrolysis experiments are applied with 800 rpm stirring using a stirring bar.

For NO_2_RR experiments, all the steps are similar as those of NO_3_RR, except 8 mM NaNO_2_ (99%, for analysis, Sigma-Aldrich) is the reactant instead of NaNO_3_. For ^15^N isotope labeling experiments, 8 mM Na^15^NO_2_ (98 atom % ^15^N, 95% chemical pure, Sigma-Aldrich) is used.

Tafel plots were recorded using staircase voltammetry. The electrode was preactivated in pH 12, 0.1 M Na_2_SO_4_ + 8 mM NaNO_3_ for 1 h, at −1.20 V_Ag/AgCl_ (−0.30 V_RHE_). Then the activated electrode was measured in a fresh electrolyte with pH values ranging from 11.5 to 12.9, containing 8 mM NaNO_3_/NaNO_2_/NH_2_OH. The electrolyte pH was tuned by adding 4 M NaOH, and the values were monitored by a VWR pH1100L pH meter. The potential was ranging from −0.60 to −1.40 V_Ag/AgCl_, with a ramping step of 50 mV. For each data point, the potential was held for 60 s, and then the current was recorded. The electrolyte is stirred using a stirring bar at 800 rpm.

For acquisition of scan rate dependence current–potential data of both NO_3_RR and NO_2_RR (pH 12), the electrode was preactivated in pH 12, 0.1 M Na_2_SO_4_ + 8 mM NaNO_3_ for 1 h, at −1.20 V_Ag/AgCl_ (−0.30 V_RHE_). The activated electrode was then put in a fresh pH 12, 0.1 M Na_2_SO_4_ + 8 mM NaNO_3_/NaNO_2_ electrolyte. The CV curves were recorded between −0.20 and −1.40 V_Ag/AgCl_, starting from −0.20 V_Ag/AgCl_. The scan rate was sequentially set as 5, 10, 20, 30, 50, 75, and 100 mV/s. For the CV curves recorded without stirring, after each measurement, a 1000 rpm stirring was applied for 1 min to remove the concentration gradient of the reactants/products near the electrode caused by the reaction. In this way, the initial state of the solution can be restored. After collecting the CV curves recorded without stirring, the electrode was put in a fresh electrolyte, following the same procedure to acquire the CV curves recorded with stirring (800 rpm).

For RDE experiments, the electrode was also preactivated in pH 12, 0.1 M Na_2_SO_4_ + 8 mM NaNO_3_ for 1 h, at −1.20 V_Ag/AgCl_ (−0.30 V_RHE_), with a rotating speed of 900 rpm. The activated electrode was then put in a fresh pH 12, 0.1 M Na_2_SO_4_ + 8 mM NaNO_3_/NaNO_2_ electrolyte. The LSV curves were recorded between −0.40 to −1.40 V_Ag/AgCl_, starting from −0.40 V_Ag/AgCl_. The rotation rate was sequentially set as 50, 100, 200, 300, 400, 600, 900, 1200, and 1600 rpm. After each measurement, a 900 rpm rotation was applied for 1 min to restore the initial state of solution.

Note that for all the measurements of the electrokinetic studies, the values of the solution resistance were measured by the current-interruption method, and then the CV curves, LSV curves, or steady state *J*–*V* curves were manually *iR* corrected.

For the electrode preparation, 0.98 mL catalyst suspension in ethanol (presonicated for 1 h) and 20 μL of Nafion solution (Sigma-Aldrich, ∼5 wt % in a mixture of alcohols and water) (0.1% v/v Nafion) were mixed together. After sonicating the suspension for 20 min, 50 μL of the catalyst mixture were drop-casted on both sides of a 0.5 × 0.5 cm Toray carbon paper (Alfa Aesar, TGP-H-60) to provide a geometric catalyst mass loading of 0.1 mg cm^–2^ (*A* = 0.5 cm^2^). The electrode was dried under ambient condition for least 3 h before the electrochemical measurements. For the RDE experiments, 0.49 mL catalyst suspension in ethanol (presonicated for 1 h) was mixed together with 0.49 mL ultrapure water and 20 μL of Nafion solution (Sigma-Aldrich, ∼5 wt % in a mixture of alcohols and water) (0.1% v/v Nafion). After sonicating the suspension for 20 min, 20 μL of the catalyst mixture were drop-casted on a 0.196 cm^2^ RDE. The electrode was dried under ambient condition for at least 1 h before electrochemical measurements.

### Spectrophotometric Product Analysis and Detection

UV–vis spectroscopy (Agilent Cary 60) was used to detect and quantify ammonia and nitrite as possible products of NO_3_RR and NO_2_RR, following the experimental procedures recently reported by previous literature.^49,67^ The original samples were properly diluted in order to match the suitable detection range for spectrophotometric analysis of each analyte and the sample absorbance was measured in the range of 400–800 nm.

#### Determination of Ammonia

The indophenol-blue method was used for the spectrophotometric determination of ammonia. Briefly, 500 μL of phenol nitroprusside solution (P6994, Sigma-Aldrich) and 500 μL of alkaline hypochlorite solution (A1727, Sigma-Aldrich) were added to 2 mL of a diluted electrolytic sample. The solution was incubated for 30 min at room temperature in the dark before recording the UV–vis spectrum. Standard solutions with known NH_4_Cl (99.998%, Sigma-Aldrich) concentration were used to construct a calibration curve. For electrocatalytic NO_3_RR experiments, the Faradaic efficiency [FE(NH_3_)] and the yield rate (*r*_NH_3__, based on mmol h^–1^ cm^–2^ or μg_product_ h^–1^ mg_catalyst_^–1^) for ammonia formation were calculated according to eq 2, eqs 3, and 4),^68−70^ respectively
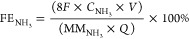
2
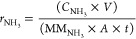
3
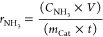
4where *C*_NH_3__ is the mass concentration of NH_3_(aq) (mg/L), *V* is the volume of electrolyte in the cathode compartment (18 mL), *F* is the Faradaic constant (96,485 C mol^–1^), MM_NH_3__ is the molar mass of NH_3_, *Q* is the charge passed during electrolysis, *m*_Cat_ is the total mass on the electrode (for 0.5 cm^2^ electrode is 0.05 mg), *A* is the geometric surface of the electrode (0.5 cm^2^), and *t* is the electrolysis time.

#### Determination of Nitrite

For nitrite (NO_2_^–^) quantification, 3 mL of the diluted sample were added to a glass vial containing 35 mg of white powder from a commercial nitrite test kit (photometric 0.002–1.00 mg L^–1^ NO_2_–N, 0.007–3.28 mg L^–1^ NO_2_^–^, Spectroquant, Merck). The mixture was then incubated for 10 min at room temperature in the dark before UV–vis analysis. Analogously to ammonia, a calibration curve was constructed by using standard NaNO_2_ (99%, for analysis, Sigma-Aldrich) concentrations. For electrocatalytic NO_3_RR experiments, the Faradaic efficiency [FE(NO_2_^–^)] and the yield rate (*r*_NO_2__^–^, based on mmol h^–1^ cm^–2^ or μg_product_ h^–1^ mg_catalyst_^–1^) for nitrite formation were calculated according to (eq 5) and (eqs 6 and 7), respectively
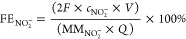
5
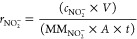
6
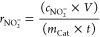
7where *c*_NO_2__^–^is the mass concentration of NO_2_^–^(aq) (mg/L), MM_NO_2__^–^ is the molar mass of NO_2_^–^, the other parameters are similar like those for calculating ammonia FE and yield rate.

### Nuclear Magnetic Resonance Spectroscopy

^1^H NMR spectra were provided by using a 600-MHz instrument (JEOL) available at the Freie Universität of Berlin. In a typical measurement for ammonia quantification, 10 mL of the used electrolyte solution were added to a clean glass vial containing 5.0 mg of maleic acid used as an internal standard (ReagentPlus, ≥99% (HPLC), Sigma-Aldrich), followed by the addition of 2.5 mL H_2_SO_4_ 4 M to acidify the mixture. After ensuring complete miscibility, the nuclear magnetic resonance tube was filled by mixing 500 μL of the mixture and 50 μL of deuterium oxide (D_2_O, 99.9 atom %D, Sigma-Aldrich). The concentration of ^14^NH_4_^+^/^15^NH_4_^+^ in the samples was derived from the integral ratio of the triplet/doublet ammonium signal and the singlet of the maleic acid standard. Standard solutions with known ^14^NH_4_Cl (99.998%, Sigma-Aldrich) and ^15^NH_4_Cl (Sigma-Aldrich, ≥98 atom % ^15^N, ≥99%) concentration were used to construct calibration curves for ^14^NH_4_^+^ and ^15^NH_4_^+^ determination, respectively.

## Supporting Information Available

Additional TEM images, quasi in situ XPS, in situ Raman, operando XAS results, and electrochemical data are supplied as Supporting Information (PDF)
