# Supplementary material for: Electrocatalytic Nitrate and Nitrite Reduction toward Ammonia Using Cu2O Nanocubes: Active Species and Reaction Mechanisms
Source: J Am Chem Soc. 2024 Apr 1;146(14):9665–78. doi: 10.1021/jacs.3c13288 (PMC11009949; doi:10.1021/jacs.3c13288)
Supplement: Supplementary file 1 — ja3c13288_si_001.pdf [file ja3c13288_si_001.pdf]

Supporting Information

Electrocatalytic nitrate and nitrite reduction towards ammonia using Cu_2O nanocubes: active species and reaction mechanisms

Lichen Bai[a,$], Federico Franco[a,$,†], Janis Timoshenko[a], Clara Rettenmaier[a], Fabian Scholten[a], Hyo Sang Jeon[a,‡], Aram Yoon[a], Martina Rüscher[a], Antonia Herzog[a], Felix T. Haase[a], Stefanie Kühl[a], See Wee Chee[a], Arno Bergmann[a], Beatriz Roldan Cuenya[a]*

a. Department of Interface Science, Fritz-Haber-Institute of Max-Planck-Society, Faradayweg 4-6, 14195 Berlin, Germany.

$ L. Bai and F. Franco contributed equally to this work.

† Current address: Department of Chemical and Pharmaceutical Sciences, University of Trieste, 34127 Trieste, Italy.

‡ Current address: Technological Convergence Center, Korea Institute of Science and Technology (KIST), 02792 Seoul, Republic of Korea.

* Corresponding author email: roldan@fhi-berlin.mpg.de

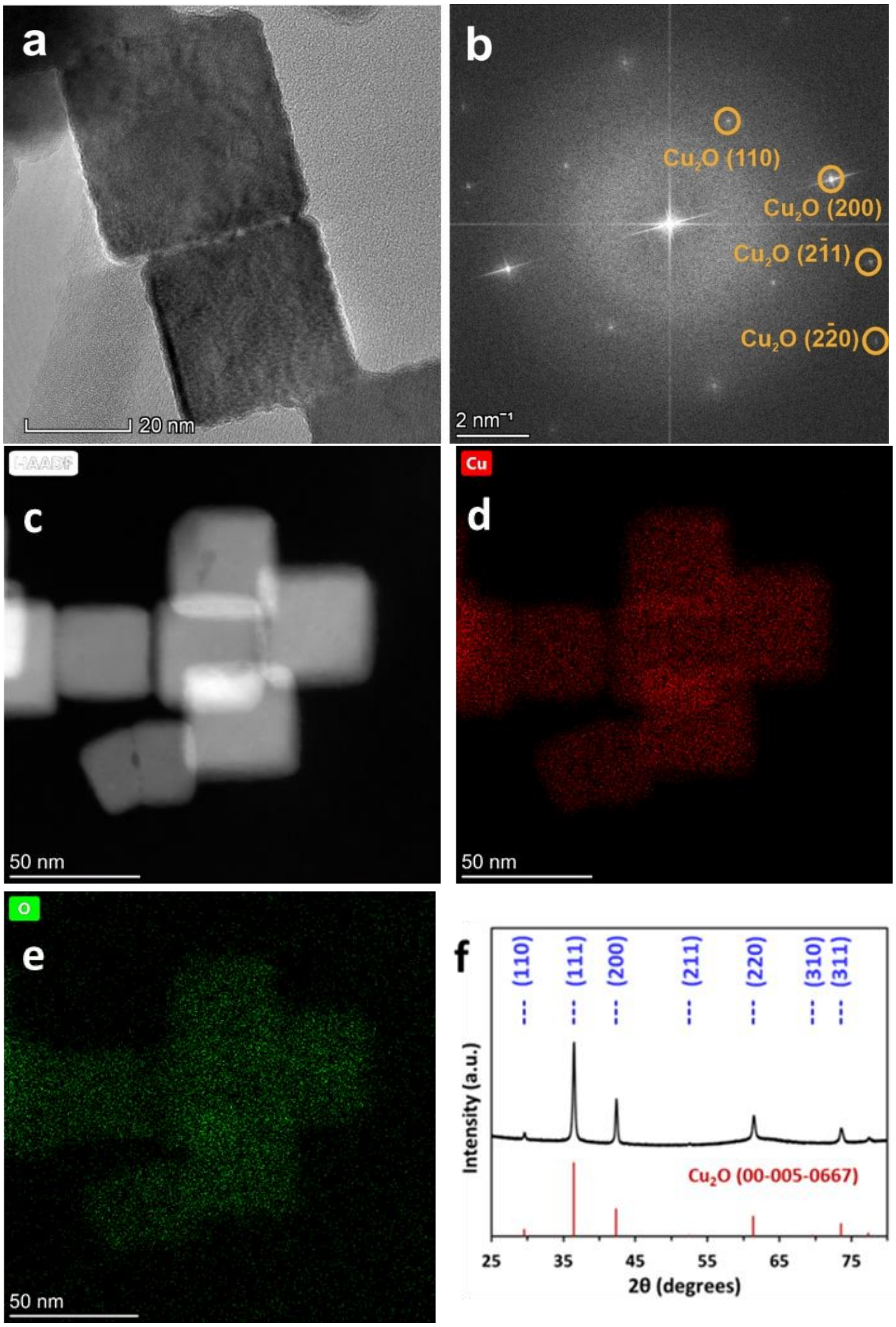

Figure S1. Characterization of Cu_2O nanocubes (NCs). (a) High resolution transmission electron microscopy (HRTEM) and (b) the corresponding fast Fourier transfer (FFT) pattern, (c) high angular annular dark field scanning transmission electron microscopy (HAADF-STEM) images of the as prepared Cu_2O NCs; (d,e) corresponding energy dispersive X-ray (EDX) mapping of (c) with Cu (red) in (d) and O (green) in (e). (f) Powder X-ray diffraction (PXRD) pattern reveals that the crystallographic structure agrees with that of the bulk Cu_2O phase (JCPDS No. 00-005-0667).

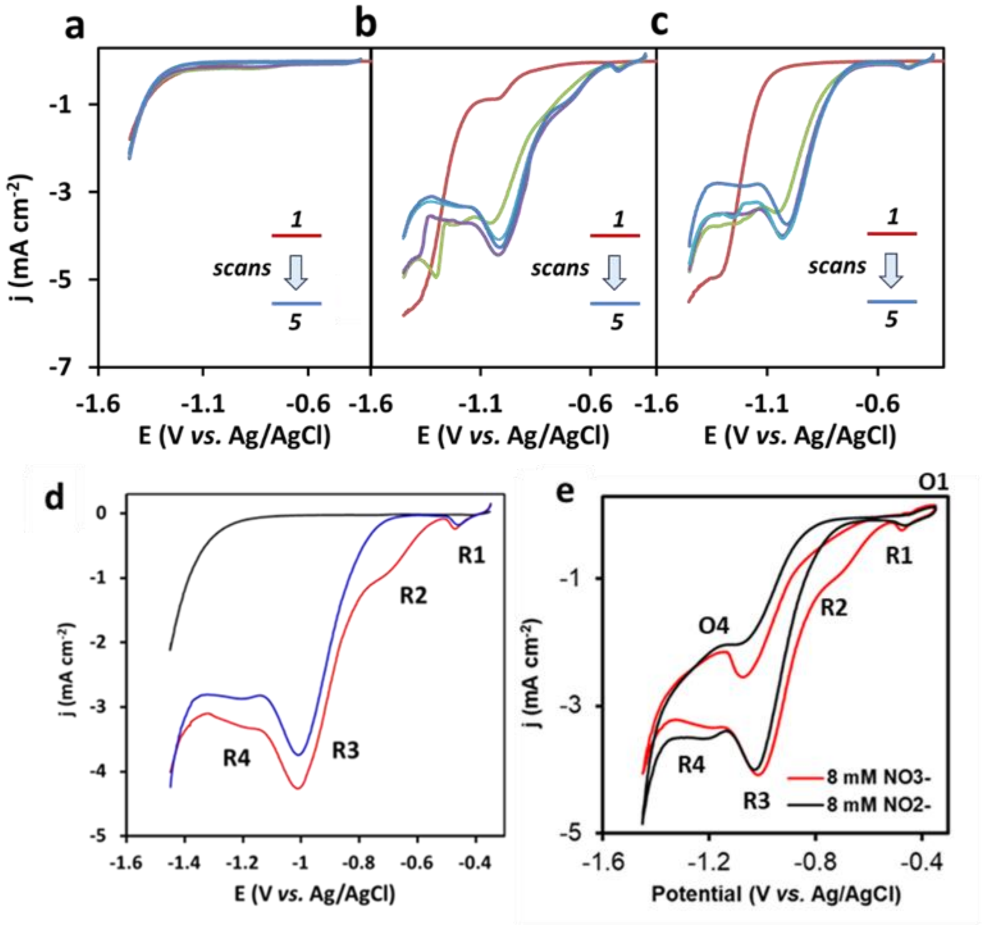

Figure S2. Voltammetric behavior of Cu_2O NCs in pH-neutral electrolyte. (a-c) Five consecutive LSVs of Cu_2O NCs in 0.1 M Na_2SO_4 (a), 0.1 M Na_2SO_4 + 8 mM NO_3^- (b) and 0.1 M Na_2SO_4 + 8 mM NO_2^- (c). The LSVs are collected under an inert Ar atmosphere. (d) Comparison of LSVs recorded in 0.1 M Na_2SO_4 electrolyte without added NO_3^-/NO_2^- sources (black) and in the presence of 8 mM $NaNO_3$ (red) or 8 mM $NaNO_2$ (blue), respectively. (e) CVs in 0.1 M Na_2SO_4 + 8 mM NO_3^- (red) or NO_2^- (black). The data was collected after five CVs between -0.35 and -1.45 $V_{Ag/AgCl}$. Scan rate = 10 mV/s.

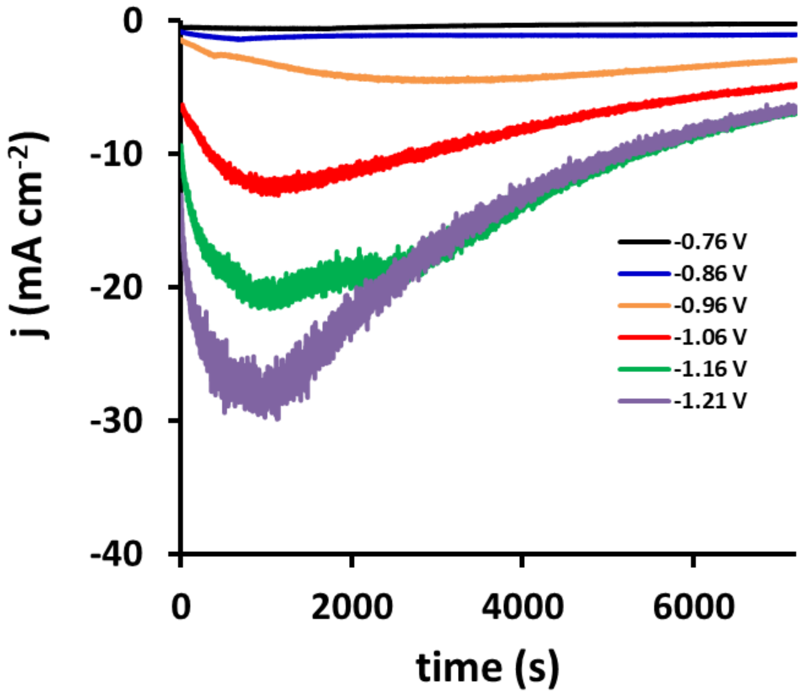

Figure S3. Representative chronoamperometric curves (j *vs.* time, 2h electrolysis) on Cu_2O NCs in 0.1 M Na_2SO_4 + 8 mM $NaNO_3$ at different applied potentials (vs. Ag/AgCl).

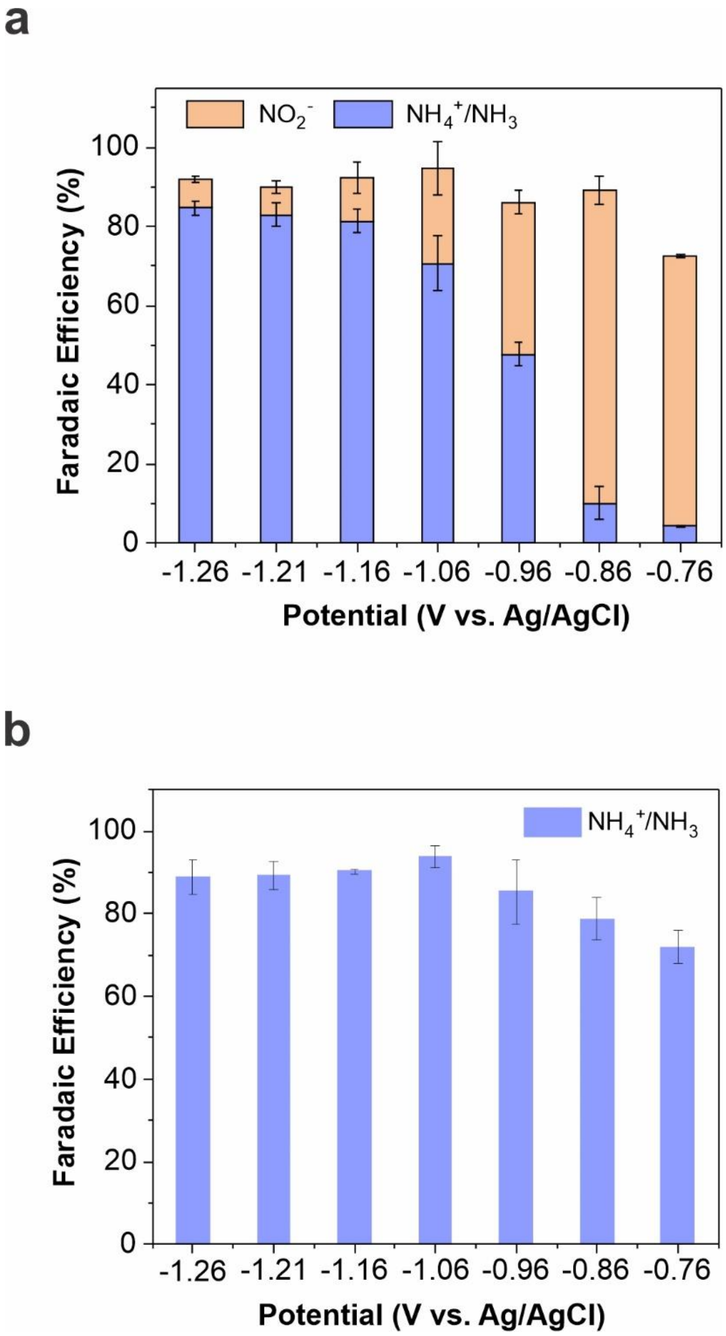

Figure S4. Faradaic Efficiency (FE) of Cu_2O NCs for nitrate and nitrite reduction in pH-neutral electrolyte. (a) Potential-dependent FEs for NH_3/NO_2^- production during chronoamperometric NO_3RR tests (2h) in 0.1 M Na_2SO_4 + 8 mM $NaNO_3$. (b) Potential-dependent FEs for NH_3 production during chronoamperometric NO_2RR tests (2h) in 0.1 M Na_2SO_4 + 8 mM $NaNO_2$.

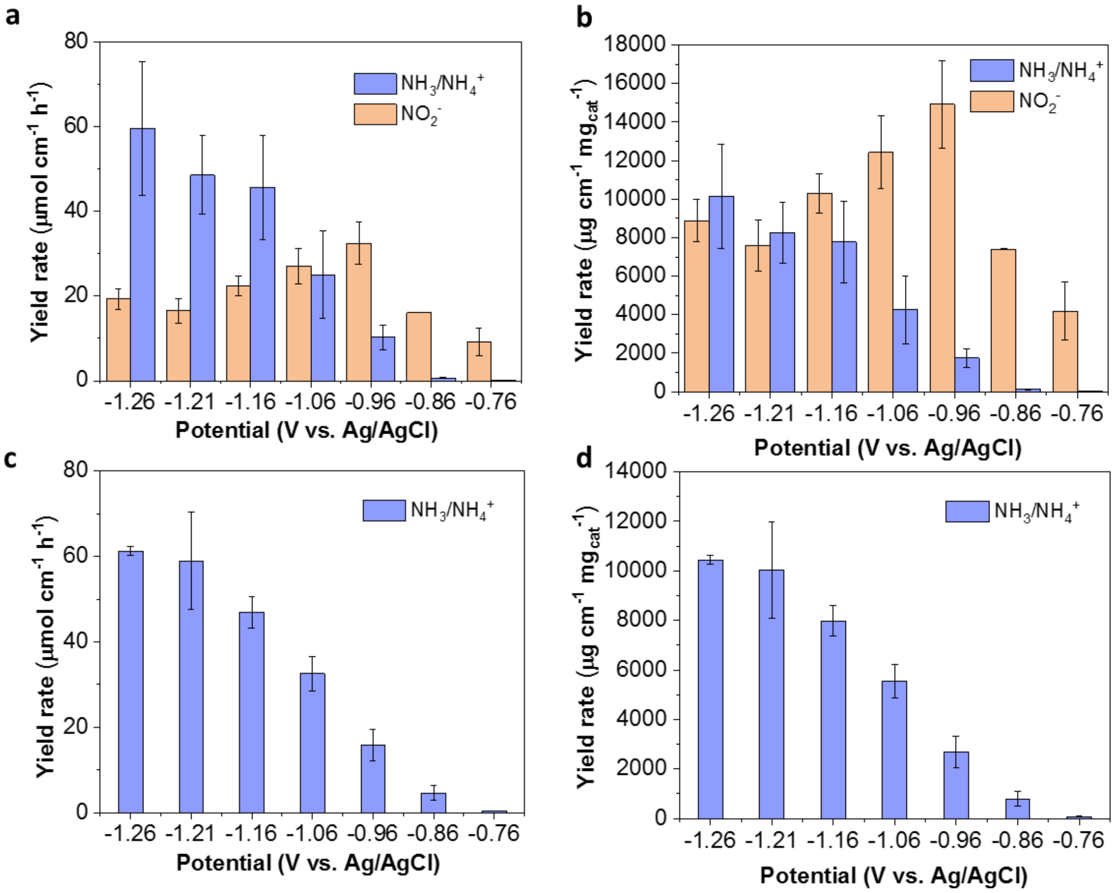

Figure S5. Product Yield rate of Cu_2O NCs for nitrate and nitrite reduction in pH-neutral electrolyte. (a-b) Potential dependent yield rate of different products (NH_3/NO_2^-) for a 2h electrolysis of Cu_2O NCs in 0.1 M Na_2SO_4 + 8 mM $NaNO_3$. (c-d) Potential dependent yield rate of ammonia for a 2h electrolysis of Cu_2O NCs in 0.1 M Na_2SO_4 + 8 mM $NaNO_2$.

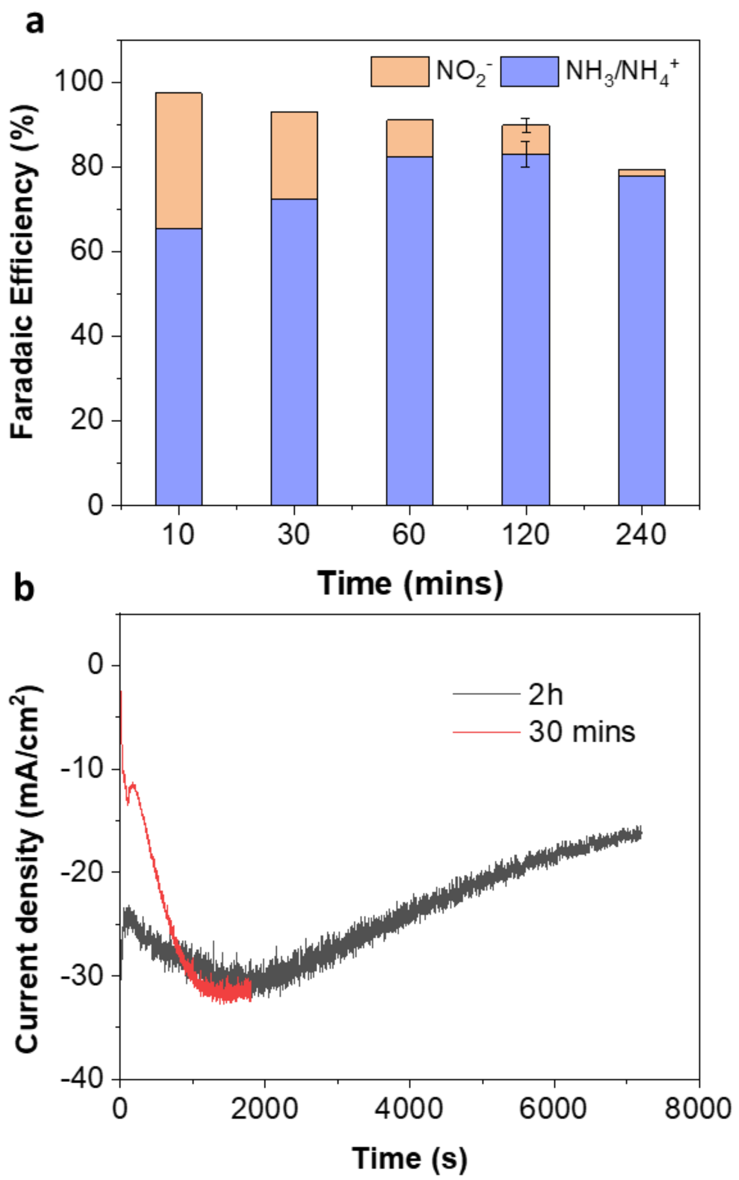

Figure S6. Time-dependent performance of Cu_2O NCs for nitrate reduction. (a) Faradaic efficiency of Cu_2O NCs measured after electrolysis with different duration time. (b) Representative chronoamperometric curves of Cu_2O NCs for electrolysis times of 30 min and 2 h. Applied potential: -1.21 V vs. Ag/AgCl. Electrolyte: 0.1 M Na_2SO_4 + 8 mM $NaNO_3$.

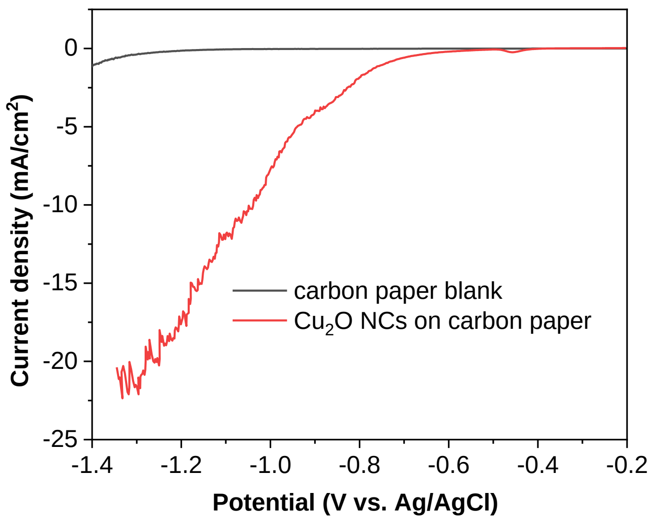

Figure S7. Linear scan voltammograms (LSV) of Cu_2O NCs (red) and blank carbon paper (black) in 0.1 M Na_2SO_4 + 8 mM $NaNO_3$. The LSVs were recorded under stirring.

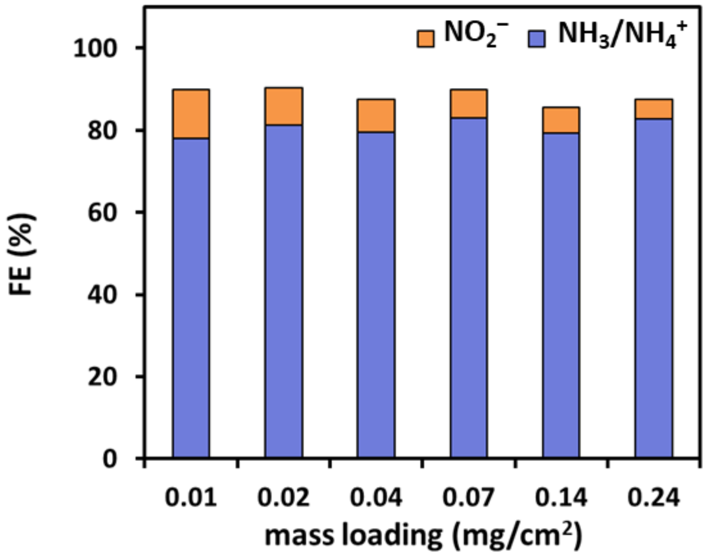

Figure S8. Faradaic efficiency for NO_2^- and NH_3 production obtained after 2h electrolysis on Cu_2O NCs at different catalyst loadings in 0.1 M Na_2SO_4 + 8 mM $NaNO_3$ at -1.21 $V_{Ag/AgCl}$.

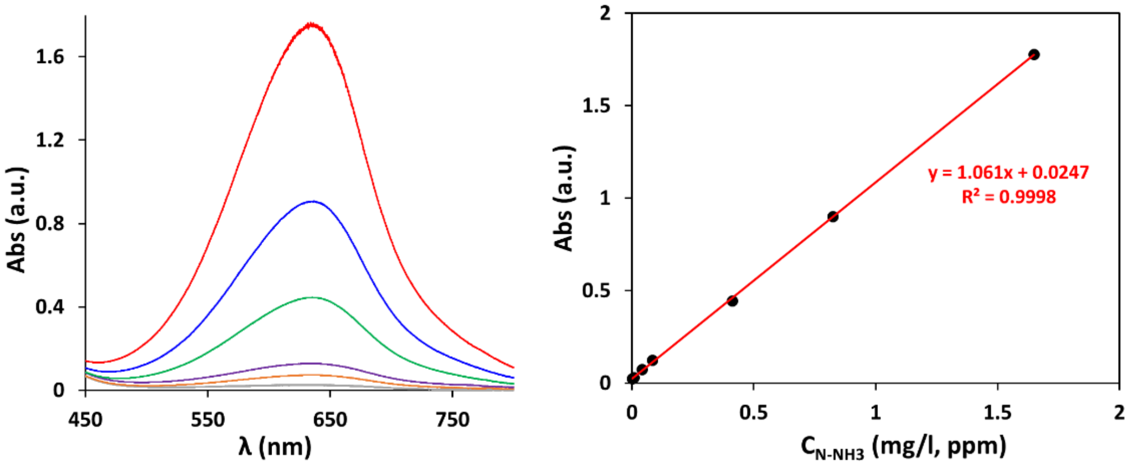

Figure S9. Calibration curve for NH_3 quantification by the spectrophotometric blue-indophenol method.

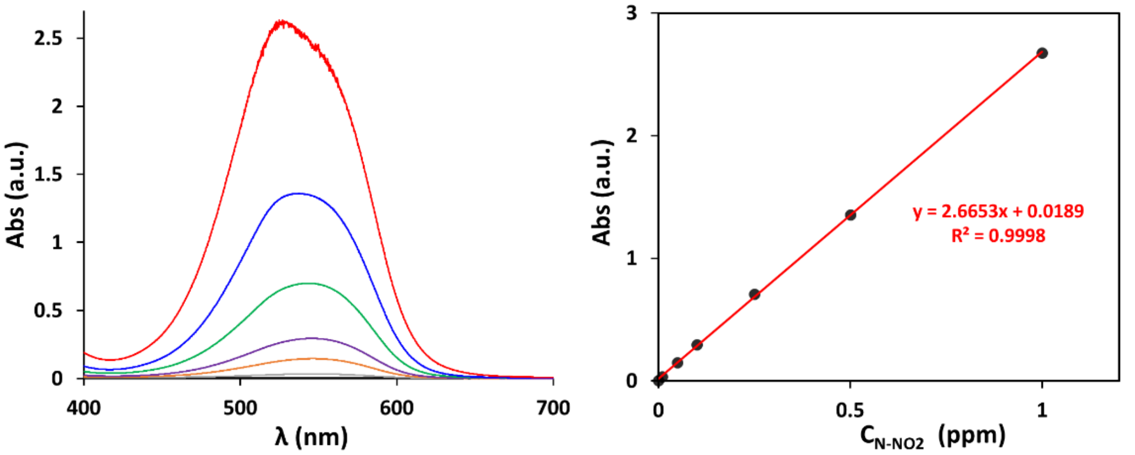

Figure S10. Calibration curve for NO_2^- quantification by the spectrophotometric method.

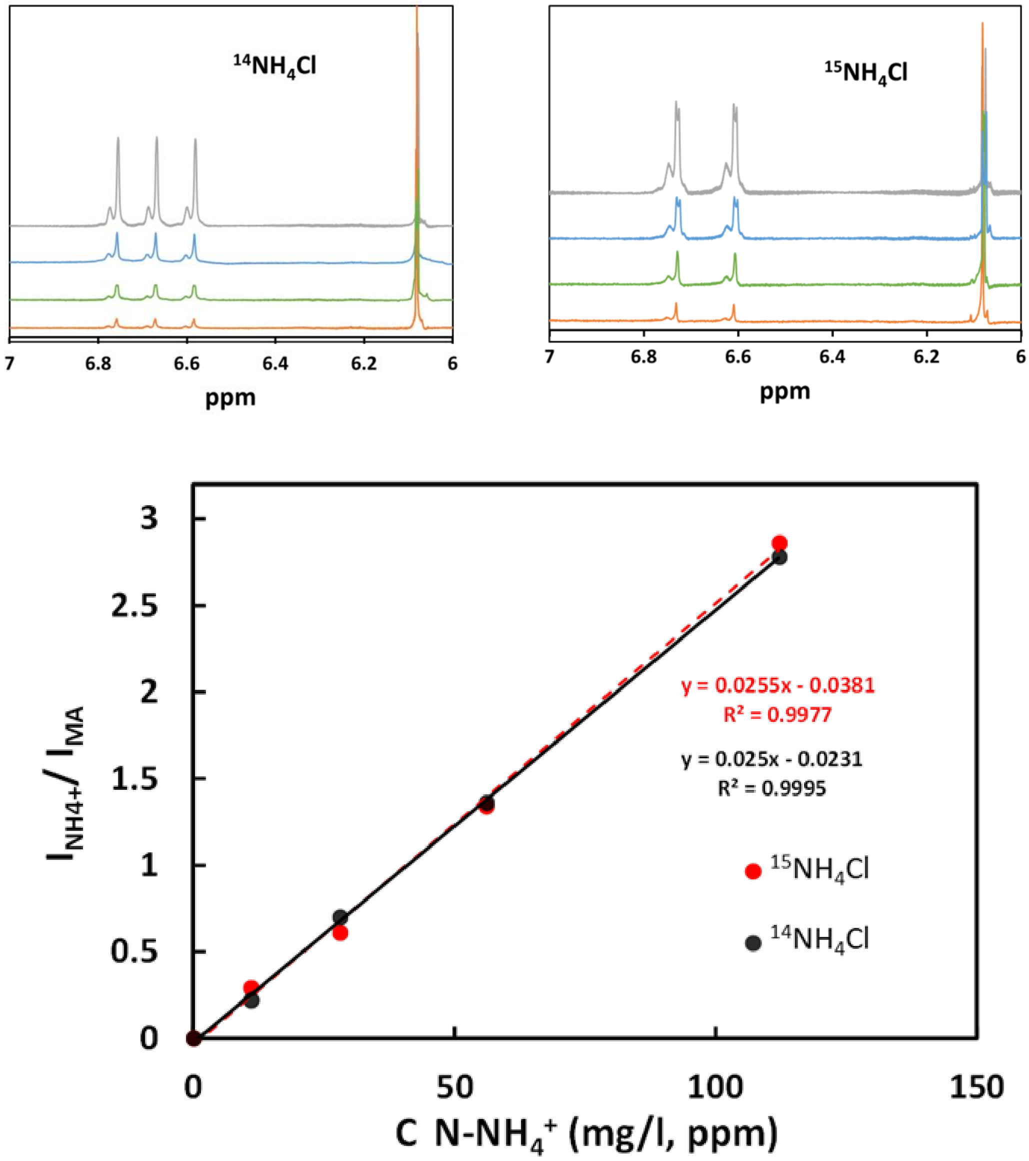

Figure S11. Calibration curves and corresponding ^{1}H-NMR spectra of standard $^{14}NH_4Cl$ and $^{15}NH_4Cl$ solutions.

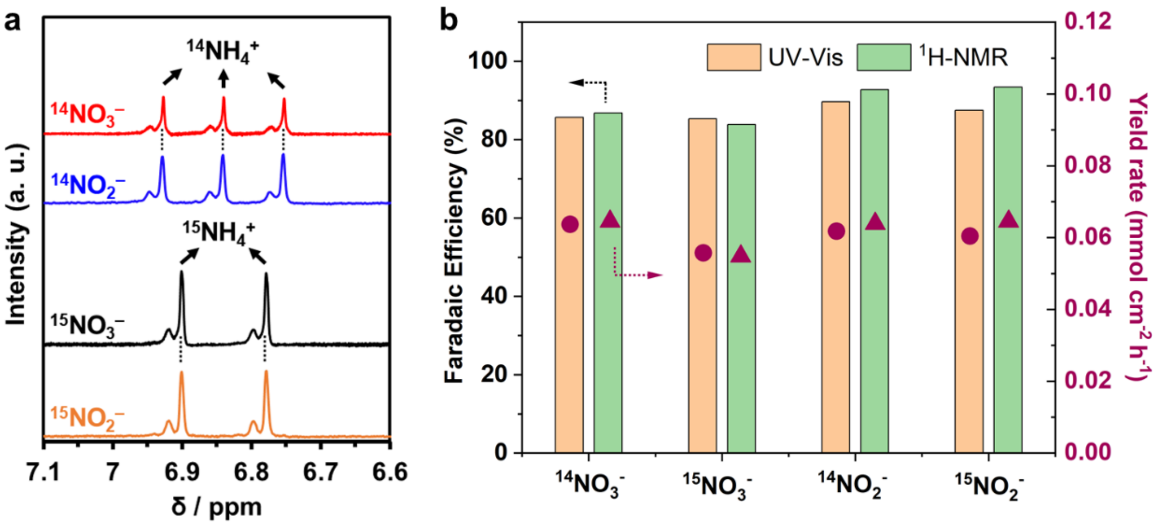

Figure S12. Ammonia quantification using ^{1}H-NMR. (a) ^{1}H-NMR spectra of the electrolyte after 2h electrolysis at –1.21 $V_{Ag/AgCl}$ in the presence of 8 mM $Na^{14}NO_3$ (red), $Na^{14}NO_2$ (blue), $Na^{15}NO_3$ (black) or $Na^{15}NO_2$ (orange). (b) Comparison between FEs and yield rates for NH_3 production obtained by UV-Vis indophenol method and ^{1}H-NMR analysis. Supporting electrolyte: 0.1 M Na_2SO_4.

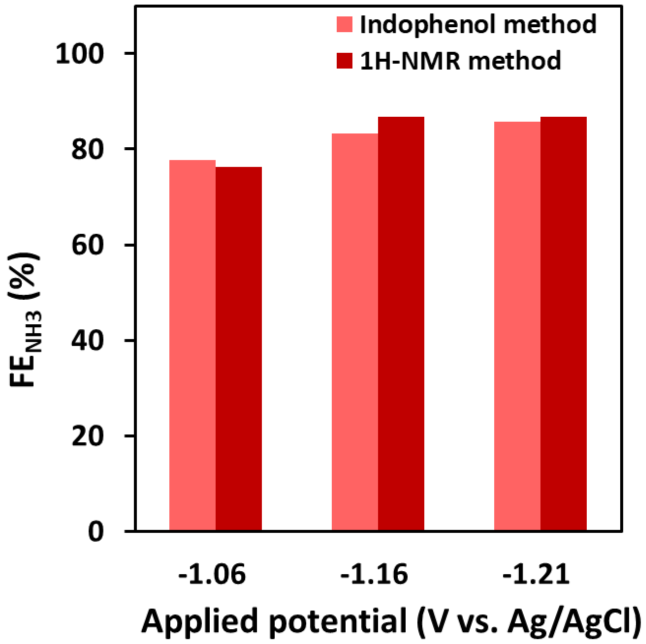

Figure S13. Comparison of FE_{NH3} obtained by the colorimetric and ^{1}H-NMR methods after 2h electrolysis of Cu_2O NCs at different applied potentials. Electrolyte: 0.1 M Na_2SO_4 + 8 mM $Na^{14}NO_3$.

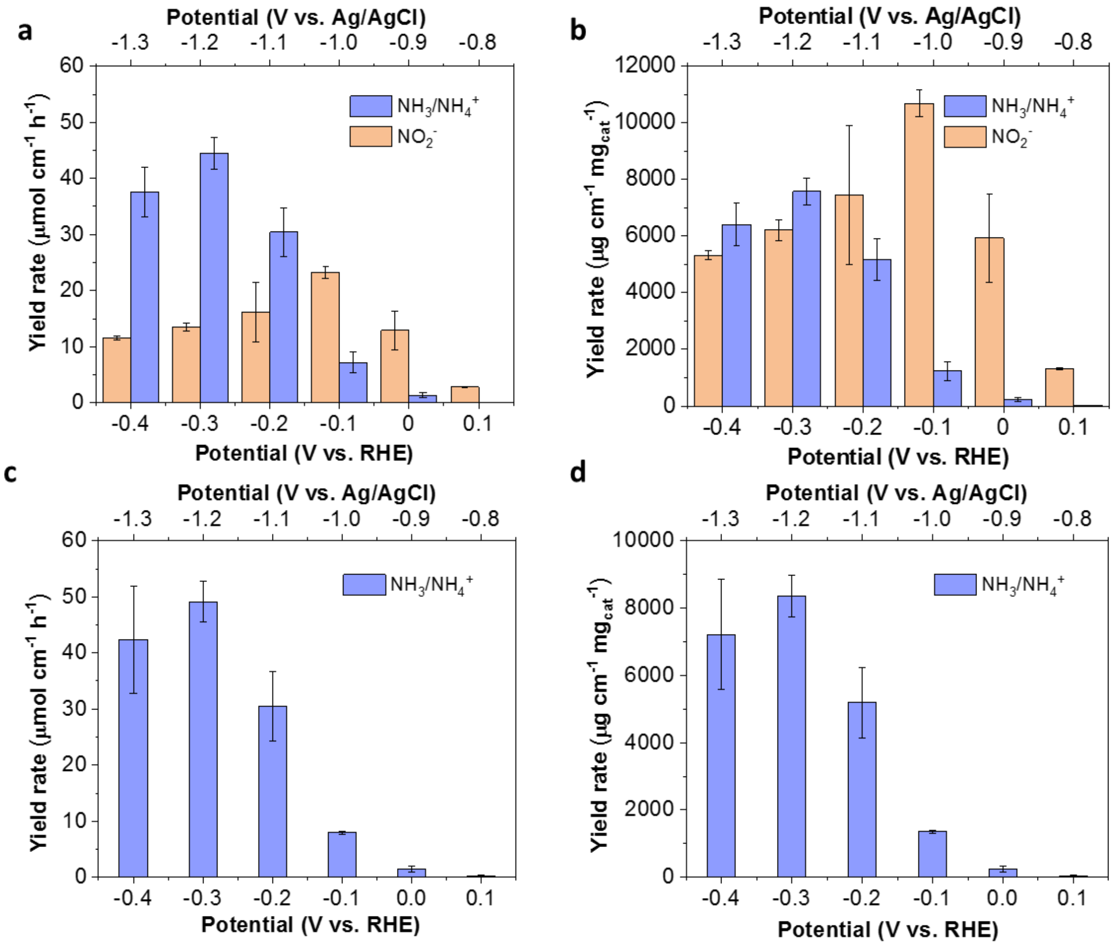

Figure S14. NO_3RR and NO_2RR performance (product yield rate) of Cu_2O NCs in pH 12.0 electrolyte. Potential-dependent yield rate of different products (NH_3/NO_2^-) for a 2h electrolysis of the Cu_2O NCs electrode in (a-b) 0.1 M Na_2SO_4 + 8 mM $NaNO_3$ at pH 12; (c-d) 0.1 M Na_2SO_4 + 8 mM $NaNO_2$ at pH 12.

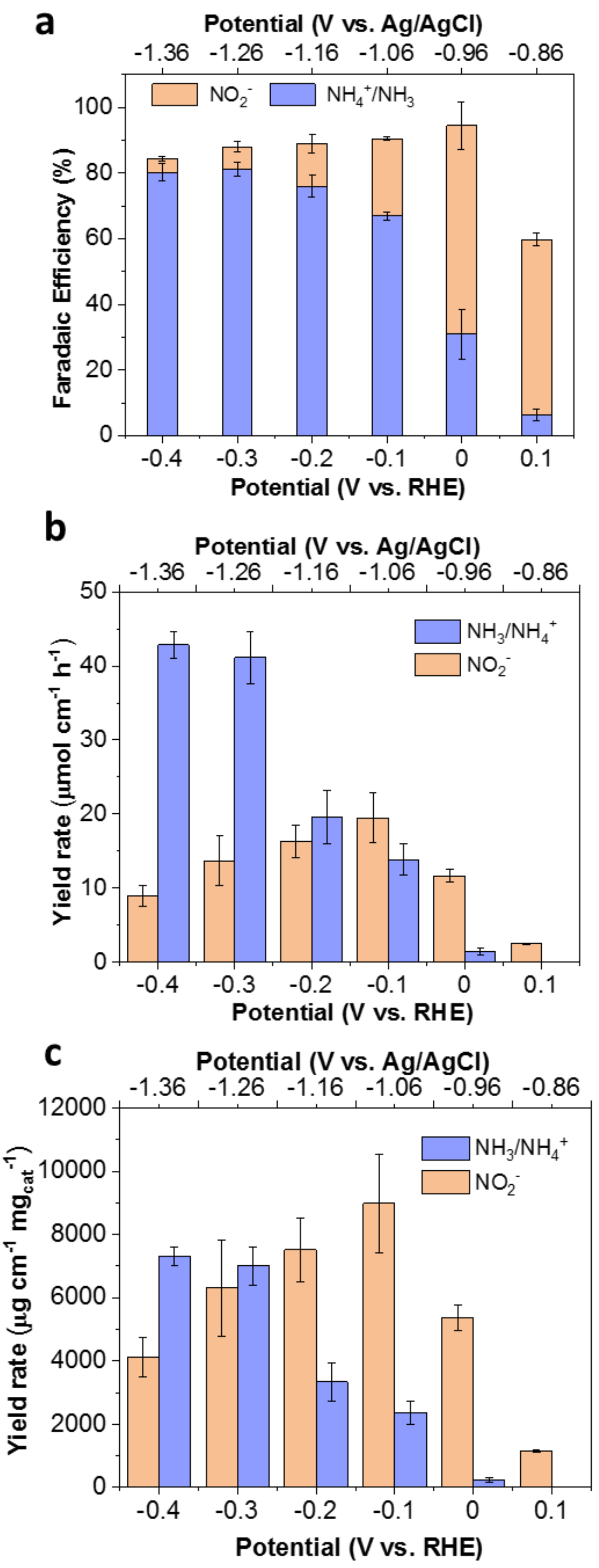

Figure S15. NO_3RR performance of Cu_2O NCs in pH 12.9 electrolyte. (a) Potential-dependent FEs of different products (NH_3/NO_2^-) production for a 2h electrolysis of Cu_2O NCs electrode in 0.1 M NaOH + 8 mM $NaNO_3$ (pH 12.9). (b-c) Potential-dependent yield rate of different products (NH_3/NO_2^-) production for a 2h electrolysis of Cu_2O NCs electrode in 0.1 M NaOH + 8 mM $NaNO_3$ (pH 12.9).

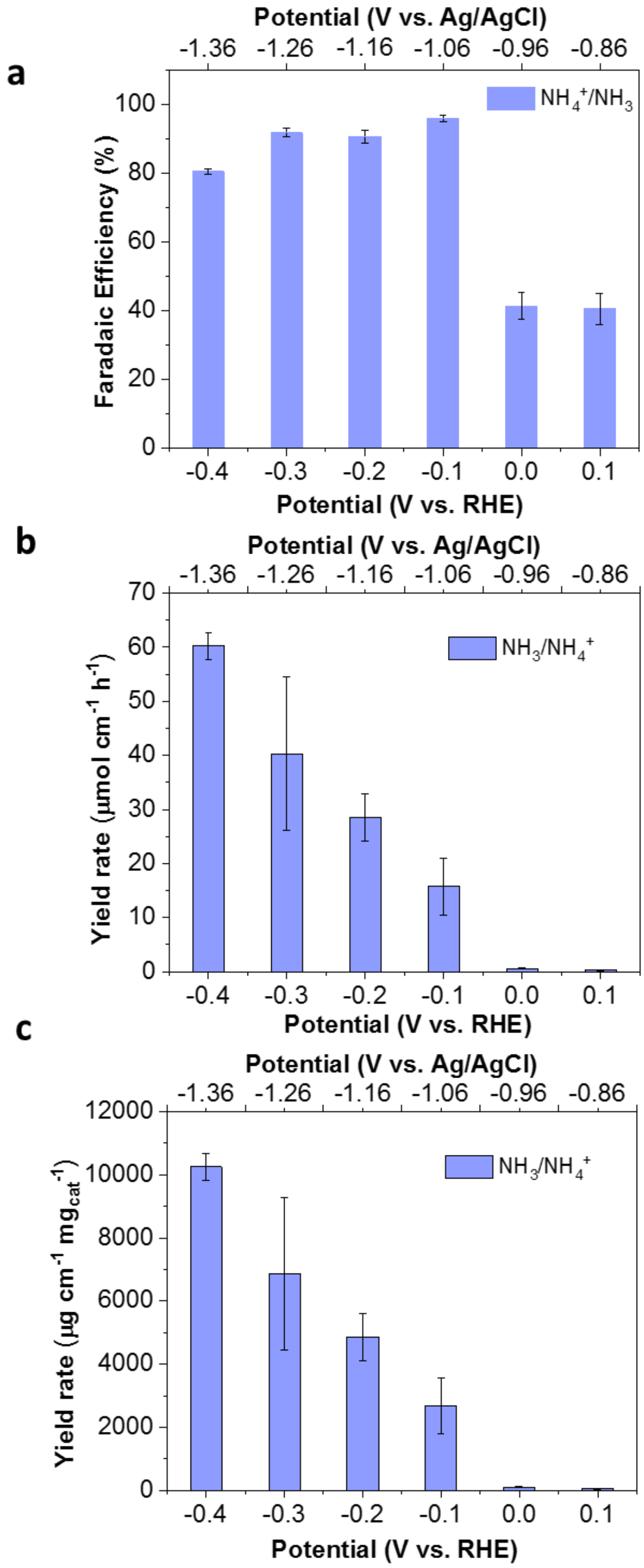

Figure S16. NO_2RR performance of Cu_2O NCs in pH 12.9 electrolyte. (a) Potential-dependent FEs of ammonia production for a 2h electrolysis of Cu_2O NCs electrode in 0.1 M NaOH + 8 mM $NaNO_2$ (pH 12.9). (b-c) Potential-dependent yield rate of ammonia production for a 2h electrolysis of Cu_2O NCs electrode in 0.1 M NaOH + 8 mM $NaNO_2$ (pH 12.9).

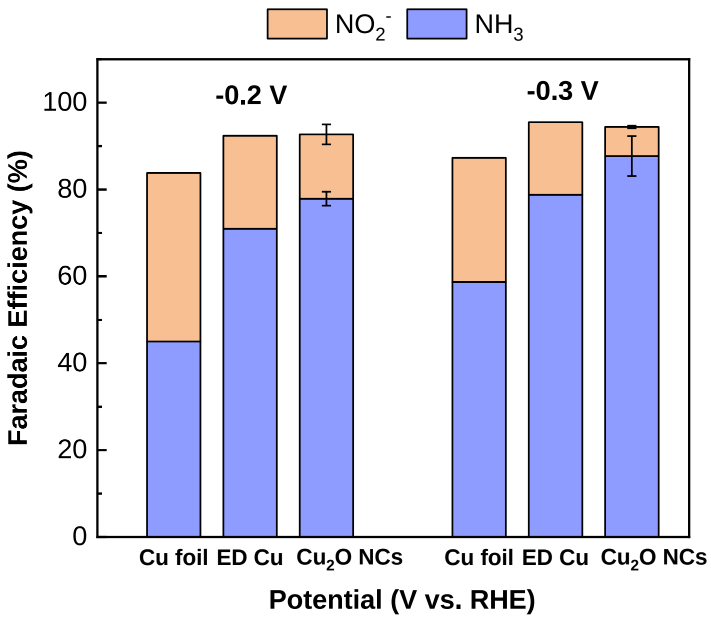

Figure S17. **Comparison of potential-dependent Faradaic Efficiency for an electro-polished Cu foil, electrodeposited Cu (ED Cu) and Cu_2O NCs.** Electrolyte: 0.1 M Na_2SO_4, with 8 mM $NaNO_3$, pH 12. The electrolysis time is 1 h for electro-polished Cu foil (synthesized according to Ref. [1]) and electrodeposited (ED) Cu (synthesized according to Ref. [2]), while for Cu_2O NCs the electrolysis time is 2 h.

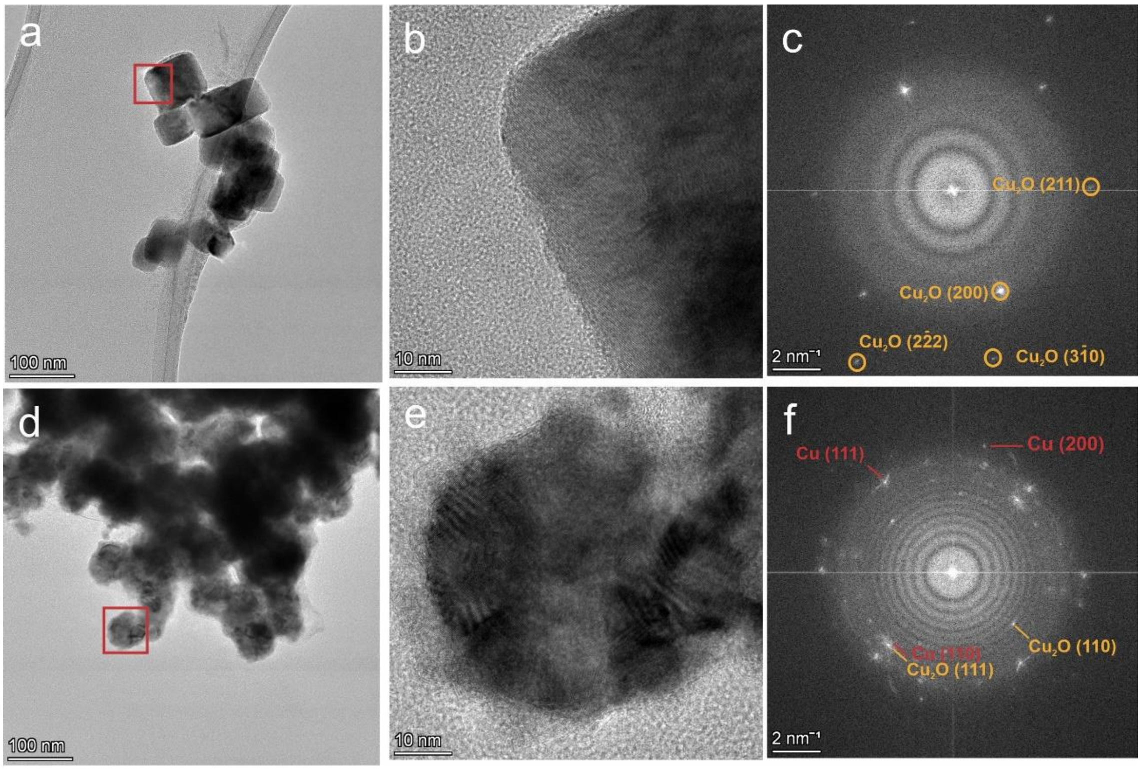

Figure S18. TEM images of Cu_2O NCs after NO_3RR. (a-c) After 2h electrolysis at 0.1 V_{RHE}. (d-f) After 2h electrolysis at -0.3 V_{RHE}. (a, d) are TEM images and (b, e) are HRTEM images of Cu_2O NCs. (c) is the FFT patterns of (b) and (f) is the FFT patterns of (e). The red rectangles in (a, d) mark the area for HRTEM. Electrolyte: 0.1 M Na_2SO_4 + 8 mM $NaNO_3$ at pH 12.

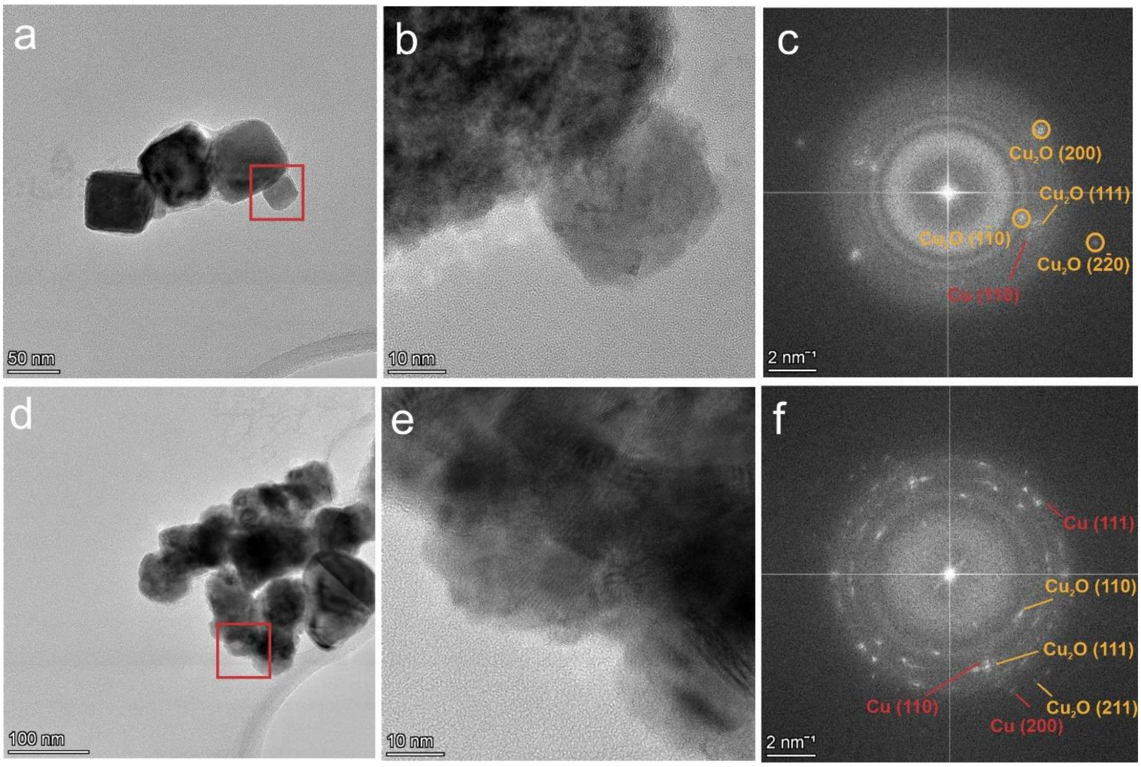

Figure S19. TEM images of Cu_2O NCs after NO_3RR. (a-c) After 2h electrolysis at 0.1 V_{RHE}. (d-f) After 2h electrolysis at -0.3 V_{RHE}. (a, d) are TEM images and (b, e) are HRTEM images of Cu_2O NCs. (c) is the FFT patterns of (b) and (f) is the FFT patterns of (e). The red rectangles in (a, d) mark the area for HRTEM. Comparing to **Figure S18**, the images are acquired in different regions of TEM grids. Electrolyte: 0.1 M Na_2SO_4 + 8 mM $NaNO_3$ at pH 12.

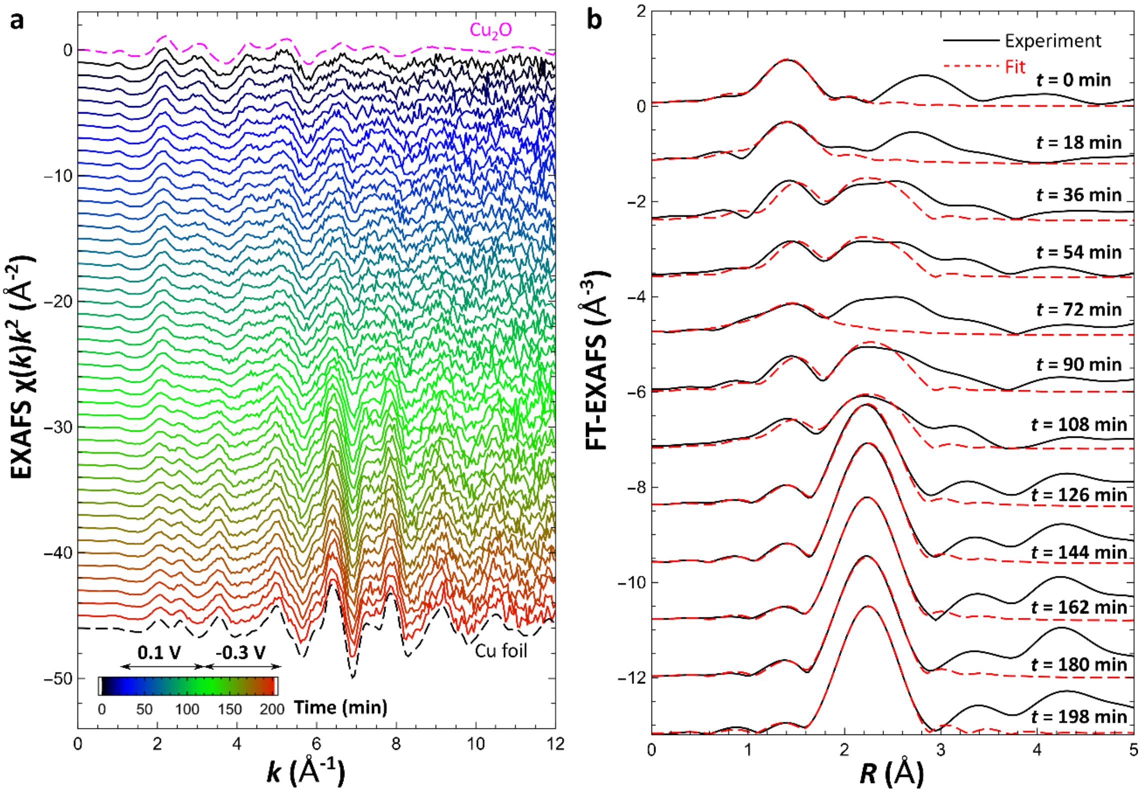

Figure S20. ***Operando*** **Cu K-edge EXAFS spectra and corresponding fitting results of Cu_2O NCs.** (a) Cu K-edge EXAFS spectra for Cu_2O NCs at OCP, 0.10 V_{RHE} and -0.30 V_{RHE}. Spectra are shifted vertically for clarity and are plotted as a function of the applied potential and also reaction time as indicated in the horizontal bar. Reference spectra for bulk Cu_2O and Cu foil are also shown. (b) Comparison of representative Fourier-transformed EXAFS spectra with the best fitting results. Electrolyte: pH 12, 0.1 M Na_2SO_4 + 8 mM $NaNO_3$.

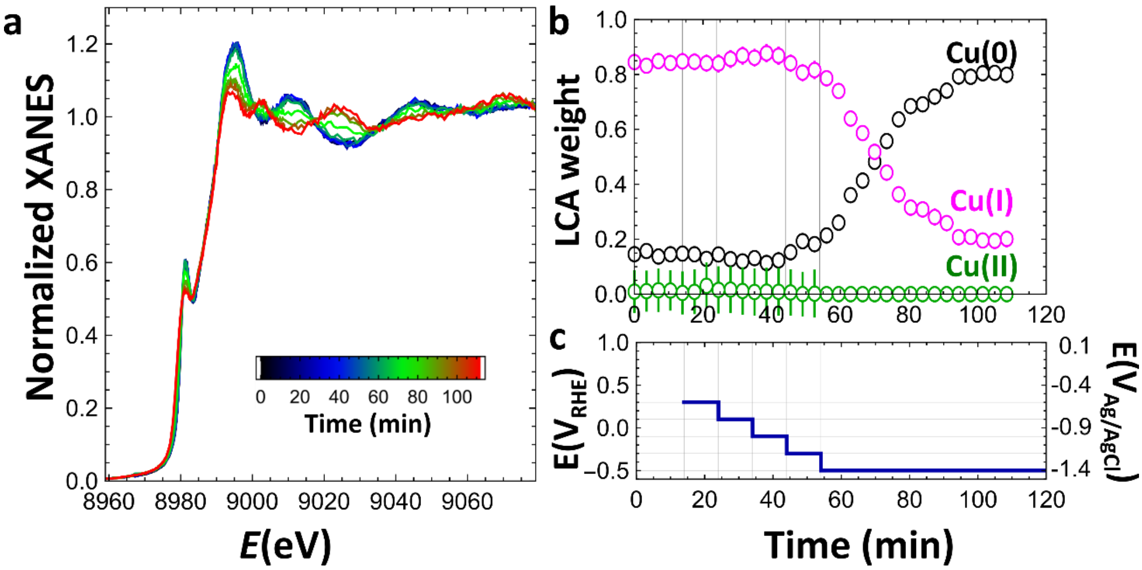

Figure S21. *Operando* XAS data of Cu_2O NCs during NO_3RR. (a) Normalized Cu K-edge XANES spectra collected at different applied potentials as indicated in panel (c). (b) Linear combination analysis (LCA) results for the XANES spectra, showing the potential-dependent variation of Cu oxidation state. Electrolyte: pH 12, 0.1 M Na_2SO_4 + 8 mM $NaNO_3$.

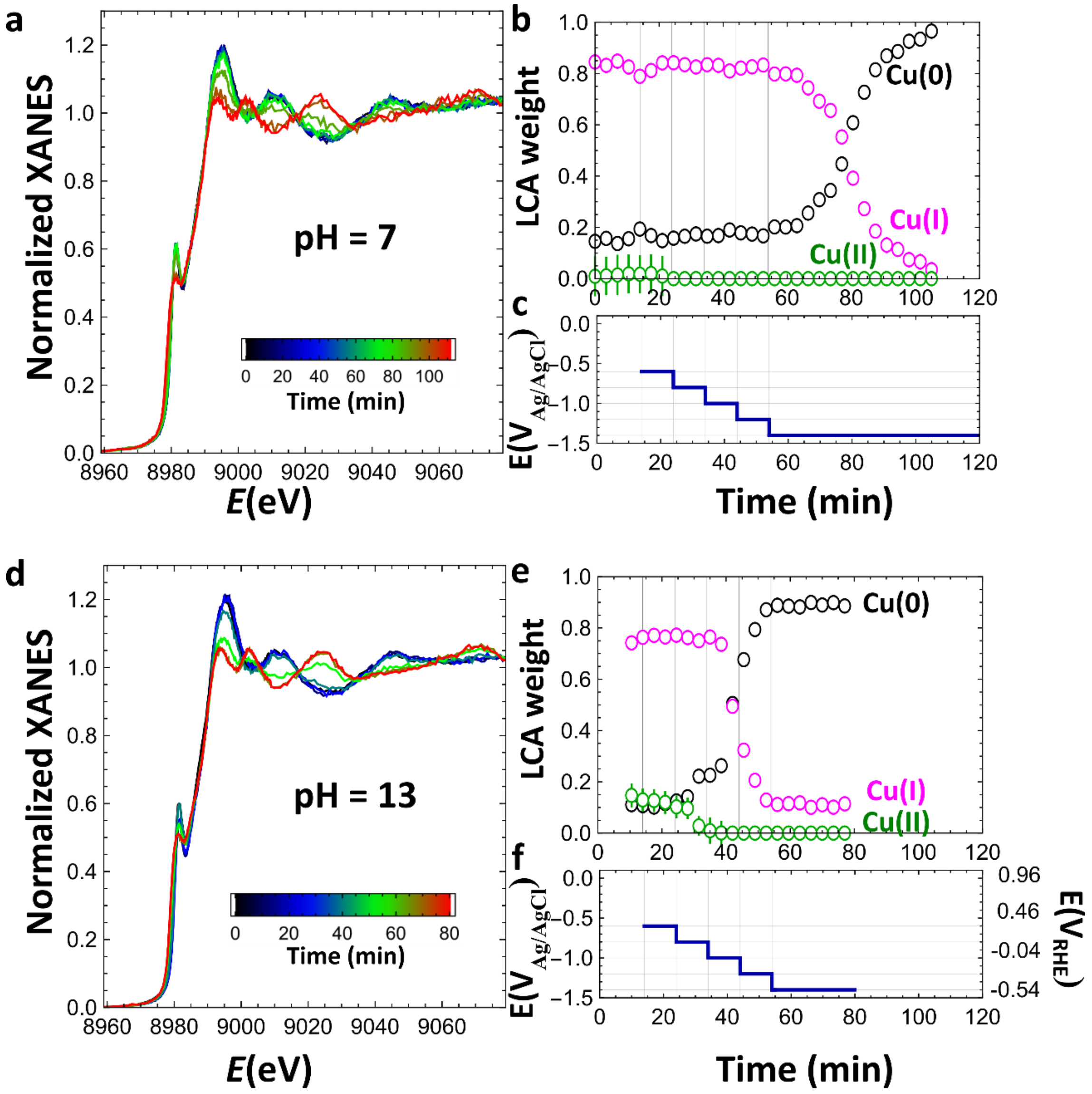

Figure S22. ***Operando*** **XAS experiments data of Cu_2O NCs during NO_3RR in electrolytes with pH 7 (a-c, electrolyte: 0.1 M Na_2SO_4 + 8 mM $NaNO_3$, the initial pH is 7 and it is gradually increased during reaction) and pH 12.9 (d-f, electrolyte 0.1 M NaOH + 8 mM $NaNO_3$).** (a,d) Normalized Cu K-edge XANES spectra collected at different applied potentials as indicated in panels (c) and (f), respectively. (b,e) Linear combination analysis (LCA) results for the XANES spectra, showing the potential-dependent variation of the Cu oxidation state. Note that the potential values in (c) are given with respect to the Ag/AgCl reference electrode.

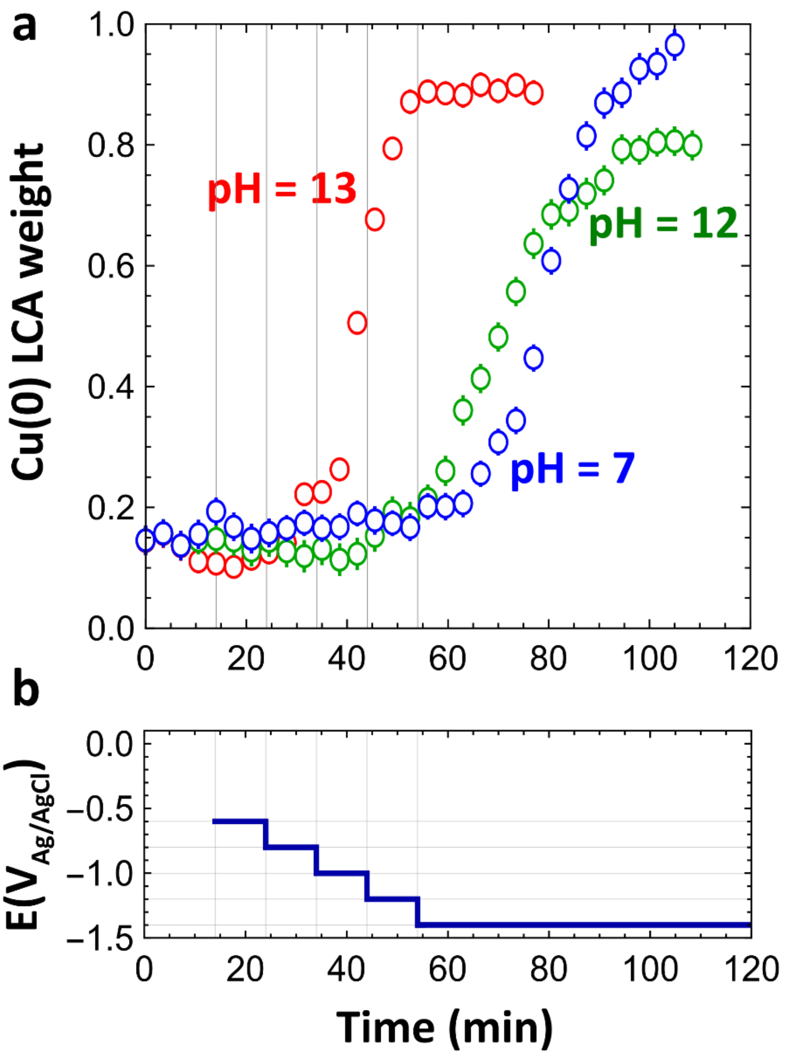

Figure S23. Results from the analysis of *operando* XAS data of Cu_2O NCs during NO_3RR in electrolytes with different pH. (a) Time and potential dependency of Cu(0) concentration under NO_3RR reaction conditions in electrolytes with different pH. The results were obtained from LCA fitting of operand Cu K-edge XANES spectra. (b) Time-dependency of the applied potential. Potential values are given with respect to Ag/AgCl reference electrode. Noted that the pH 7 is the initial pH, the pH value gradually increases during measurement (**Supplementary Note 1**).

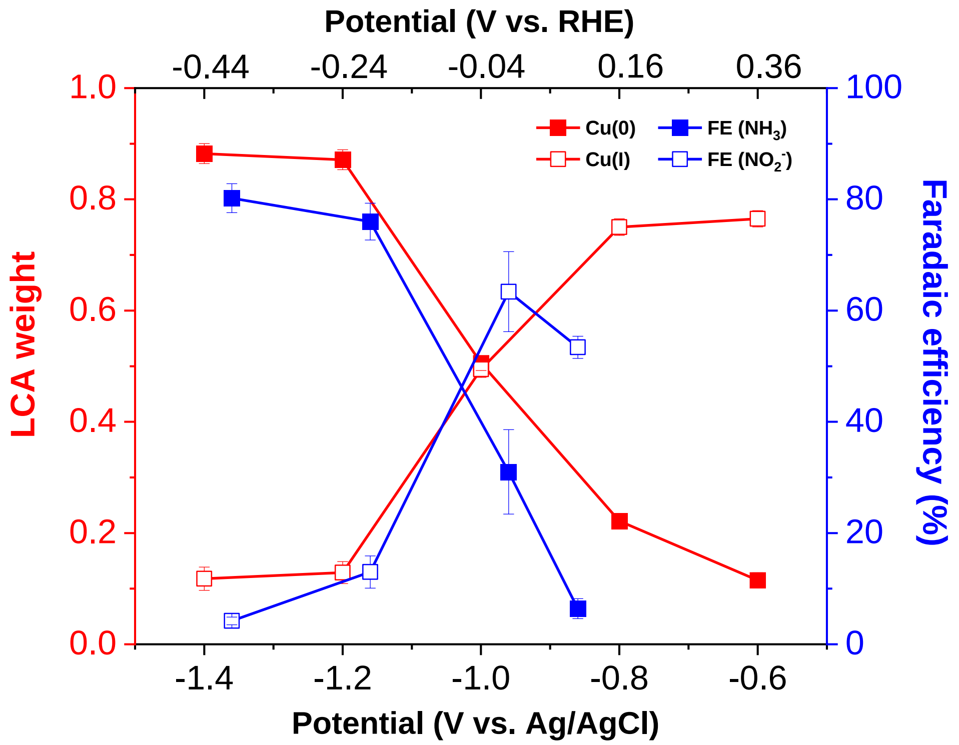

Figure S24. Potential-dependent Cu(I)/Cu(0) speciation and NO_2^-/NH_3 selectivity observed during NO_3RR at pH 12.9 (0.1 M NaOH + 8 mM $NaNO_3$). For LCA weight, the values are derived from **Figure S22e-f** at reaction time of 20, 30, 40, 50, and 60 min, respectively.

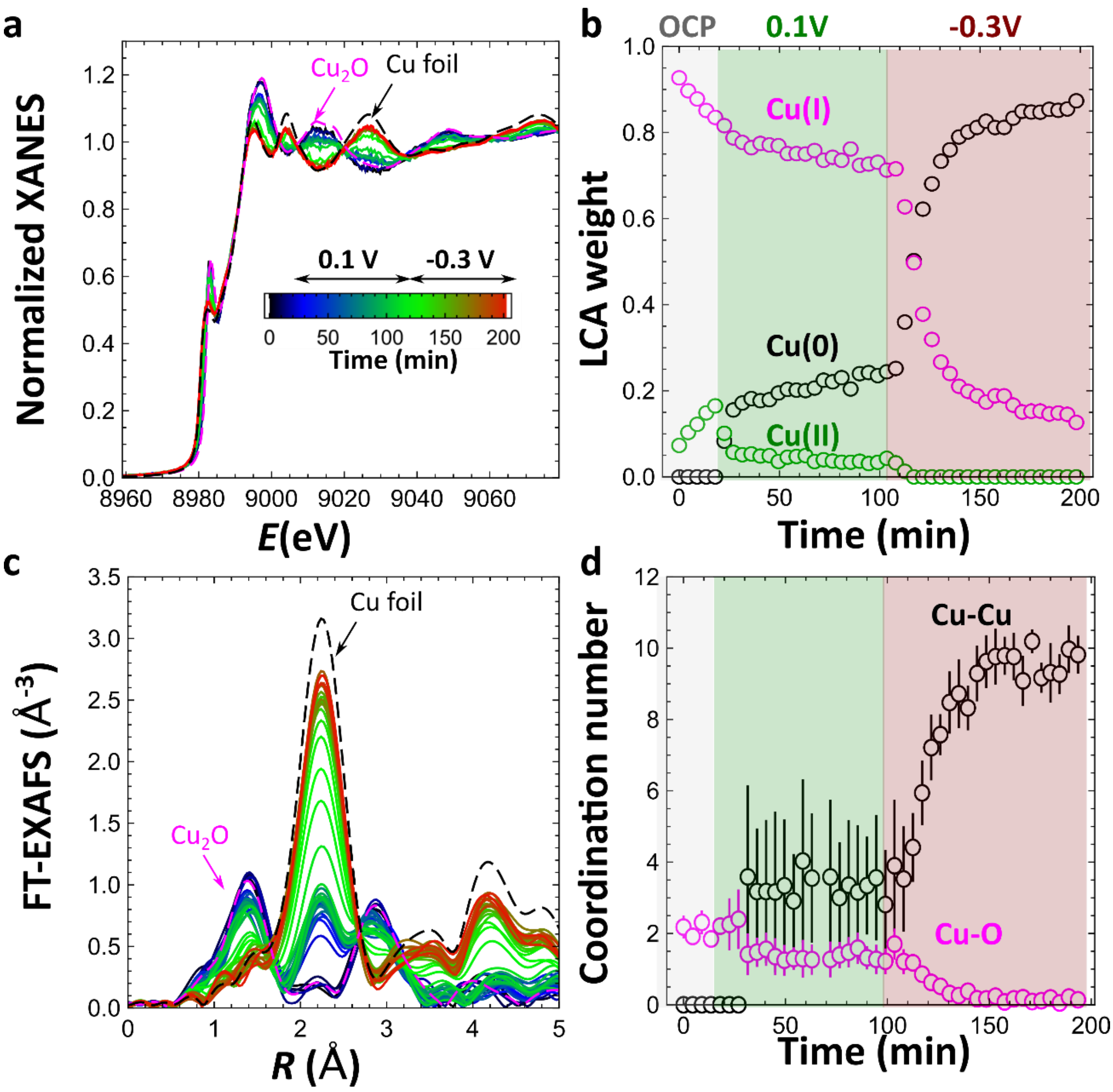

Figure S25. *Operando* XAS experiments data of Cu_2O NCs during NO_2RR. (a) Normalized Cu K-edge XANES spectra collected at OCP, 0.10 V_{RHE} and –0.30 V_{RHE}. (b) Linear combination analysis (LCA) results of the XANES spectra, showing the variation of the Cu oxidation state. (c) Fourier-transformed (FT) Cu K-edge EXAFS spectra of Cu_2O NCs at OCP, 0.10 V_{RHE} and –0.30 V_{RHE}. (d) EXAFS fitting results showing the evolution of Cu-Cu and Cu-O coordination numbers. Electrolyte: pH 12, 0.1 M Na_2SO_4 + 8 mM $NaNO_2$.

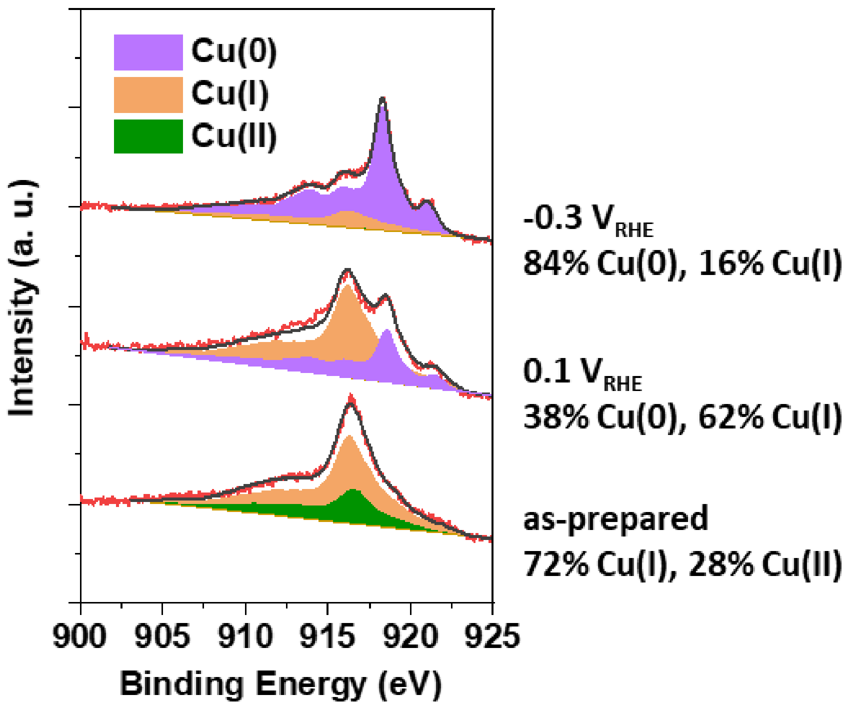

Figure S26. Cu LMM AES data of Cu_2O NCs before and after NO_2RR. The data is collected from quasi *in situ* XPS experiments. Electrolyte: pH 12, 0.1 M Na_2SO_4 + 8 mM $NaNO_2$.

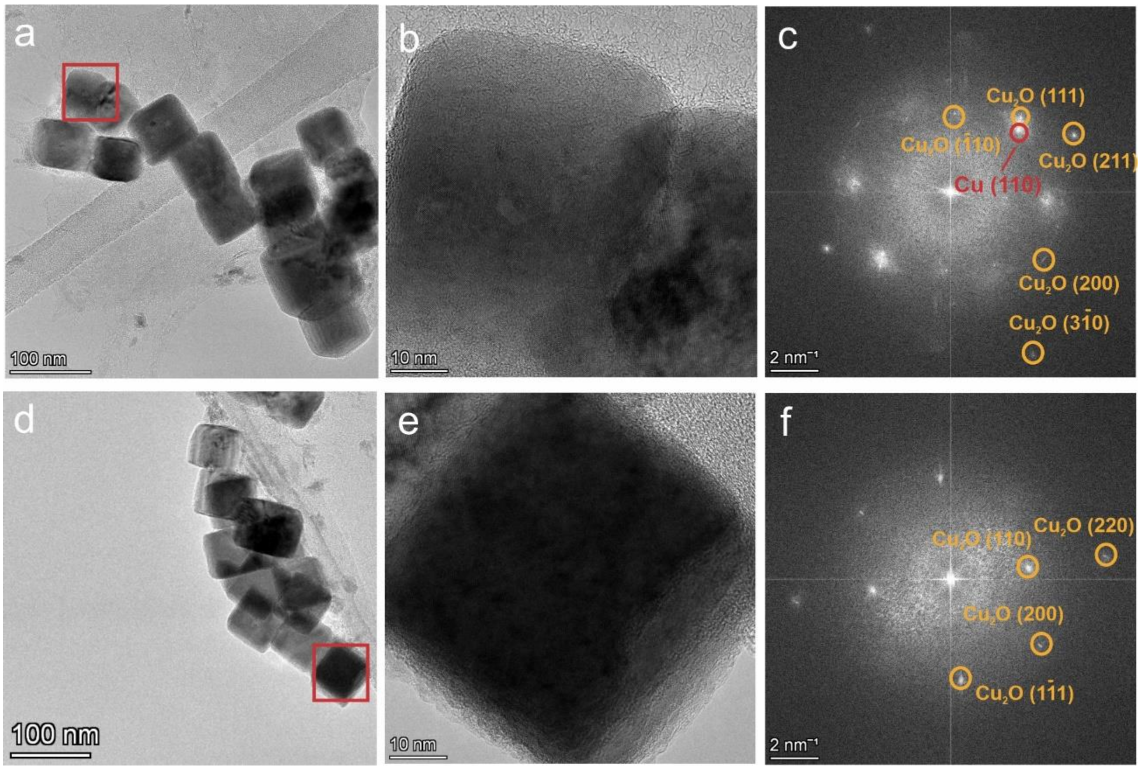

Figure S27. TEM images of Cu_2O NCs after NO_2RR at 0.1 V_{RHE}. (a, d) are TEM images and (b, e) are HRTEM images of Cu_2O NCs. (c) is the FFT patterns of (b) and (f) is the FFT patterns of (e). The red rectangles in (a, d) mark the area for HRTEM. (a-c) are acquired in different regions of TEM grids, comparing to (d-f). Electrolyte: 0.1 M Na_2SO_4 + 8 mM $NaNO_2$ at pH 12.

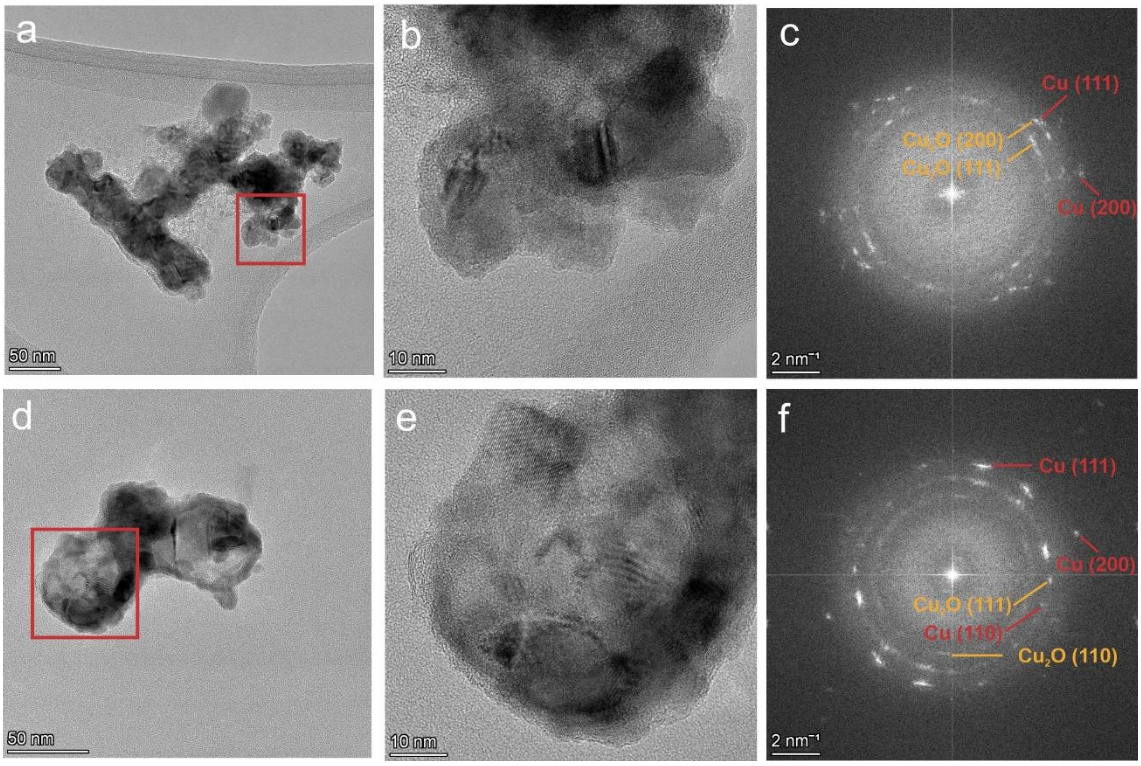

Figure S28. TEM images of Cu_2O NCs after NO_2RR -0.3 V_{RHE}. (a, d) are TEM images and (b, e) are HRTEM images of Cu_2O NCs. (c) is the FFT patterns of (b) and (f) is the FFT patterns of (e). The red rectangles in (a, d) mark the area for HRTEM. (a-c) are acquired in different regions of TEM grids, comparing to (d-f). Electrolyte: 0.1 M Na_2SO_4 + 8 mM $NaNO_2$ at pH 12.

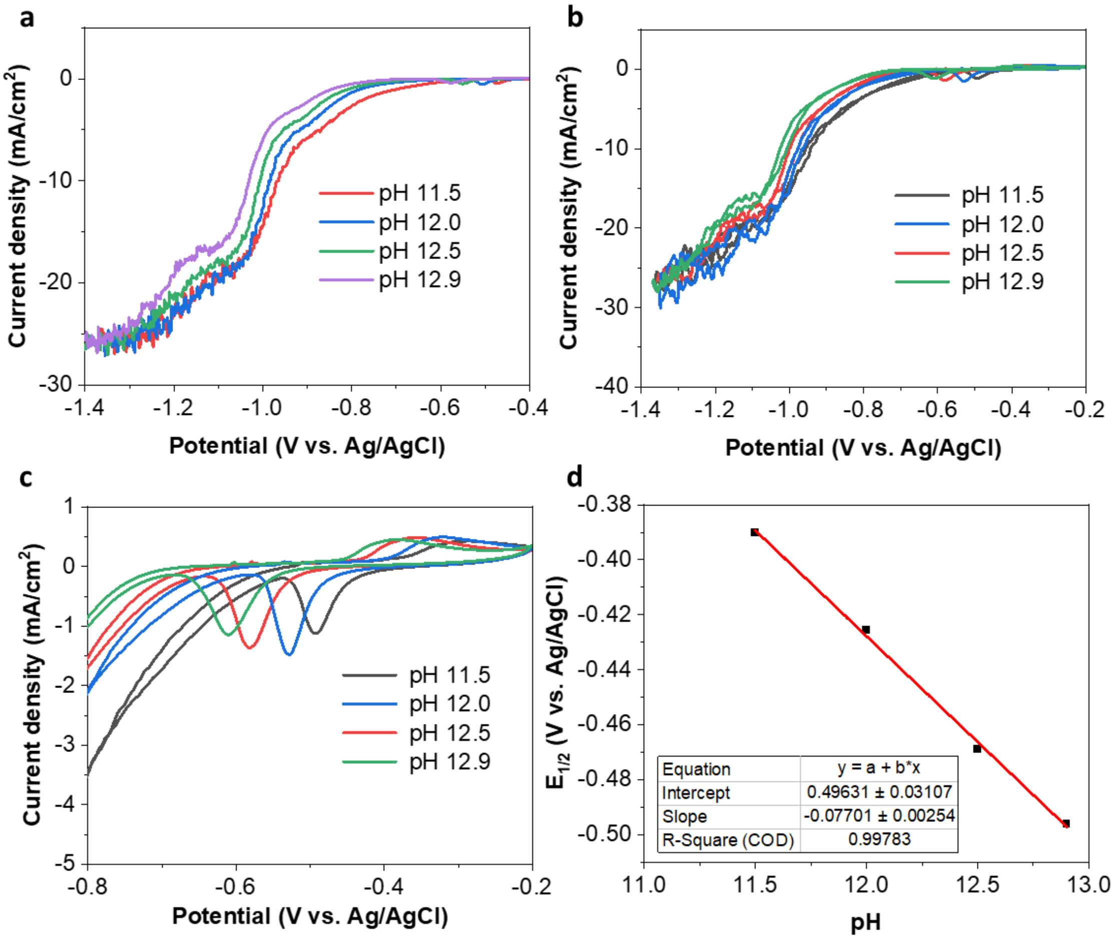

Figure S29. Electrochemical behavior of Cu_2O NCs for NO_3RR. (a) LSVs of Cu_2O NCs at different pH from 11.5 to 12.9, scan rate: 5 mV/s. (b) Cyclic voltammetry (scan rate: 20 mV/s) of Cu_2O NCs at different pH values (from 11.5 to 12.9). (c) Enlarged graph of (b) from -0.2 to -0.8 V vs. Ag/AgCl. (d) The linear fit of redox potential (average of cathodic and anodic peak potentials) on pH values. Electrolyte: 0.1 M Na_2SO_4 + 8 mM $NaNO_3$. The data were recorded under stirring.

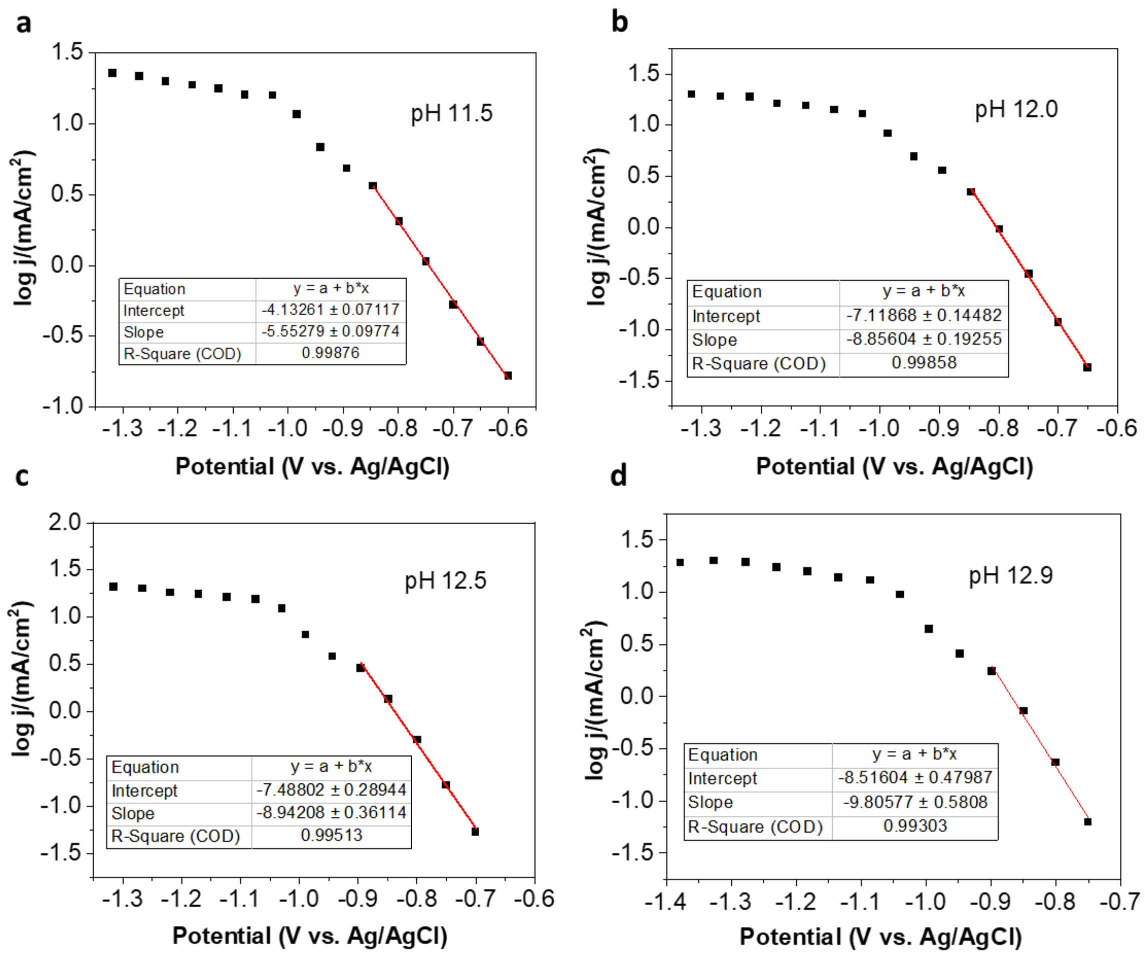

Figure S30. Staircase voltammetry log j – V curves and the corresponding Tafel plots fitting for NO_3RR. (a) pH 11.5; (b) pH 12.0; (c) pH 12.5; (d) pH 12.9. Electrolyte: 0.1 M Na_2SO_4 + 8 mM $NaNO_3$.

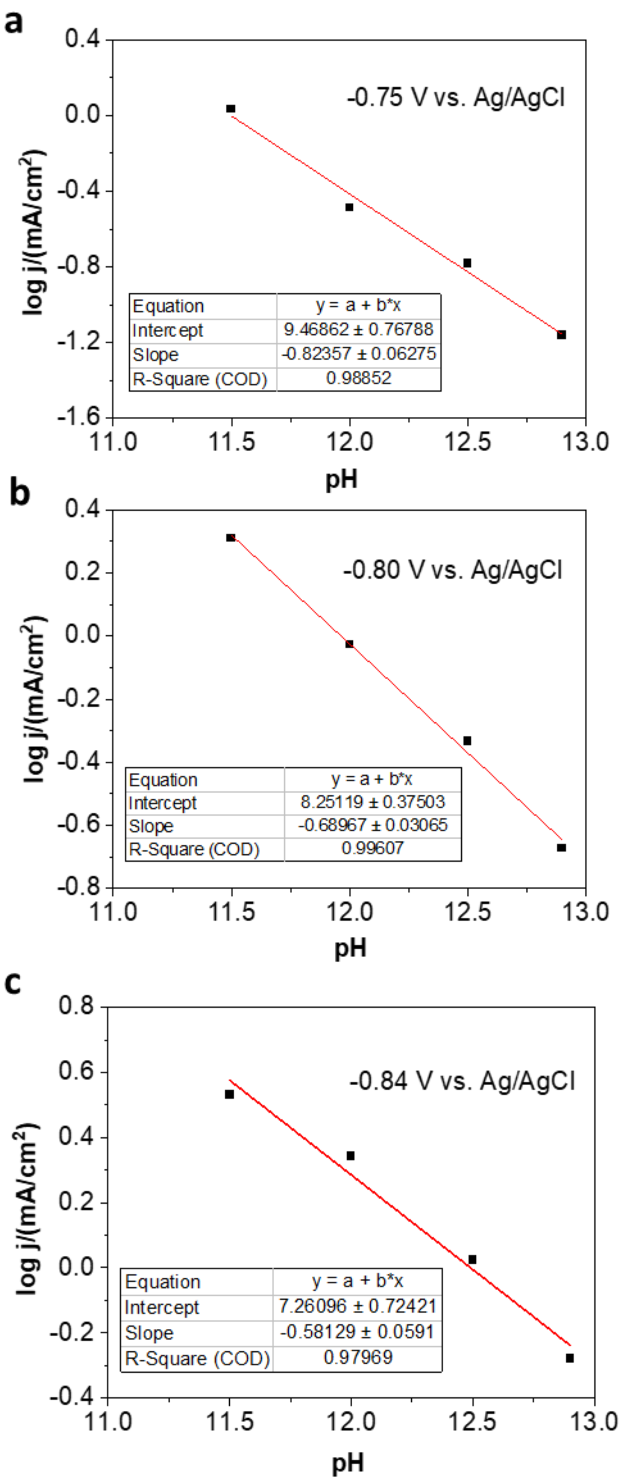

Figure S31. log j – pH curves derived from staircase voltammetry log j – V curves and the corresponding linear fitting. (a) -0.75 V vs. Ag/AgCl; (b) -0.80 V vs. Ag/AgCl; (c) -0.84 V vs. Ag/AgCl. Electrolyte: 0.1 M Na_2SO_4 + 8 mM $NaNO_3$. The order of proton is determined as 0.70 ± 0.12.

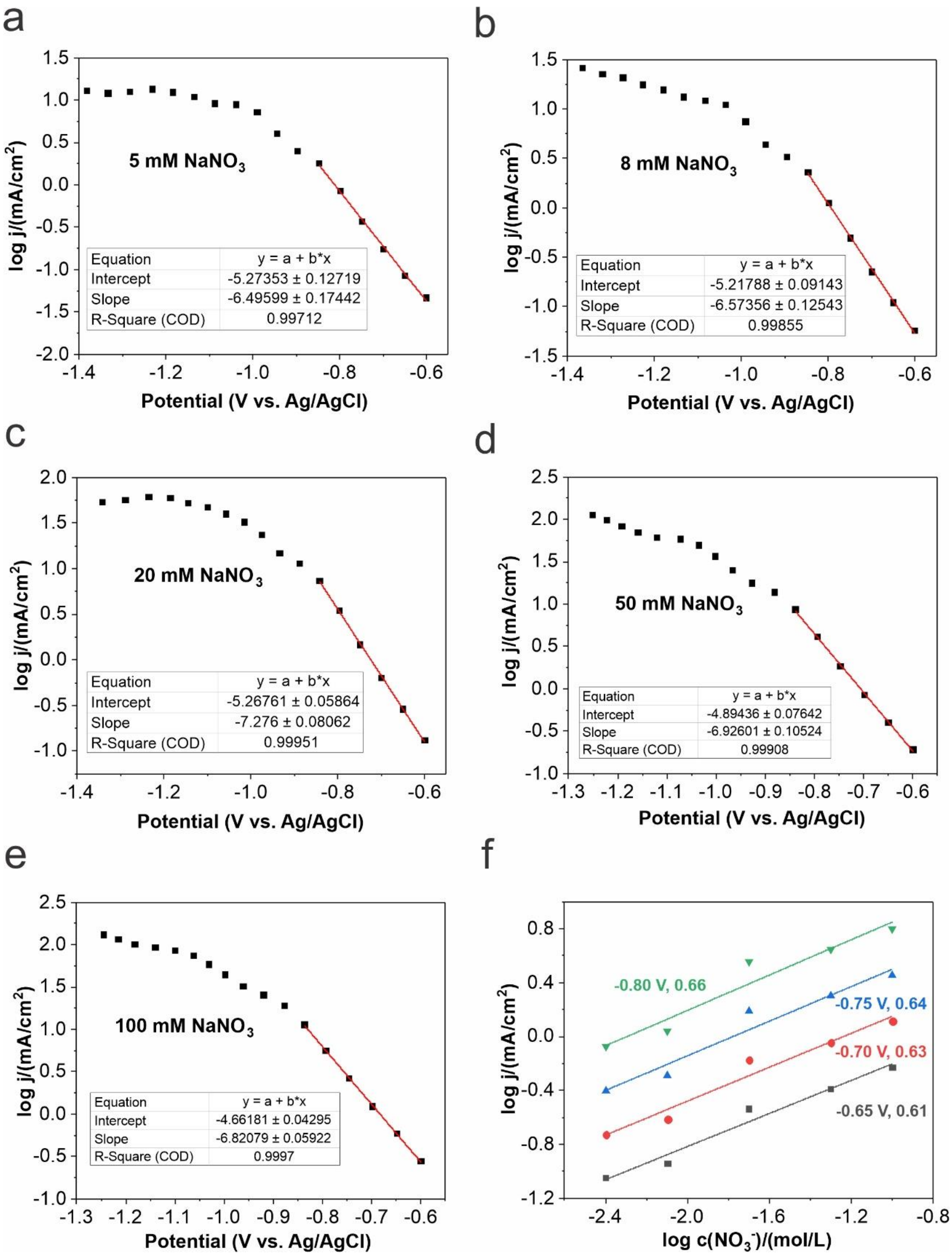

Figure S32. Nitrate reduction performance of Cu_2O NCs in nitrate with different concentrations. The staircase voltammetry log j – V curves are measured and the corresponding Tafel plots are fitted in an electrolyte with different nitrate concentrations: (a) 5 mM $NaNO_3$; (b) 8 mM $NaNO_3$; (c) 20 mM $NaNO_3$; (d) 50 mM $NaNO_3$; (e) 100 mM $NaNO_3$. Electrolyte: pH 12, 0.1 M Na_2SO_4. (f) The log j – log c(NO_3^-) plots in different potential (vs. Ag/AgCl), derived from Tafel plots in (a-e). The order of nitrate is determined as 0.64 ± 0.02.

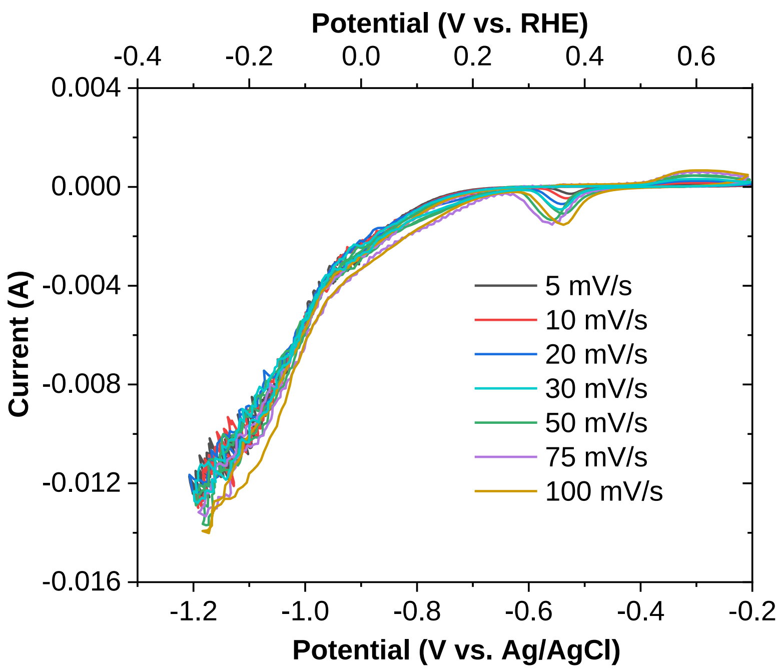

Figure S33. Scan rate dependent CVs of Cu_2O NCs at pH 12.0 in solution with stirring. Electrolyte: pH 12, 0.1 M Na_2SO_4 + 8 mM $NaNO_3$.

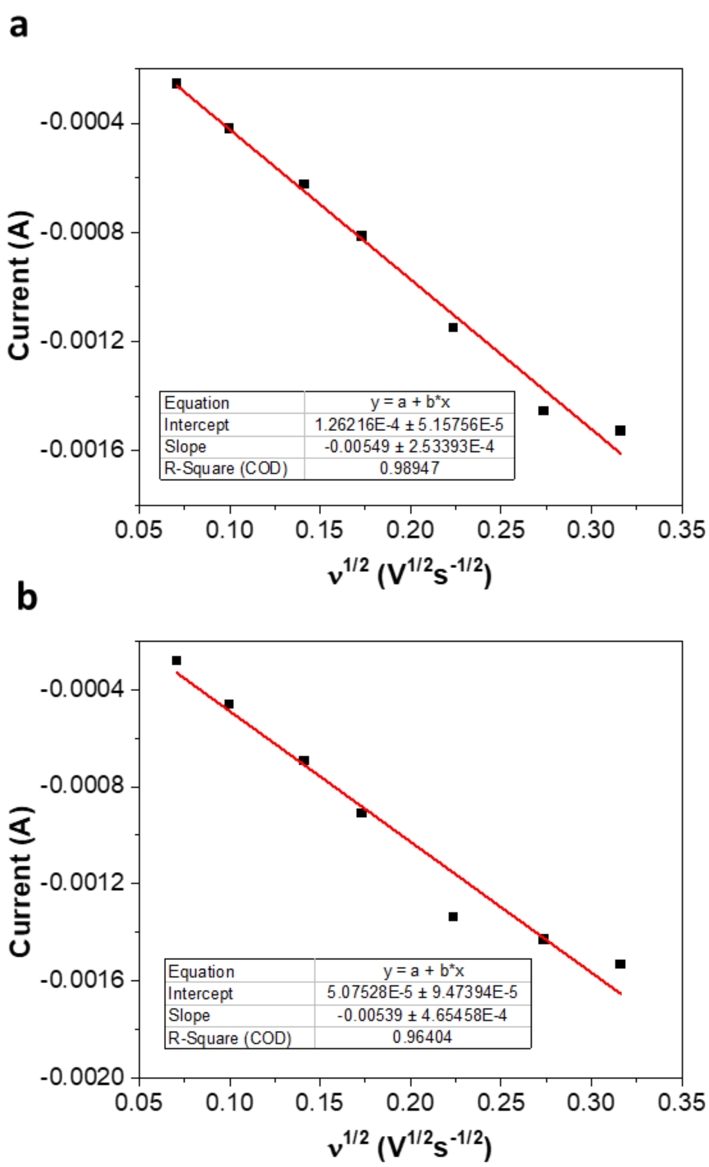

Figure S34. Scan rate dependent electrochemical behavior of Cu_2O NCs for NO_3RR. (a) Linear fitting plot of the cathodic peak current of Cu(I)/Cu(0) redox peak (**R1** of **Figure 4b, main text**) versus the square root of scan rate. (b) Linear fitting plot of cathodic peak current of Cu(I)/Cu(0) redox peak in **Figure S33** versus the square root of scan rate.

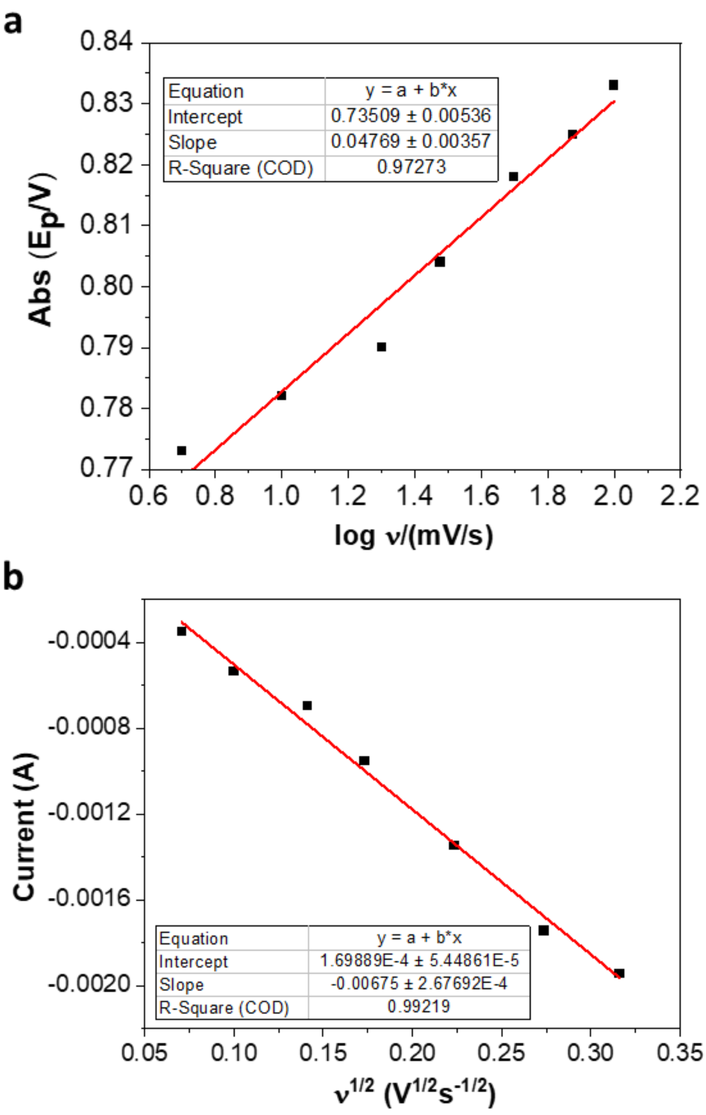

Figure S35. Scan rate dependent electrochemical behavior of Cu_2O NCs for NO_3RR. (a) Linear fitting plot of absolute peak potential of **R2** (**Figure 4b, main text**) versus the logarithm of scan rate. (b) Linear fitting plot of the catalytic peak current of **R2** (**Figure 4b, main text**) versus the square root of scan rate. The fitting results indicated that the electron transfer number is 1.7.

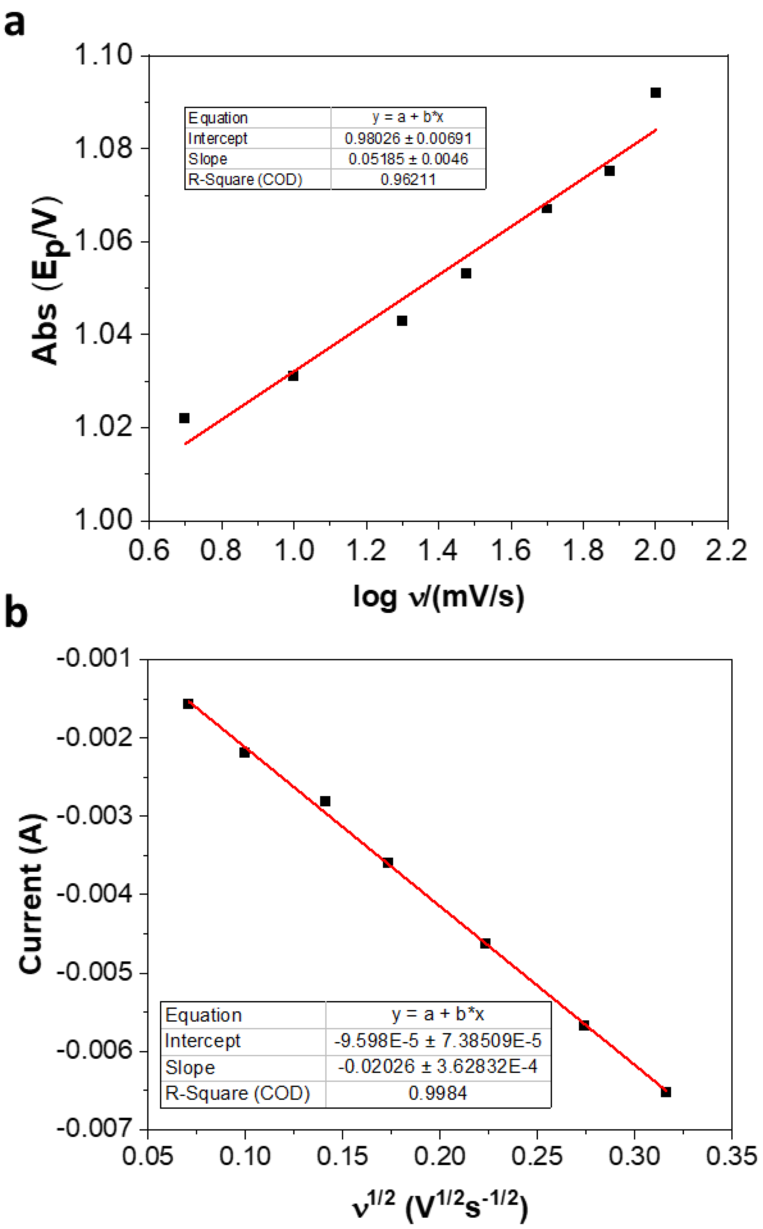

Figure S36. Scan rate dependent electrochemical behavior of Cu_2O NCs for NO_3RR. (a) Linear fitting plot of the absolute peak potential of **R3** (**Figure 4b, main text**) versus the logarithm of scan rate. (b) Linear fitting plot of catalytic peak current of **R3** (**Figure 4b, main text**) versus the square root of scan rate. The fitting results indicate that the electron transfer number is 5.5. Three independent experiments indicated that the ratio of n(R3)/n(R2) is 2.9±0.2.

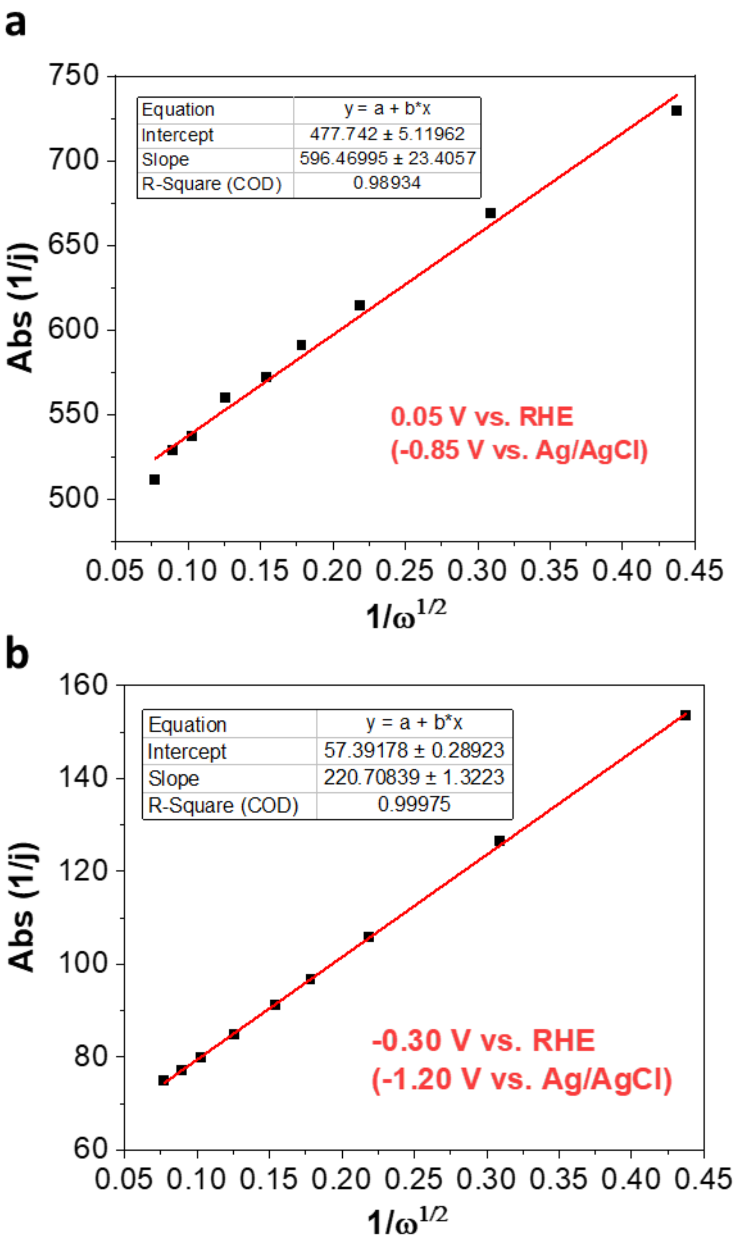

Figure S37. Rotating disk electrode (RDE) electrochemical behavior of Cu_2O NCs for NO_3RR. (a) Linear fits of the reciprocal of current density versus the reciprocal of square root of rotating rate (Koutecký–Levich equation). Potential: 0.05 V_{RHE}. From the slope of the fitted plot, the total electron transfer number is calculated as 2.3. (c) Linear fit of the reciprocal current density versus the reciprocal of square root of rotating rate (Koutecký–Levich equation). Potential: -0.30 V_{RHE}. From the slope of the fitted plot, the total electron transfer number is calculated as 6.1. Electrolyte: pH 12, 0.1 M Na_2SO_4 + 8 mM $NaNO_3$. The fitting is based on data in **Figure 4c, main text**. Three independent experiments showed that the total electron transfer number at 0.05 V_{RHE} is 1.9 ± 0.3; while the total electron transfer number at -0.30 V_{RHE} is 6.2 ± 0.2.

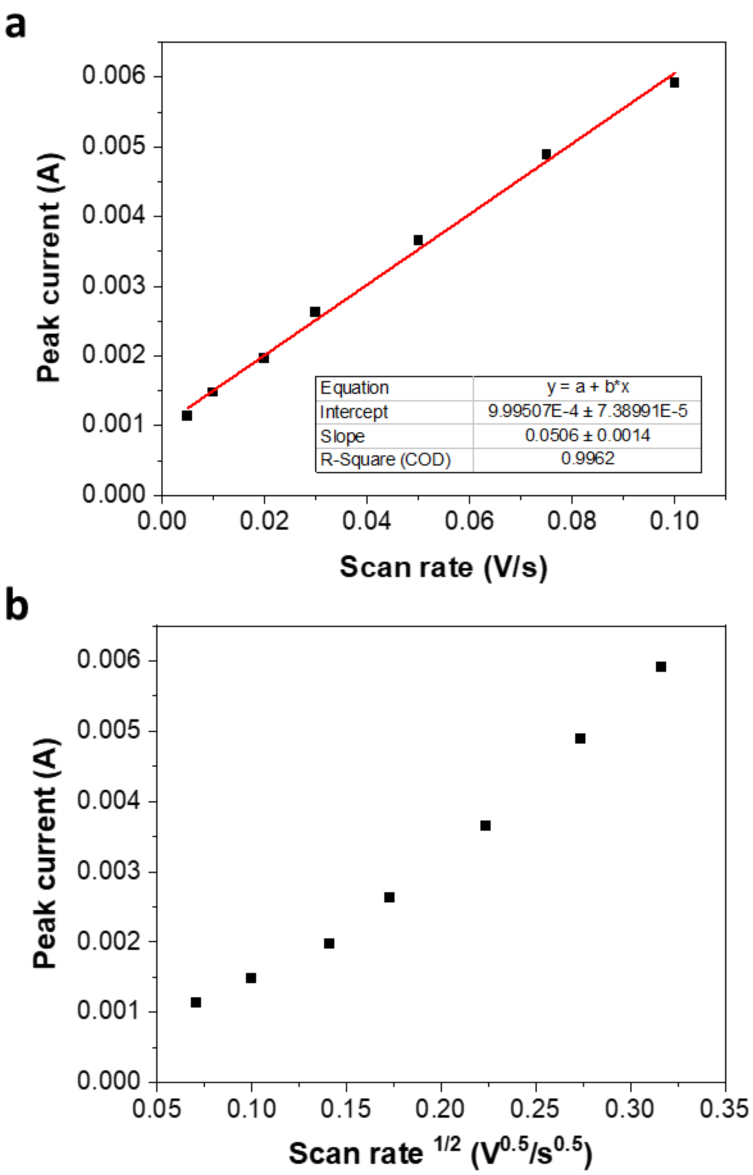

Figure S38. Scan rate dependent electrochemical behavior of Cu_2O NCs for NO_3RR. (a) Linear fitting plot of absolute peak current of **R4** (**Figure 4b, main text**) versus the scan rate. (b) The peak current of **R4** (**Figure 4b, main text**) is not linearly increased with the square root of scan rate. Therefore, **R4** (**Figure 4b, main text**) is related to desorption of surface adsorbed species.

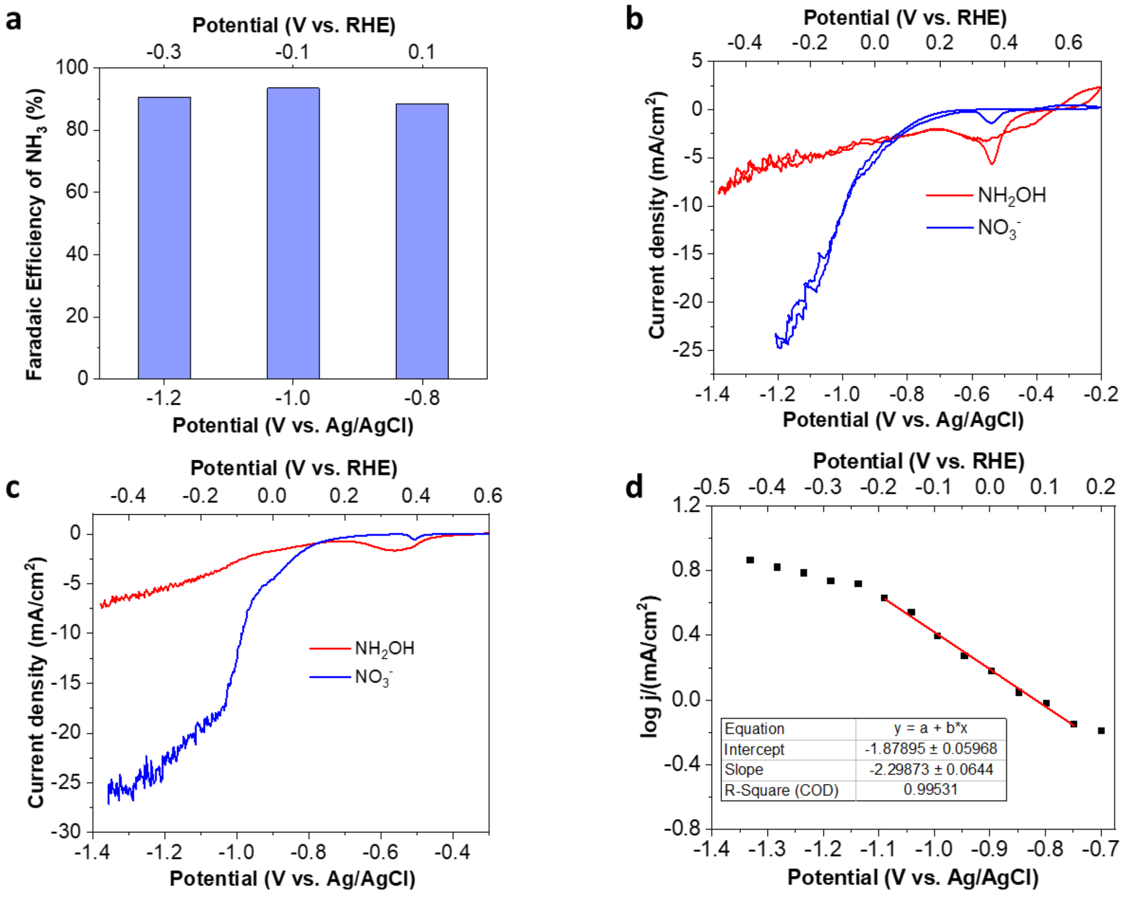

Figure S39. Electrochemical behavior of Cu_2O NCs for reduction of NH_2OH. (a) Faradaic efficiency of ammonia at various applied potentials after 1 h electrolysis. Electrolyte: pH 12, 0.1 M Na_2SO_4 + 8 mM NH_2OH. (b) CVs of Cu_2O NCs in pH 12.0, 0.1 M Na_2SO_4, containing 8 mM NH_2OH (red), 8 mM $NaNO_3$ (blue). The scan rate is 20 mV/s. (c) LSVs of Cu_2O NCs in pH 12.0, 0.1 M Na_2SO_4, containing 8 mM NH_2OH (red), 8 mM $NaNO_3$ (blue). The scan rate is 5 mV/s. (d) Steady state log j – V curves and the corresponding Tafel plots fitting. Electrolyte: pH 12, 0.1 M Na_2SO_4 + 8 mM NH_2OH. The solution is stirring during all the measurements.

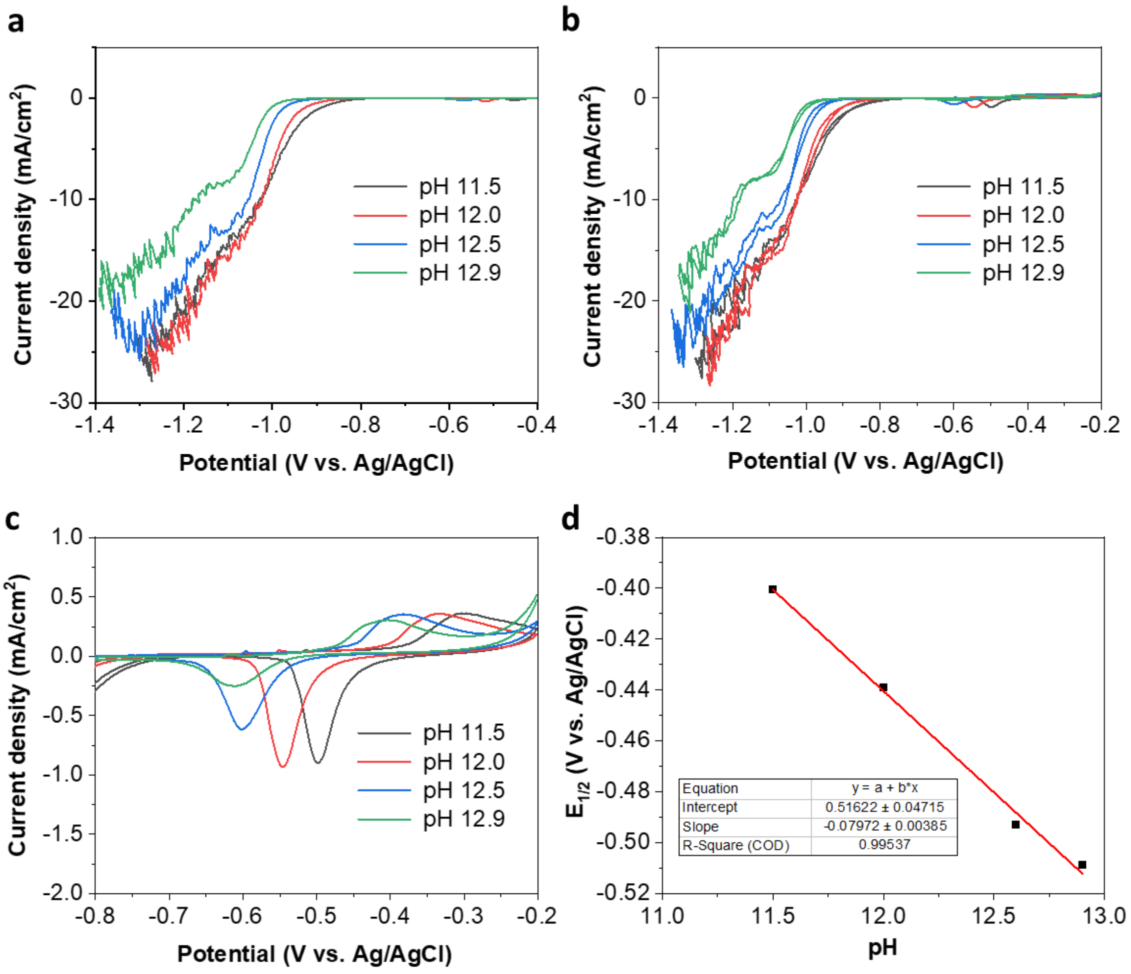

Figure S40. Electrochemical behavior of Cu_2O NCs for NO_2RR. (a) LSVs of Cu_2O NCs at different pH from 11.5 to 12.9, scan rate: 5 mV/s. (b) Cyclic voltammetry (scan rate: 20 mV/s) of Cu_2O NCs at different pH values (from 11.5 to 12.9). (c) Enlarged graph of (b) from -0.2 to -0.8 V vs. Ag/AgCl. (d) The linear fitting of redox potential (average of cathodic and anodic peak potentials) on pH values. Electrolyte: 0.1 M Na_2SO_4 + 8 mM $NaNO_2$. The data were recorded under stirring.

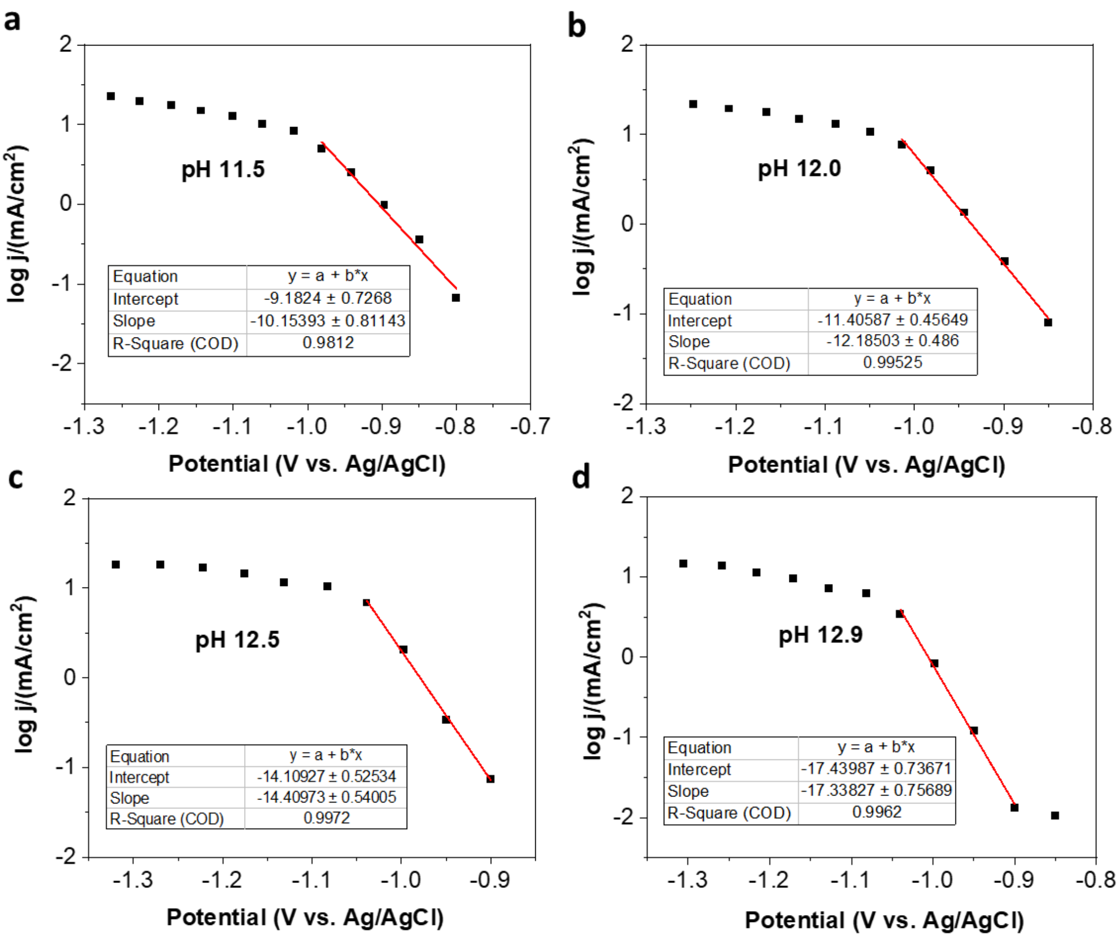

Figure S41. Staircase voltammetry log j – V curves and the corresponding Tafel plots fitting for NO_2RR. (a) pH 11.5; (b) pH 12.0; (c) pH 12.5; (d) pH 12.9. Electrolyte: 0.1 M Na_2SO_4 + 8 mM $NaNO_2$.

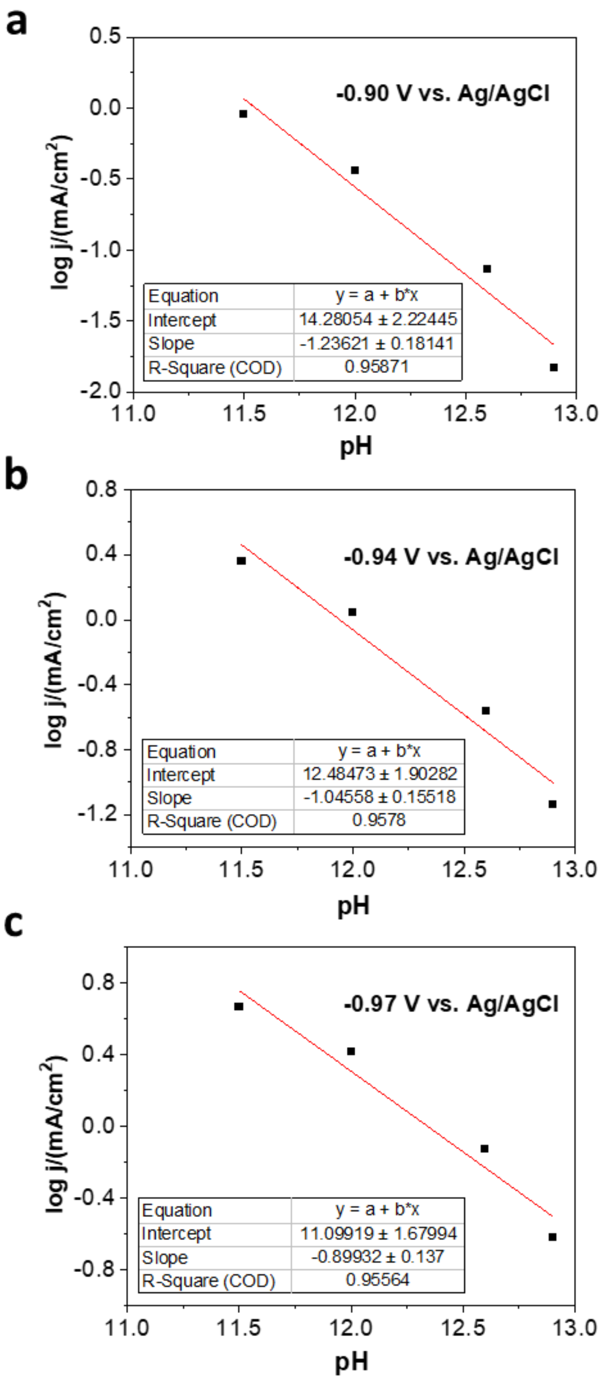

Figure S42. log j – pH curves derived from staircase voltammetry log j – V curves and the corresponding linear fitting for NO_2RR. (a) -0.90 V vs. Ag/AgCl; (b) -0.94 V vs. Ag/AgCl; (c) -0.97 V vs. Ag/AgCl. Electrolyte: 0.1 M Na_2SO_4 + 8 mM $NaNO_2$. The order of proton is determined as 1.06 ± 0.17.

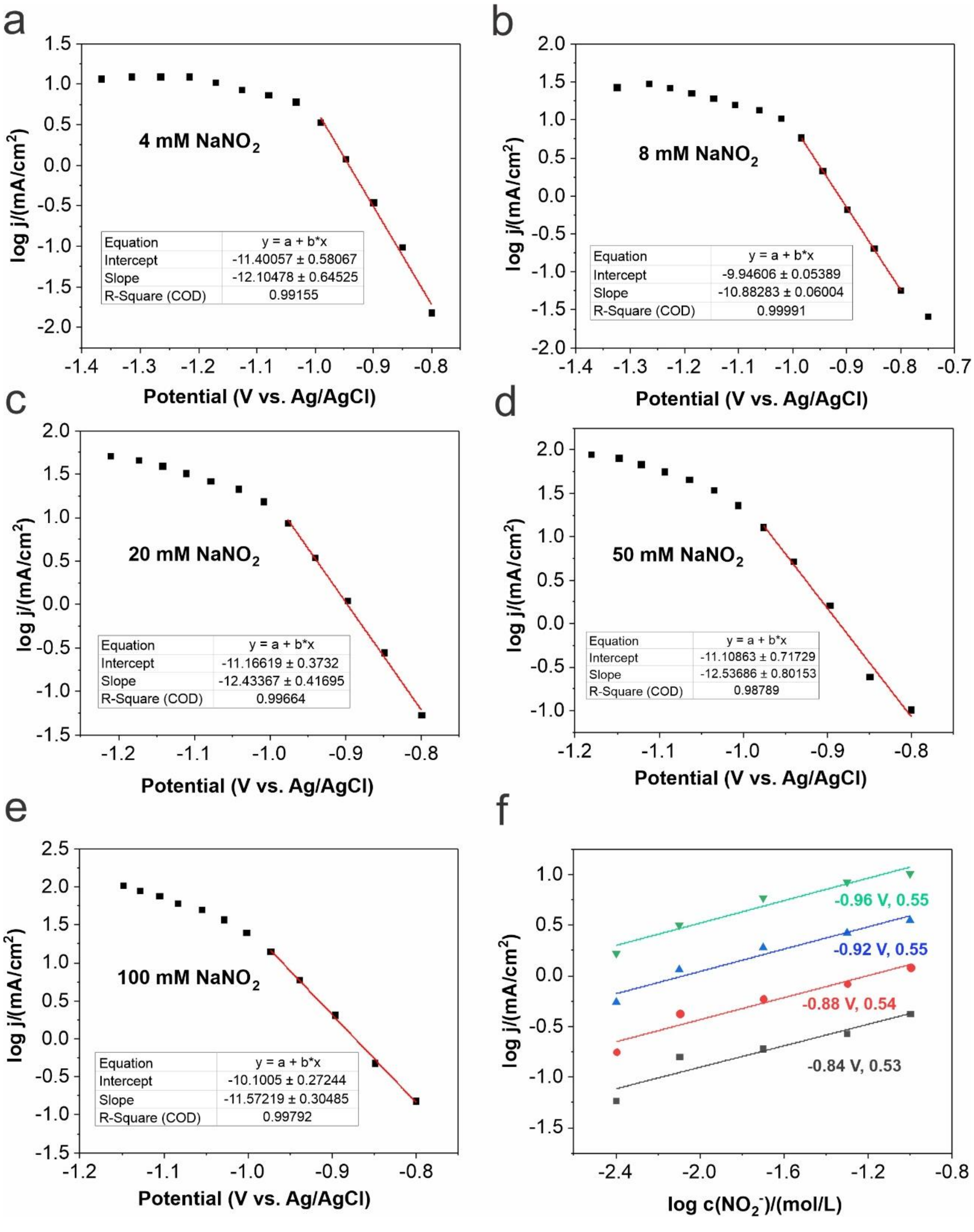

Figure S43. Nitrite reduction performance of Cu_2O NCs in nitrite with different concentration. The staircase voltammetry log j – V curves are measured and the corresponding Tafel plots are fitted in electrolyte with different nitrite concentration: (a) 4 mM $NaNO_2$; (b) 8 mM $NaNO_2$; (c) 20 mM $NaNO_2$; (d) 50 mM $NaNO_2$; (e) 100 mM $NaNO_2$. Electrolyte: pH 12, 0.1 M Na_2SO_4. (f) The log j – log c(NO_2^-) plots in different potential (vs. Ag/AgCl), derived from Tafel plots in (a-e). The order of nitrite is determined as 0.54 ± 0.01.

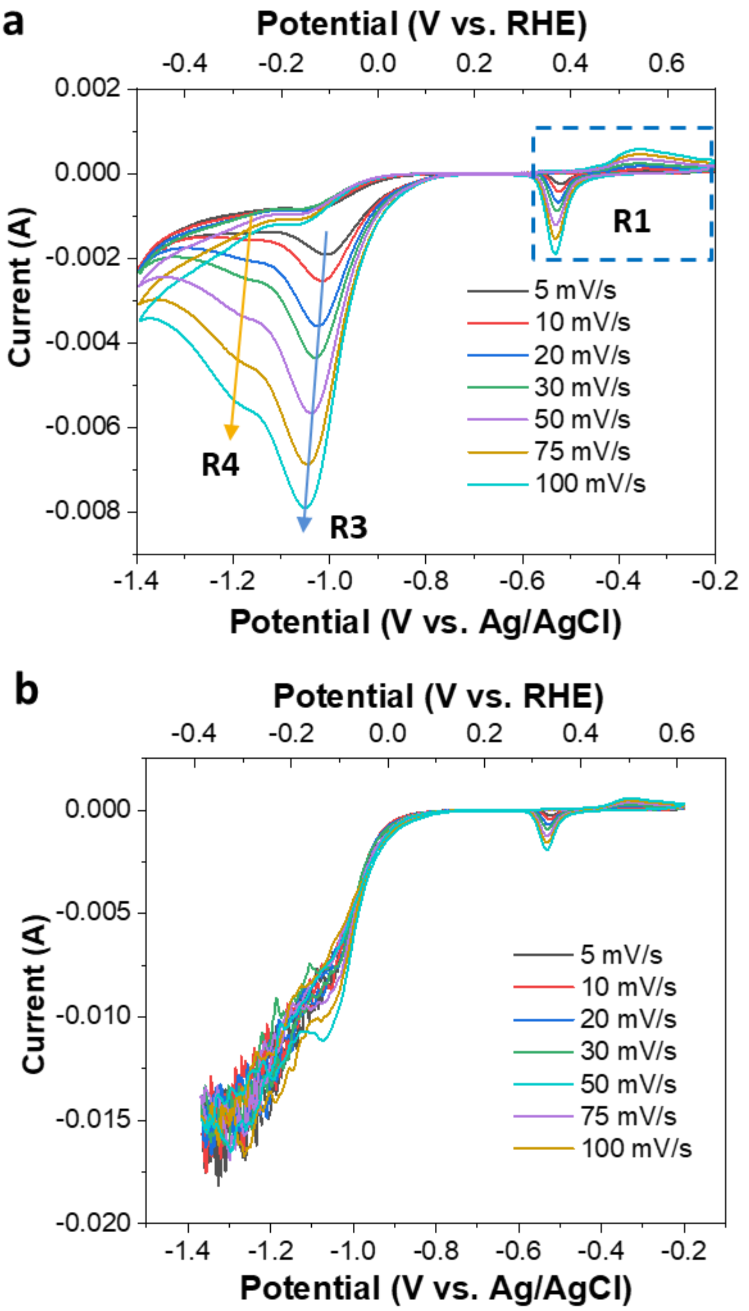

Figure S44. Scan rate dependent CVs of Cu_2O NCs for NO_2RR. (a) without stirring; (b) with stirring. Electrolyte: pH 12, 0.1 M Na_2SO_4 + 8 mM $NaNO_2$.

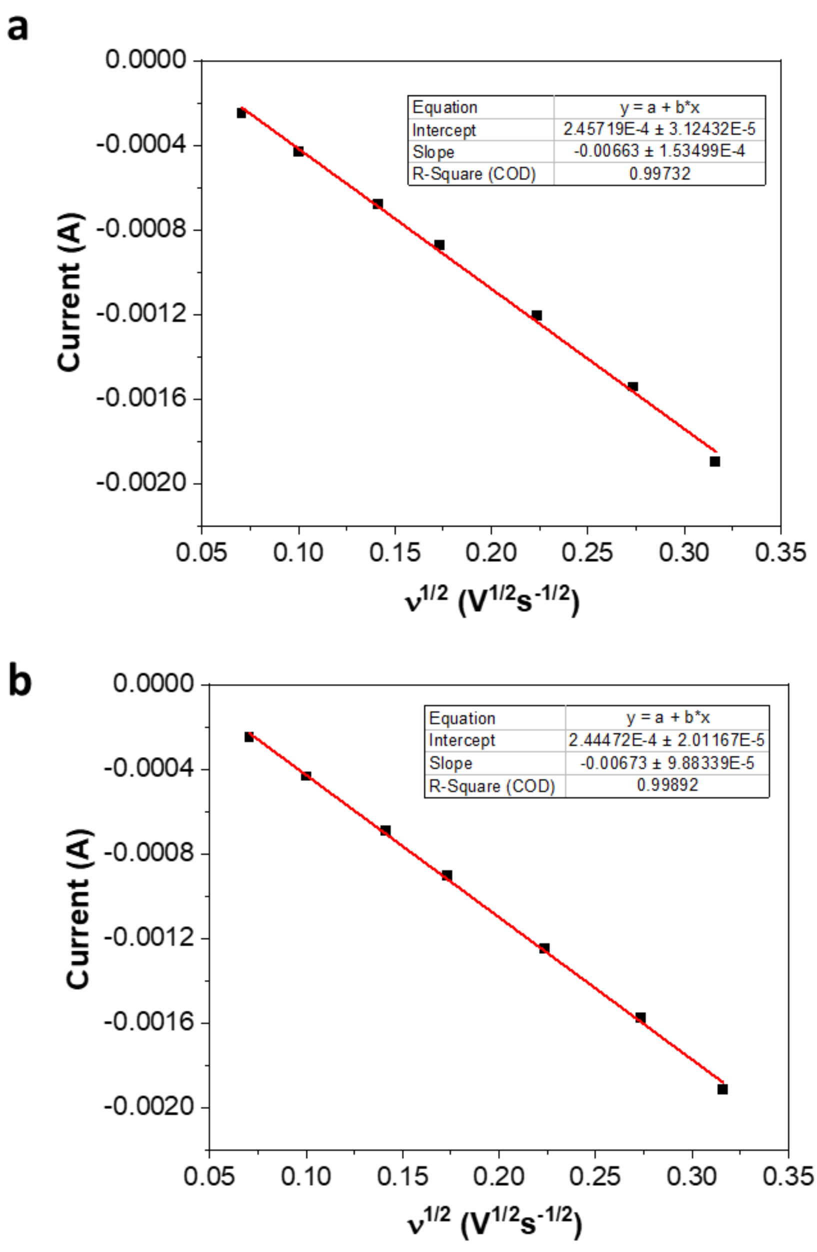

Figure S45. Scan rate dependent electrochemical behavior of Cu_2O NCs for NO_2RR. (a) Linear fitting plot of cathodic peak current of Cu(I)/Cu(0) redox peak (**Figure S44a**) versus the square root of scan rate. (b) Linear fitting plot of cathodic peak current of Cu(I)/Cu(0) redox peak (**Figure S44b**) versus the square root of scan rate.

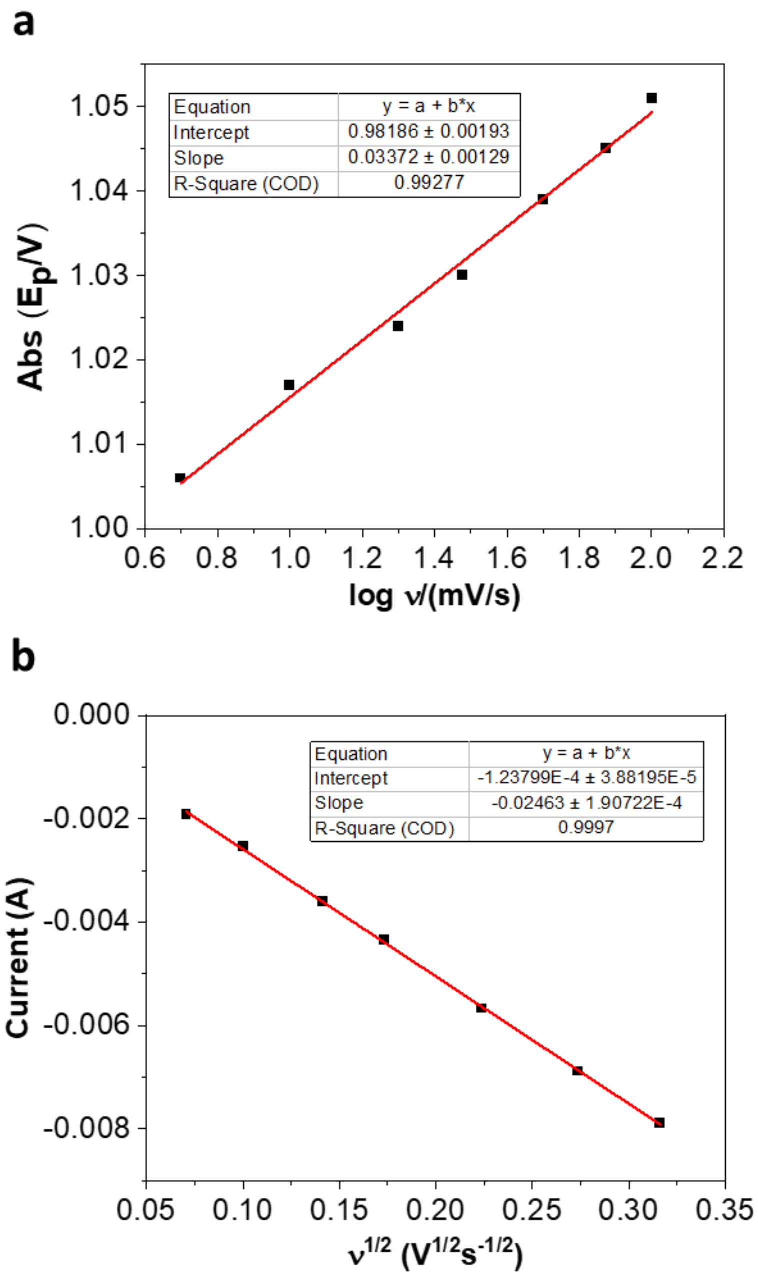

Figure S46. Scan rate dependent electrochemical behavior of Cu_2O NCs for NO_2RR. (a) Linear fitting plot of catalytic peak current of the catalytic peak **R3** in **Figure S44a** versus the square root of scan rate. (b) Linear fitting plot of absolute peak potential of the catalytic peak **R3** in **Figure S44a** versus the logarithm of scan rate. The fitting results indicated that the electron transfer number is 5.0. Three different independent experiments showed that the fitted electron transfer number is 4.5±0.9.

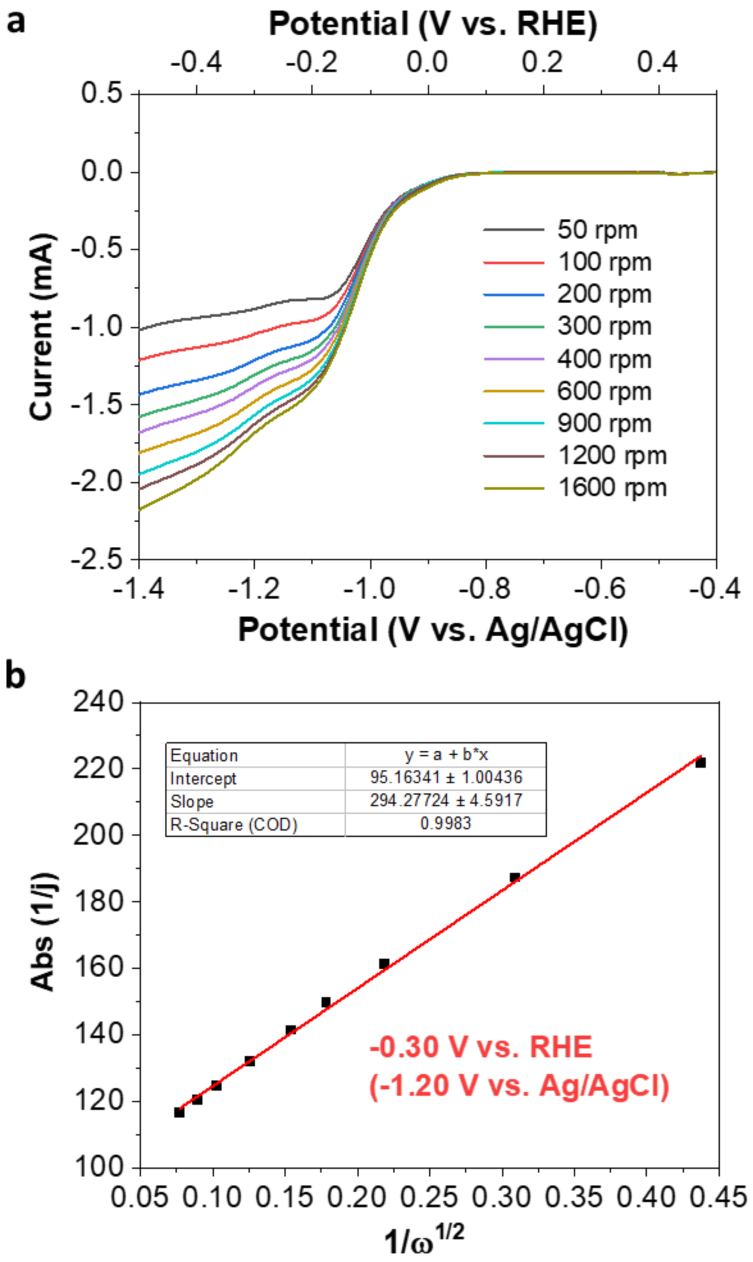

Figure S47. Rotating disk electrode (RDE) electrochemical behavior of Cu_2O NCs for NO_2RR. (a) Linear scan voltammetry (scan rate: 10 mV/s) of Cu_2O NCs on a rotating disk electrode at different rotating rate. Electrolyte: pH 12, 0.1 M Na_2SO_4 + 8 mM $NaNO_2$. (b) Linear fits of reciprocal of current density versus reciprocal of square root of rotating rate (Koutecký–Levich equation). Potential: -0.30 V vs. RHE. From the slope of the fitted plot, the total electron transfer number is 4.3. Three independent experiments showed that the total electron transfer number is 4.2 ± 0.1.

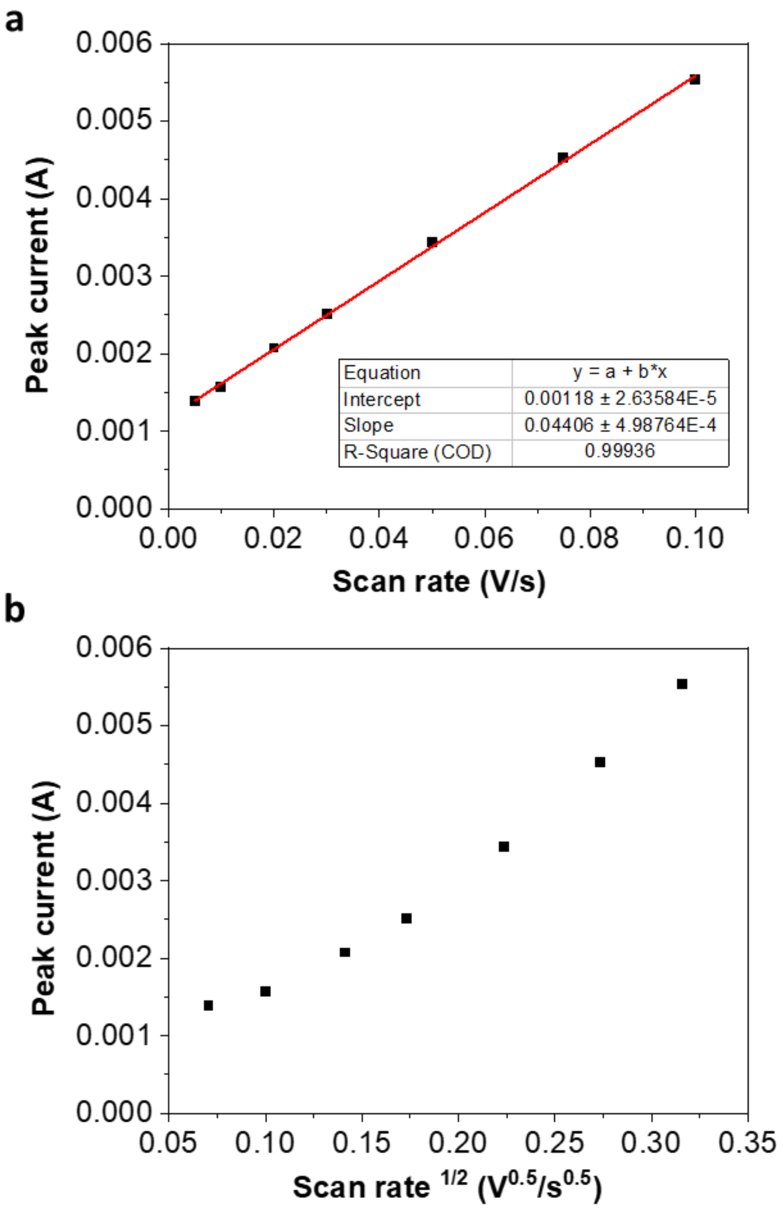

Figure S48. Scan rate dependent electrochemical behavior of Cu_2O NCs for NO_2RR. (a) Linear fitting plot of absolute peak current of **R4** (**Figure S44a**) versus the scan rate. (b) The peak current of **R4** (**Figure S44a**) is not linearly increased with the square root of scan rate. Therefore, **R4** (**Figure S44a**) is related to desorption of surface adsorbed species.

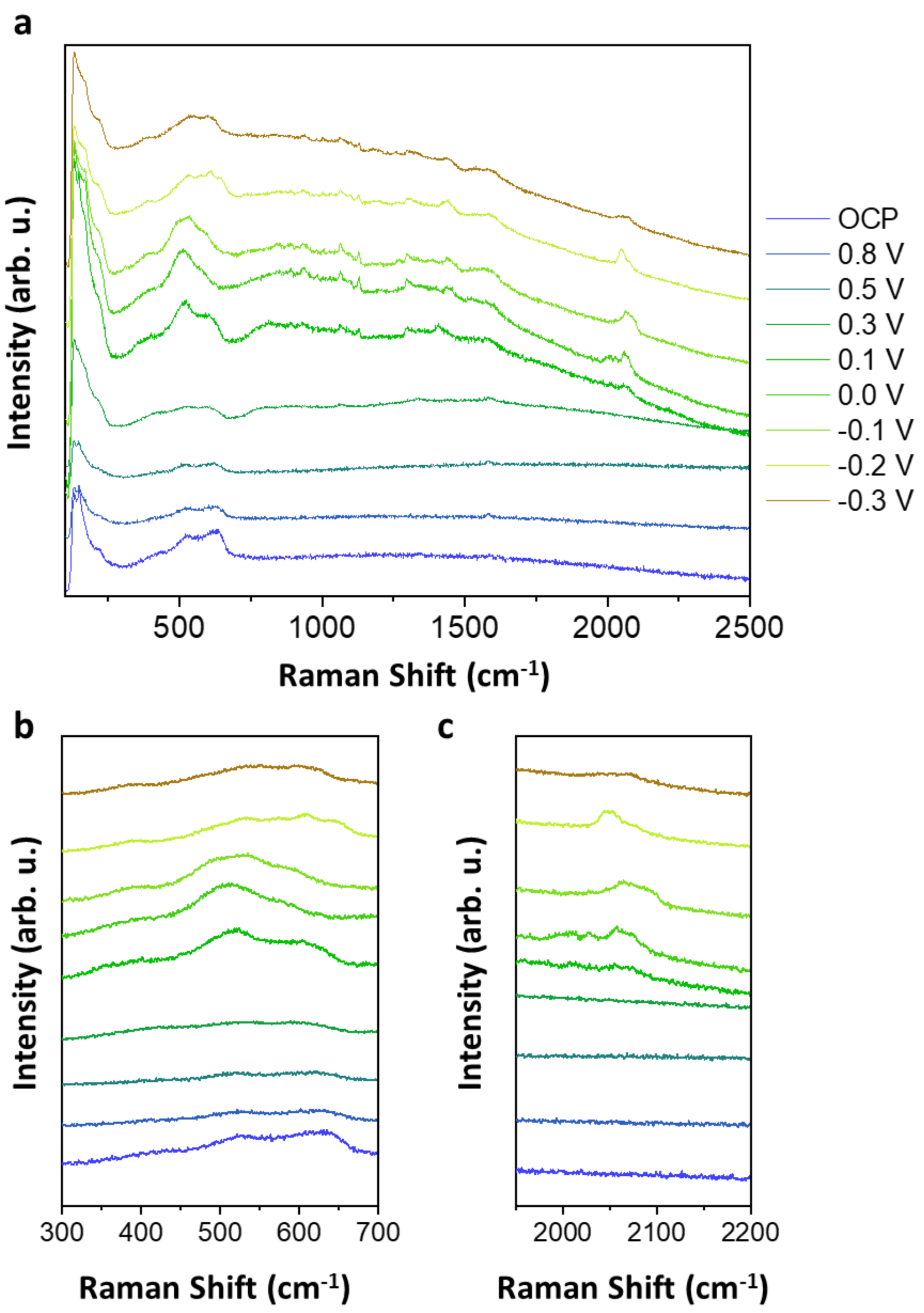

Figure S49. ***In situ*** **Raman spectra of Cu_2O NCs for NO_3RR.** (a) Full spectra measured from 100-2500 cm^{-1} Raman shift. (b) Enlarged spectra in the range of 300-700 cm^{-1}. (c) Enlarged spectra in the range of 1950-2200 cm^{-1}. The applied potentials are ranging from the OCP to −0.3 V_{RHE}. Electrolyte: pH 12, 0.1 M Na_2SO_4 + 8 mM $NaNO_3$.

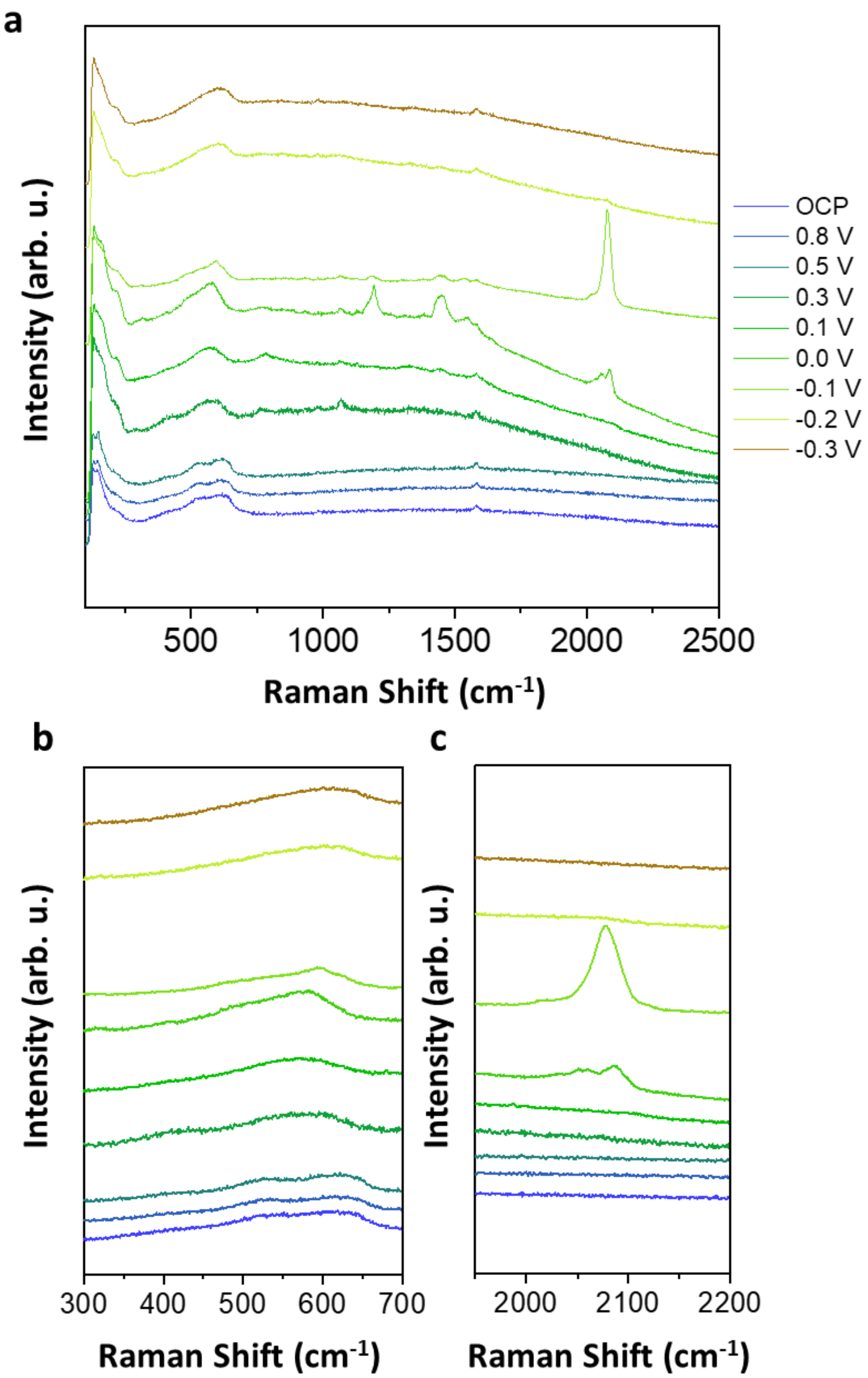

Figure S50. *In situ* Raman spectra of Cu_2O NCs for NO_2RR. (a) Full spectra measured from 100-2500 cm^{-1} Raman shift. (b) Enlarged spectra in the range of 300-700 cm^{-1}. (c) Enlarged spectra in the range of 1950-2200 cm^{-1}. The applied potentials are ranging from the OCP to −0.3 V_{RHE}. Electrolyte: pH 12, 0.1 M Na_2SO_4 + 8 mM $NaNO_2$.

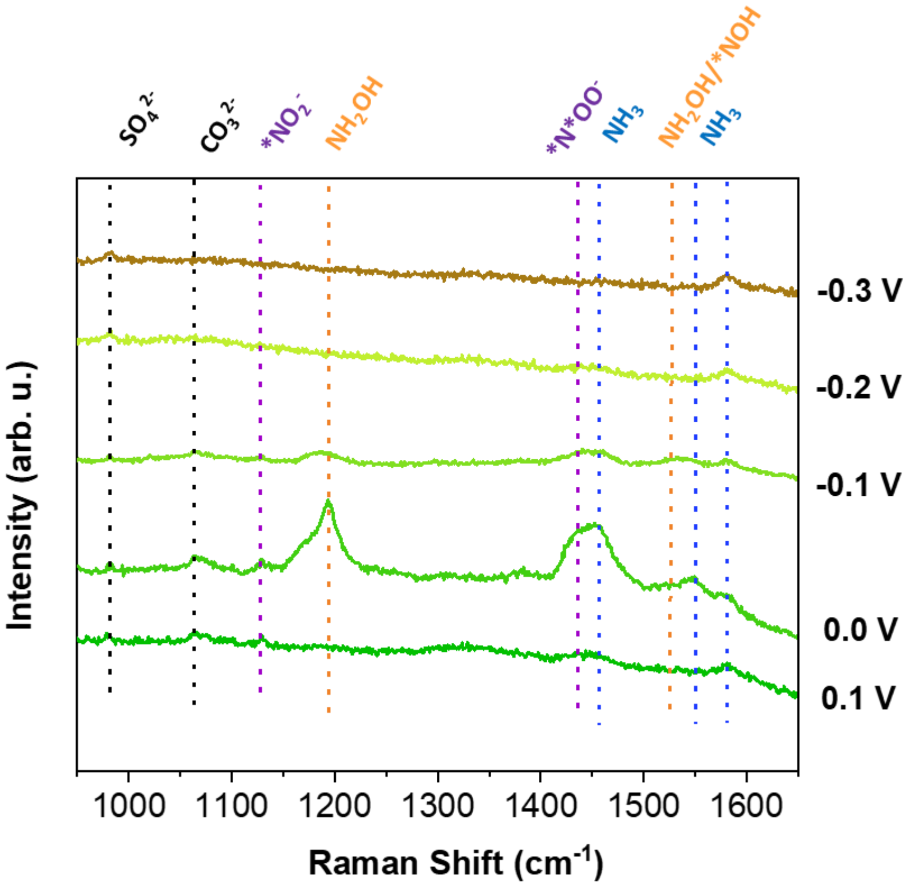

Figure S51. *In situ* Raman spectroscopy of Cu_2O NCs during NO_2RR. Enlarged Raman spectra in the range of 950-1650 cm^{-1} Raman shift, with applied potentials ranging from the 0.1 to −0.3 V_{RHE}. Full spectra are shown in **Figure S50a**. The applied potentials are ranging from the OCP to −0.3 V_{RHE}. Electrolyte: pH 12, 0.1 M Na_2SO_4 + 8 mM $NaNO_2$. The NO_2^-, NH_2OH, and NH_3 related peaks are labelled with violet, orange, and blue dotted line, respectively. Peaks at 983 and 1065 cm^{-1} correspond to SO_4^{2-} and CO_3^{2-} in solution (labelled with black dotted line), respectively, as they are always appeared once the electrolyte contains Na_2SO_4 (pH 12).

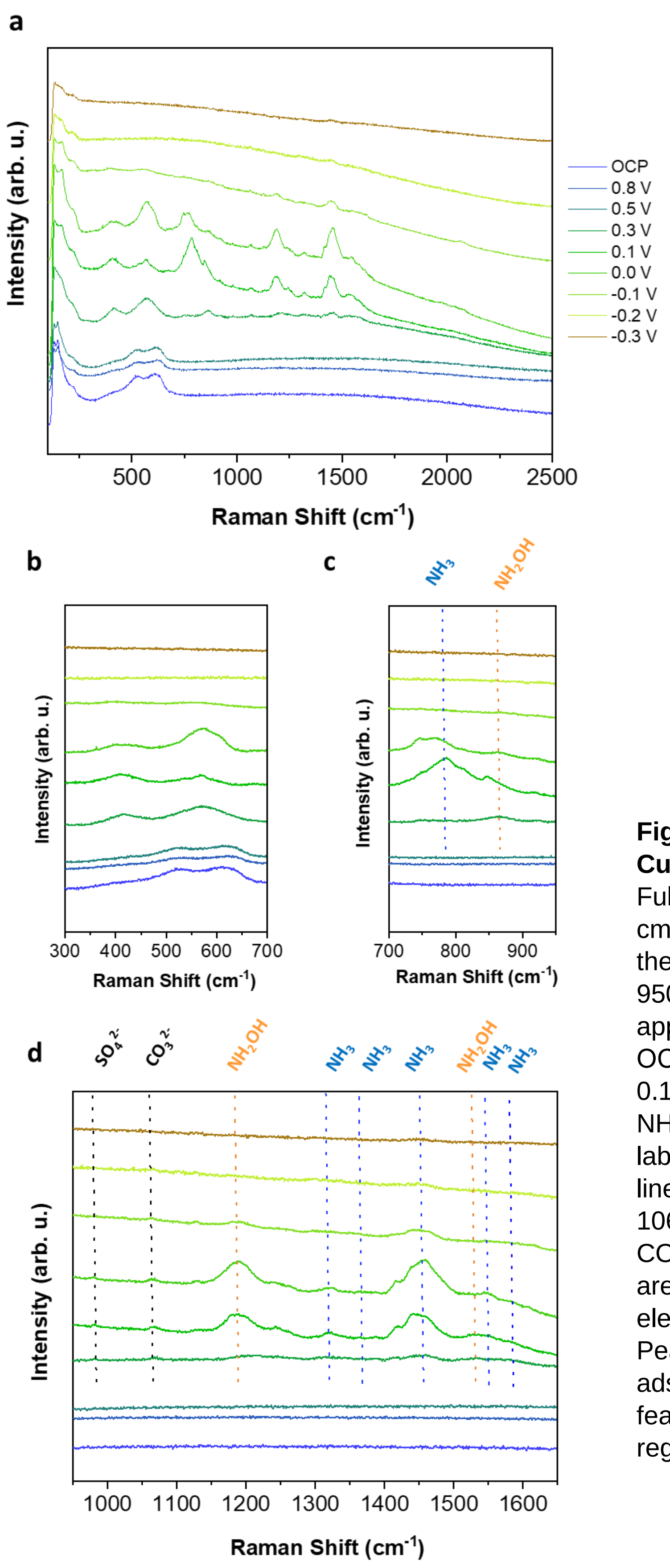

Figure S52. ***In situ*** **Raman spectra of Cu_2O NCs for NH_2OH reduction.** (a) Full spectra measured from 100-2500 cm^{-1} Raman shift. Enlarged spectra in the range of (b) 300-700 cm^{-1}, (c) 700-950 cm^{-1} and (d) 950-1650 cm^{-1}. The applied potentials are ranging from the OCP to −0.3 V_{RHE}. Electrolyte: pH 12, 0.1 M Na_2SO_4 + 8 mM NH_2OH. The NH_2OH and NH_3 related peaks are labelled with orange and blue dotted line, respectively. Peaks at 983 and 1065 cm^{-1} correspond to SO_4^{2-} and CO_3^{2-} in solution, respectively, as they are always appeared once the electrolyte contains Na_2SO_4 (pH 12). Peak at 760 cm^{-1} is probably related to adsorbed OH^- on Cu or Cu oxide features, as it always appears regardless of electrolyte.

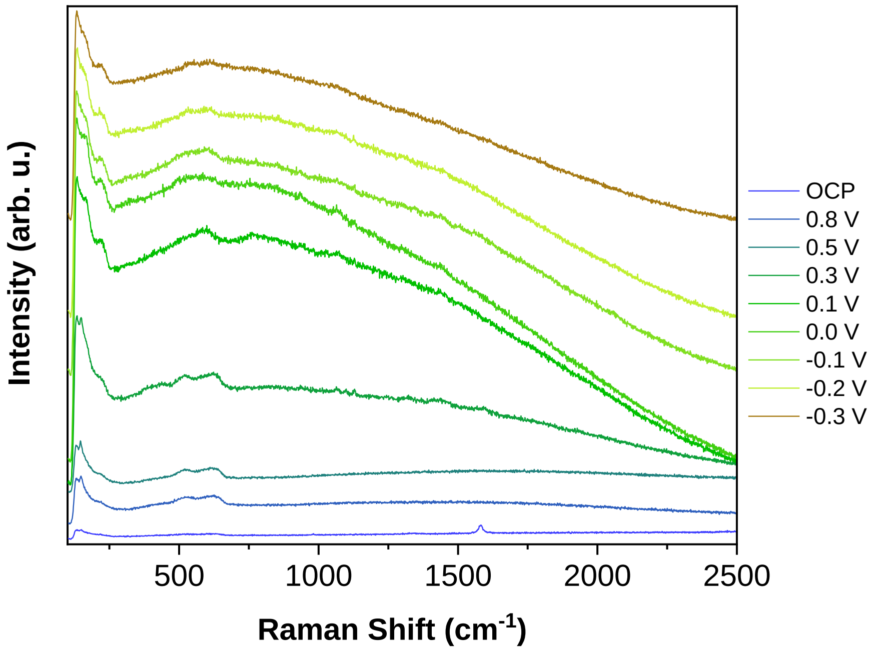

Figure S53. *In situ* Raman spectra of Cu_2O NCs under hydrogen evolution condition. Electrolyte: pH 12, 0.1 M Na_2SO_4. The applied potentials are ranging from the OCP to −0.3 V_{RHE}.

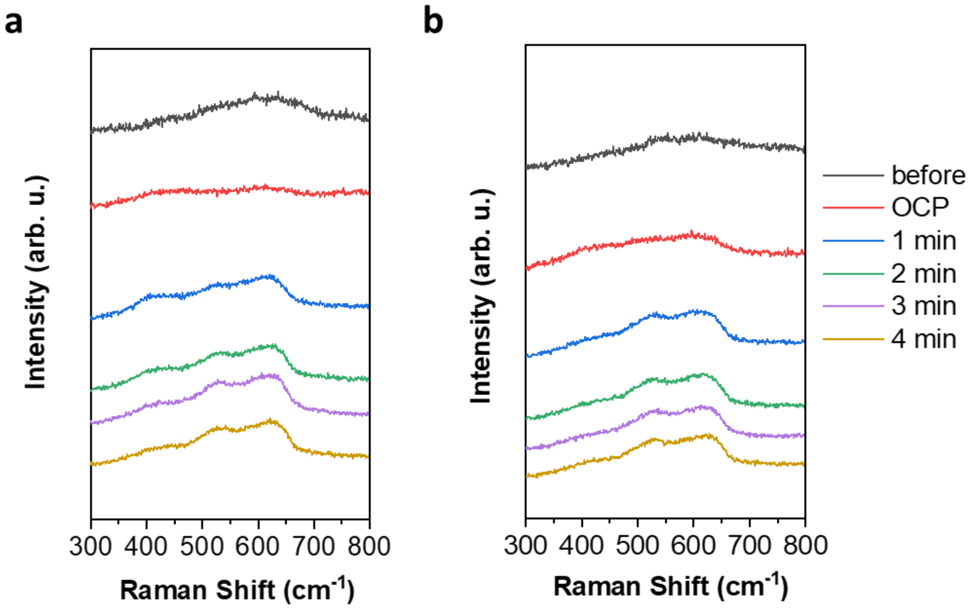

Figure S54. *In situ* Raman spectra of Cu_2O NCs after adding nitrate in alkaline sodium sulphate electrolyte. (a) The initial electrolyte is pH 12, 0.1 M Na_2SO_4. 'before' spectrum is collected with applying -0.3 V_{RHE} for 15 min. 'OCP' spectrum is collected after adding nitrate (to maintain an 8 mM electrolyte) and immediately removing the applied potential. Then the spectra are recorded every 1 min until after removing the applied potential for 4 min. (b) Control experiment with the same procedure as (a) but without adding nitrate.

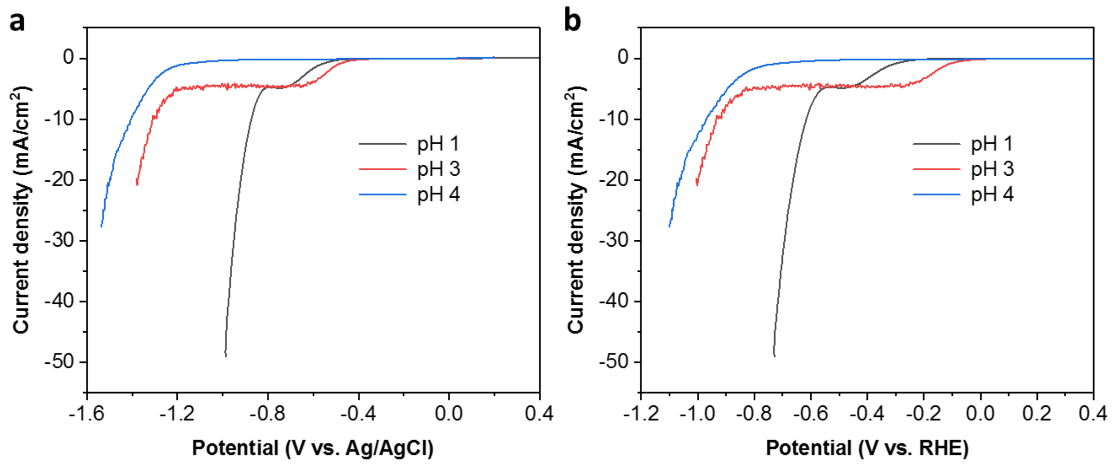

Figure S55. LSVs of Cu_2O NCs in acidic conditions (pH 1, 3 and 4) versus (a) SHE or Ag/AgCl and (b) RHE. For pH 1, the electrolyte is 0.05 M H_2SO_4 + 0.05 M Na_2SO_4 + 8 mM $NaNO_3$. For pH 3 and 4, the electrolyte is 0.1 M Na_2SO_4 + 8 mM $NaNO_3$, then the pH values are tuned by adding 0.5 M H_2SO_4.

Table S1. Quasi *in situ* XPS composition determined from the integrated area of the linear combination fittings with the respective reference spectra for Cu and its oxides.

**Sample**	**% Cu**	**% Cu_2O**	**% CuO**
As-prepared	0	72	28
NO_3RR 0.1 V_{RHE}	30	70	0
NO_3RR -0.3 V_{RHE}	92	8	0
NO_2RR 0.1 V_{RHE}	38	62	0
NO_2RR -0.3 V_{RHE}	84	16	0

Supplementary Note 1. Electrocatalytic performance in neutral electrolyte and the variation of pH values of the electrolyte solution

In this study, we used sodium sulfate (Na_2SO_4) as the supporting electrolyte for all the electrochemical measurements. We have several reasons for using Na_2SO_4 as the supporting electrolyte. First, SO_4^{2-} has the highest ionic conductivity compared to various anions, except OH^-.[3] The solubility of sulfate anions is also very high.[3] Second, SO_4^{2-} is very stable in a wide range of electrochemical window. Third, SO_4^{2-} has relatively weak adsorption on the electrode surface, making less competitive adsorption with nitrate/nitrite.[4] The latter two reasons ensure stable production of ammonia with high selectivity. As many previous literature reports, we first investigated the performance of the electrochemical nitrate/nitrite reduction in Na_2SO_4 solution, with an initial pH value close to 7. The catalyst is activated by electrochemical cycling in the presence of NO_3^-/NO_2^-, as evidenced by an increased current density observed during five consecutive LSV scans (**Figures S2a-c**). After the electrochemical treatment, the catalyst shows a dramatically higher current density and a positive shift of the onset potential in the presence of nitrate and nitrite (**Figure S2**). The voltammetric features shown in close-to-neutral pH electrolyte are similar to those observed at pH 12 (**Figure 1b, main text**).

A peculiar current increase is observed in the initial stage of NO_3RR (**Figure S3**). This behavior can be attributed to sluggish reduction of the poorly conductive Cu_2O to metallic Cu(0), which may be accompanied by concomitant structural and morphological changes.[5-6] Similar reductive electrochemical pre-treatment in the presence of nitrate was previously reported to enhance the NO_3RR performances of CuO_x catalysts.[7-10]

Selectivity data of NO_3RR/NO_2RR catalyzed by Cu_2O NCs are shown in **Figures S4-S6** and **S8-S10**). At mild cathodic potentials, NO_3RR catalyzed by Cu_2O NCs results in a selective NO_3^- conversion into NO_2^-, with a maximum FE of 73±7 % at -0.76 $V_{Ag/AgCl}$. However, NH_3 progressively increases with overpotential and becomes the predominant product at the expense of NO_2^-, with a FE_{NH3} > 80 % between -1.16 to -1.26 $V_{Ag/AgCl}$. In particular, a maximum FE of 85±2 % and a molar yield rate of 60±16 μmol h^{-1} cm^{-2}, respectively, were obtained for ammonia formation at -1.26 $V_{Ag/AgCl}$, whereas the FE for NO_2^- production is below 10% (**Figures S4a and S5a-b**). Notably, the FE_{NH3} at -1.21 $V_{Ag/AgCl}$ also gradually increases with reaction time, reaching a maximum value of > 80% after 1h electrolysis (**Figure S6**), consistent with gradual activation of the catalyst and formation of metallic Cu (**Figures S2a-c**).

The electrocatalytic performance of Cu_2O NCs was also explored in the presence of an equimolar amount of NO_2^- (**Figure S4b and S5c-d**). The catalyst exhibits more than 90% FE for NH_3 formation in a wide range of applied potentials, from -1.06 V to -1.26 $V_{Ag/AgCl}$ (**Figure S4b**). The higher FE_{NH3} of NO_2RR compared to those of NO_3RR suggests faster kinetics for NO_2^- activation and reduction by Cu_2O NCs.

A series of control experiments further confirm the key role of Cu_2O NCs in catalyzing NO_3RR to NH_3. As expected, neither detectable NH_3 nor NO_2^- amounts are observed during a potentiostatic test of Cu_2O NCs without adding $NO_3^–/NO_2^–$. Moreover, the lack of Cu_2O NCs leads to negligible current densities for NO_3RR at potentials more positive than –1.30 $V_{Ag/AgCl}$, indicating that the carbon paper support material is inert towards NO_3RR in the selected potential window (**Figure S7**). Furthermore, no significant changes in selectivity are observed as a function of the Cu_2O NCs mass loading on the electrode (0.01-0.24 mg cm^{-2}, **Figure S8**).

Quantitative ^1H-NMR analysis was carried out to validate the accuracy of the colorimetric data for NH_3 detection (**Figures S11-S13**). The integration of the typical triplet signal centered at δ = 6.84 ppm representative of $^{14}NH_4^+$ after NO_3RR and NO_2RR at -1.21 $V_{Ag/AgCl}$, respectively, results in analogous efficiencies and yield rates for ammonia production as those obtained from colorimetric analysis (**Figure S12**).[11-12] Similar results were obtained at different applied potentials as well (**Figure S13**). Furthermore, isotope labelling experiments performed by using 8 mM $Na^{15}NO_3$ as reactant and nitrogen source confirm that the detected NH_3/NH_4^+ originates from $NO_3^–$ reduction. The FE(NH_3) calculated by integration of the typical $^{15}NH_4^+$ doublet ^1H-NMR signal is consistent with the spectrophotometric quantification and the quantitative data in the presence of $Na^{14}NO_3$ (**Figure S12**). Consistent quantification results are also obtained using $Na^{14}NO_2$ or $Na^{15}NO_2$ for NO_2RR (**Figure S12**).

In these unbuffered close-to-neutral pH conditions, the pH changes during NO_3RR to higher than 11 after a 2 h catalytic test at -1.21 $V_{Ag/AgCl}$. The pH value even changes to around 10 only after five continuous CVs (20 mV/s scan rate) from -0.35 to -1.45 $V_{Ag/AgCl}$. Similar phenomena were also observed in the case of NO_2RR. **Since the pH value is continuously changing during the electrochemical measurements, we reported all the potential versus the reference electrode (Ag/AgCl), instead of versus the reversible hydrogen electrode (RHE), when using the initial neutral Na_2SO_4 solution.** Although the anion exchange membrane is not functioning well in near neutral condition, we noted that the pH value varied from close to 7 to 10.7 in a single compartment cell for 2h electrolysis (with stirring) in 0.1 M Na_2SO_4 + 8 mM $NaNO_3$ at -1.21 $V_{Ag/AgCl}$. The pH change is mainly due to continuous consumption of protons and generation of ammonia. Ammonia can be hydrolyzed to ammonium and then generating hydroxyl ions.

The increment of pH values can be much alleviated when the initial pH is higher than 11.5. The pH variation due to the generation of ammonia is gradually negligible in an electrolyte with higher pH values. The anion exchange membrane also works well in alkaline condition. For examples, the pH value of the electrolyte (0.1 M Na_2SO_4 + 8 mM $NaNO_3$, initial pH 12.0) changes from 12.0 to 12.2 after 2 h electrolysis at -0.30 V_{RHE} (-1.20 $V_{Ag/AgCl}$). The pH values keep constant in 0.1 M NaOH + 8 mM $NaNO_3$ electrolyte (pH 12.9) after 2 h electrolysis at -0.30 V_{RHE} (-1.26 $V_{Ag/AgCl}$). Noted that during the experiments of electrokinetic analysis (including linear sweep voltammetries, steady state current-potential plots, scan rate dependence studies, and rotating disk electrode experiments), the pH values

of the bulk solution can be largely maintained (maximum 0.2 variation for pH 11.5; less than 0.1 variation for pH 12.0 and 12.5; no change for pH 12.9). Therefore, we chose to study the pH effects, reaction kinetics and mechanisms in the pH range from 11.5 to 12.9. Likewise, we reported the potential versus RHE in this pH range.

Although the NO_3RR selectivity trend is similar for all the explored conditions, alkaline conditions were found to promote the FE(NH_3) at lower applied overpotential. For example, in pH 12.9, at 0.0 V_{RHE} and -0.1 V_{RHE}, FE(NH_3) are 31±8 % and 67±1 %, respectively (**Figure S15a**). The FE(NH_3) are 25±1 % and 50±7 % for the corresponding potential in pH 12.0, respectively (**Figure 1c, main text**). For initial neutral pH electrolyte, if we consider the 'real' pH as 11.0 (pH > 11 after 2h electrolysis at -1.21 $V_{Ag/AgCl}$), the FE(NH_3) are 10±4 % at -0.86 $V_{Ag/AgCl}$ (-0.01 V_{RHE} for pH 11) and 48±3 % at -0.96 $V_{Ag/AgCl}$ (-0.11 V_{RHE} for pH 11), respectively (**Figure S4a**). Although this assumption is not strictly rigorous since a positive applied potential resulting smaller degree of pH change, the trend implies that the FE(NH_3) is improved when the electrolyte pH is increased. The data is consistent with *operando* XAS data that faster reduction of Cu is observed in electrolyte with higher pH (**Figure S23**).

Supplementary Note 2. Electrokinetics in low current region and quasi Langmuir model

For any electrochemical reaction, *yR → xO + ne*$^-$, according to Butler-Volmer equation, the forward and backward reaction rate can be described as below:

$$v_f = k^0 \exp\left(\frac{\beta nF(E-E^{\Theta\prime})}{RT}\right) C_R^y \qquad \text{(S1)}$$

$$v_b = k^0 \exp\left(-\frac{(1-\beta)nF(E-E^{\Theta\prime})}{RT}\right) C_O^x \qquad \text{(S2)}$$

F is the Faraday constant (96485 C/mol), *R* is the universal gas constant (8.314 J/K/mol), *T* is the thermodynamic temperature of the reaction. C_R represents the concentration of reactant and the *y* is its reaction order. C_O represents the concentration of the product and the *x* is its reaction order. *n* is the transferred electron numbers. β is the transfer coefficient or symmetric factor of the anodic reaction. *E* is the applied potential and $E^{\Theta\prime}$ is the formal potential, which is the reference point. When the applied potential $E = E^{\Theta\prime}$, $C_{R*}^y = C_{O*}^x$ (the concentration of reactant and product at this point is C_{R*} and C_{O*}, respectively) and the forward reaction and backward reactions have the same reaction rate (Eq. S3). k^0 is the standard rate constant, which has the form of Arrhenius equation and is the same for forward and backward reactions. The detailed derivation of these equations are described in the book "Electrochemical Methods: Fundamental and Applications" by A. J. Bard and L. R. Faulkner.[13]

$$v_f = v_b = k^0 C_{R*}^y = k^0 C_{O*}^x \qquad \text{(S3)}$$

In the case of cathodic reaction is highly irreversible, on the basis of Eq. S2, the current density can be expressed as:

$$j = -nFC_O^x k^0 exp\left(-\frac{n\alpha F(E-E^{\Theta\prime})}{RT}\right) \qquad \text{(S4)}$$

The minus sign indicates the reductive reaction. *α* is the transfer coefficient of the cathodic reaction (*α = 1-β*). We assume the coverage of key intermediates during nitrate/nitrite reduction is less than 5% and the pre-exponential rate constant k^0 remains unchanged in the same reaction temperature. Such assumptions could be fulfilled when the reaction rate is not high (the applied overpotential is moderate), and in this case the reaction kinetics can be described by quasi-equilibrium Langmuir isotherm adsorption model.[14-17] Noted that the assumption is only reasonable in the absence of diffusion limitation (or at least the diffusion limitation can be ignored). Generally, the key process of nitrate/nitrite reduction involves a pre-equilibrium (quasi-equilibrium) step (PES) and a rate-determining step (RDS).[15-17] The reactions can be depicted as below.

$$A + n_1e^- + n_2H^+ \underset{}{\overset{k_1^0}{\rightleftharpoons}} B$$

$$B + n_3e^- + n_4H^+ \xrightarrow{k_2^0} C$$

The PES can either be a single electrochemical step (in most case $n_1 = 1$) or combined consecutive electrochemical steps ($n_1 \geq 1$). For RDS, the transferred electrons n_3 can be either 1 or 0. k_1^0 are the standard rate constant of the PES. k_2^0 is the standard rate constants of RDS. The steady-state velocity can be expressed as follows:

$$v = k_2^0\theta_B[H^+]^{n_4}\exp\left(-\frac{n_3\alpha_2F\left(E-E_2^{\theta'}\right)}{RT}\right) \qquad \text{(S5)}$$

α_2 is the transfer coefficient of RDS, $E_2^{\theta'}$ is the formal potential of RDS, θ_B represents the partial surface coverage of intermediate *B*. θ_B can be defined in terms of the surface coverage of the resting state *A* (θ_A). The relationship between θ_B and θ_A can be deduced from the equilibrium equation:

$$v_1 = k_1^0\theta_A[H^+]^{n_2}\exp\left(-\frac{n_1\alpha_1F\left(E-E_1^{\theta'}\right)}{RT}\right) \qquad \text{(S6)}$$

$$v_{-1} = k_1^0\theta_B\exp\left(\frac{n_1(1-\alpha_1)F\left(E-E_1^{\theta'}\right)}{RT}\right) \qquad \text{(S7)}$$

$$\theta_A + \theta_B = 1 \qquad \text{(S8)}$$

For the quasi-equilibrium condition:

$$v_1 = v_{-1} \qquad \text{(S9)}$$

Then we have:

$$\theta_B = \frac{[H^+]^{n_2}\exp\left(-\frac{n_1F\left(E-E_1^{\theta'}\right)}{RT}\right)}{1+[H^+]^{n_2}\exp\left(-\frac{n_1F\left(E-E_1^{\theta'}\right)}{RT}\right)} \qquad \text{(S10)}$$

Substituting the expression of θ_B (Eq. S10) for Eq. S5, the steady-state velocity can be expressed as:

$$v = \frac{k_2^0[H^+]^{n_2+n_4}\exp\left(-\frac{(n_1+n_3\alpha_2)EF}{RT}\right)\exp\left(\frac{n_1FE_1^{\theta'}+n_3\alpha_2FE_2^{\theta'}}{RT}\right)}{1+[H^+]^{n_2}\exp\left(-\frac{n_1F\left(E-E_1^{\theta'}\right)}{RT}\right)} \qquad \text{(S11)}$$

If Langmuir conditions are assumed, the surface coverage of B (θ_B) should be less than 5%, then we have ($[H^+]^{n_2}\exp\left(-\frac{n_1F\left(E-E_1^{\theta'}\right)}{RT}\right) << 1$):

$$\theta_B = \frac{[H^+]^{n_2}\exp\left(-\frac{n_1F\left(E-E_1^{\theta'}\right)}{RT}\right)}{1+[H^+]^{n_2}\exp\left(-\frac{n_1F\left(E-E_1^{\theta'}\right)}{RT}\right)} \approx [H^+]^{n_2}\exp\left(-\frac{n_1F\left(E-E_1^{\theta'}\right)}{RT}\right) \qquad \text{(S12)}$$

$$v = k_2^0[H^+]^{n_2+n_4}\exp\left(-\frac{(n_1+n_3\alpha_2)EF}{RT}\right)\exp\left(\frac{n_1FE_1^{\Theta\prime}+n_3\alpha_2FE_2^{\Theta\prime}}{RT}\right) \quad \text{(S13)}$$

$$j = -8Fv = k^0[H^+]^{n_2+n_4}\exp\left(-\frac{(n_1+n_3\alpha_2)EF}{RT}\right) \quad \text{(S14)}$$

$$k_0 = -8Fk_2^0\exp\left(\frac{n_1FE_1^{\Theta\prime}+n_3\alpha_2FE_2^{\Theta\prime}}{RT}\right) \quad \text{(S15)}$$

k_0 is the overall rate constant. The formal potential is independent of the concentration of reactants/products. The overall transferred electron numbers are 8 for the reduction of nitrate to ammonia, 6 for the reduction of nitrite to ammonia, and 2 for the reduction of nitrate to nitrite, respectively.

The Tafel slope, $\left(\frac{\partial E}{\partial \log j}\right)_{pH}$ or $\left(\frac{\partial \eta}{\partial \log j}\right)_{pH}$, can be expressed as:

$$\left(\frac{\partial E}{\partial \log j}\right)_{pH} = \left(\frac{\partial \eta}{\partial \log j}\right)_{pH} = \frac{2.303RT}{(n_1+n_3\alpha_2)F} \quad \text{(S16)}$$

If the first generation of B is the RDS (there is no pre-equilibrium step):

$$\mathbf{A + n_1e^- + n_2H^+ \xrightarrow{k_1^0} B}$$

Then we have:

$$v = k_1^0\theta_A[H^+]^{n_2}\exp\left(-\frac{n_1\alpha_1F\left(E-E_1^{\Theta\prime}\right)}{RT}\right) \quad \text{(S17)}$$

$$j = -8Fv = -8Fk_1^0\theta_A[H^+]^{n_2}\exp\left(-\frac{n_1\alpha_1F\left(E-E_1^{\Theta\prime}\right)}{RT}\right) \quad \text{(S18)}$$

Thus, the Tafel slope is:

$$\left(\frac{\partial E}{\partial \log j}\right)_{pH} = \left(\frac{\partial \eta}{\partial \log j}\right)_{pH} = \frac{2.303RT}{n_1\alpha_1F} \quad \text{(S19)}$$

Typically, the elemental electrochemical step involves one electron transfer; therefore, if the first electrochemical activation of the reactant is the RDS, we have close to 120 mV/dec Tafel slope in standard condition (298 K, 1 atm), when the transfer coefficient is 0.5. In this research work, the nitrate reduction in pH from 11.5 to 12.9 fits well with this condition, considering reasonable change of the transfer coefficient (0.3-0.7) within the variation of pH values.

Likewise, when the first step is PES and the second step is RDS, we have close to 40 mV/dec Tafel slope, assuming the transfer coefficient as 0.5. If the second step does not involve direct electron transfer (e.g. chemical hydrogenation step via adsorbed hydrogen), then the Tafel slope is 59 mV/dec, as n_3 is equal to 0. In this research work, the nitrite reduction in high pH (12.5, 12.9) fits well with this condition (see **Supplementary Note 4** for more details).

Supplementary Note 3. Electrokinetics in high current region: scan rate dependence study and rotating disk electrode (RDE) experiments

The electrokinetics in the low current region, where the catalytic current is less influenced by diffusion limitations, can be studied using a quasi-equilibrium Langmuir adsorption model. In the case of high current region, the performance is limited by diffusion and mass transport of reactant. In this case, the Tafel slope analysis is no longer working. Therefore, the electrochemical methods considering diffusion control should be adopted. Here, we studied the scan rate dependence of current-potential relationship and used rotating disk electrodes to investigate the kinetic behavior of catalyst in the range of diffusion control.

The voltammetric behavior (current-potential relationship) of a certain reaction, $O + ne^- \rightarrow R$, can typically be described as Eq. S20-S23.[13]

$$i = nFAC_o(\pi D_o \sigma)^{0.5}\chi(\sigma t) \qquad \text{(S20)}$$

$$i = nFAC_o(\pi D_o b)^{0.5}\chi(bt) \qquad \text{(S21)}$$

$$\sigma = \frac{nF}{RT}v \qquad \text{(S22)}$$

$$b = \frac{\alpha_r n_r F}{RT}v \qquad \text{(S23)}$$

Eqs. S20 and S22 are used for reversible electrochemical systems (the conversion between O and R is reversible); while Eqs. S21 and S23 are used for irreversible electrochemical reactions. In these equations, i is the measured peak current (unit: A). n is the number of total transferred electrons, n_r *and* α_r is the number of transferred electrons and transfer coefficient of the RDS, respectively. F is the Faraday constant (96485 C/mol), R is the universal gas constant (8.314 J/K/mol), T is the thermodynamic temperature of the reaction, A is electrode area (unit: cm^2). C_o and D_o are concentration (unit: mol cm^{-3}) and diffusion coefficient (unit: cm^2 s^{-1}) of reactant, respectively. v is the scan rate (unit: V s^{-1}). $\chi(\sigma t)$ and $\chi(bt)$ are dimensionless current function of charge transfer for reversible and irreversible electrochemical system.[13] The maximum value of $\chi(\sigma t)$ is 0.4463, while the maximum value of $\chi(bt)$ is 0.496. Therefore, at 298 K, Eq. S20 and Eq. S21 can be rearranged as Eq. S24 and S25.[13, 18]

$$i = (2.69 \times 10^5)n^{3/2}AD_0^{1/2}C_o v^{1/2} \qquad \text{(S24)}$$

$$i = (2.99 \times 10^5)(\alpha_r n_r)^{1/2}nAD_0^{1/2}C_o v^{1/2} \qquad \text{(S25)}$$

Eq. S24 is known as the Randles–Sevcik equation, which describes the behavior of reversible or quasi-reversible redox peaks. Eq. S25 is called as Nicholson–Shain equation, which describes the behavior of irreversible catalytic peaks. In both conditions, the current has linear dependence on the square root of scan

rate. Due to the reversible feature, for Eq. S24, both the anodic and cathodic current should fit this equation.

Noted that in Eq. S25, there are two different numbers of transferred electrons: the number of transferred electrons of the RDS, n_r; and the total number of transferred electrons for the whole reaction, n. The $\alpha_r n_r$ (noted that typically n_r should be 1 for most of electrochemical reaction) can be determined by using the equation below.[18]

$$\left|\frac{dE_p}{dln\sqrt{v}}\right| = \frac{RT}{F\alpha_r n_r} \quad \text{(S26)}$$

$$\left|\frac{dE_p}{dlog\, v}\right| = \frac{2.303RT}{2Fn_r\alpha_r} = \frac{29.6\ \mathrm{mV}}{n_r\alpha_r} \quad \text{(S27)}$$

Therefore, the catalytic peak potential is linearly shifted with the logarithm of the scan rate (here the unit of scan rate is mV s^{-1}). From the slope of fitted E_p-*log v* plots, the $\alpha_r n_r$ can be determined, and then Eq. S25 can be used for calculating the transferred electrons for different catalytic peaks by using the derived slope of i-$v^{1/2}$ plots.

In addition to the Nicholson-Shain equation, the transferred electrons at a certain potential can also be determined from Koutecký–Levich equation (Eq. 28) using RDE.[13, 19]

$$\frac{1}{j} = \frac{1}{j_k} + \frac{1}{0.62nFD_0^{2/3}\omega^{1/2}v^{-1/6}c_0} \quad \text{(S28)}$$

In Eq. 28, j is the overall apparent catalytic current density (unit: mA cm^{-2}), j_k is the kinetic current density, ω is the rotating rate of the electrode (unit: rad/s), v is the kinematic viscosity of the electrolyte (unit: cm^2 s^{-1}). By gradually changing the rotating rate, one can obtain the $1/j$-$1/\omega^{1/2}$ plot. According to the slope of linearly fitted $1/j$-$1/\omega^{1/2}$ plot, the transferred electrons n can be acquired.

Supplementary Note 4. Proposed reaction mechanisms of Cu_2O NCs for NO_3RR and NO_2RR

Combining the results of various *in situ/operando* characterizations and the electrochemical kinetic studies, the catalytic process of NO_3RR and NO_2RR can be proposed as below. The RDS of nitrate reduction is the first proton-coupled electron transfer (PCET) activation of nitrate. This assumption is based on closed to 120 mV/dec Tafel slope and first-order rate dependence on proton concentration. Based on **Figure S30**, Eq. S16 and S19, the transfer coefficient *α* ranges from 0.33 to 0.58, depending on the pH values of electrolyte (from 11.5 to 12.9), which is within the normal range of variation (0.3-0.7). Therefore, we thought the RDS should remain the same in an electrolyte with pH ranging from 11.5 to 12.9. The most possible adsorbed intermediate generated after one proton one electron activation of nitrate is $*NO_2OH^-$, in which one oxygen is added with one hydrogen (4.2).[20-21] $*NO_2OH^-$ is further reduced to $*NO_2^-$ via another PCET process (4.3).

$$NO_3^- + * \xrightarrow{K_{NO_3^-}} *NO_3^- \quad (4.1)$$

$$*NO_3^- + H^+ + e^- \xrightarrow{k_{4.2}^0} *NO_2OH^- \quad (4.2)$$

$$*NO_2OH^- + H^+ + e^- \xrightarrow{k_{4.3}^0} *NO_2^- + H_2O \quad (4.3)$$

(4.1) is the adsorption equilibrium reaction of nitrate. $K_{NO_3^-}$ is the adsorption equilibrium constant of nitrate. The surface concentration of adsorbed NO_3^- is proportional to NO_3^- concentration in solution. θ_x is the coverage of other competitive adsorbed species, mainly like SO_4^{2-} and H_2O/OH^-, etc, which can be considered as constant in a certain degree.

$$\theta_{NO_3^-} = K_{NO_3^-}[NO_3^-](1 - \theta_{NO_3^-} - \theta_x) \quad \text{(S29)}$$

The reaction rate of nitrate reduction is:

$$v = k_{4.2}^0 \theta_{NO_3^-}[H^+] \exp\left(-\frac{\alpha_{4.2}F\left(E - E_{4.2}^{\Theta'}\right)}{RT}\right) \quad \text{(S30)}$$

$k_{4.2}^0$ and $E_{4.2}^{\Theta'}$ are standard rate constant and formal potential of step 4.2, respectively. $\alpha_{4.2}$ is the transfer coefficient.

The reaction order on both $\theta_{NO_3^-}$ and $[H^+]$ are 1. To determine the reaction order on $[NO_3^-]$, we have:

$$\left(\frac{\partial \log v}{\partial \log \theta_{NO_3^-}}\right)_{pH} = \left(\frac{\partial \log v}{\partial \log [NO_3^-]}\right)_{pH}\left(\frac{\partial \log [NO_3^-]}{\partial \log \theta_{NO_3^-}}\right)_{pH} = 1 \quad \text{(S31)}$$

$$\left(\frac{\partial \log \theta_{NO_3^-}}{\partial \log [NO_3^-]}\right)_{pH} = \left(\frac{\partial \theta_{NO_3^-}}{\partial [NO_3^-]}\right)_{pH} \times \frac{[NO_3^-]}{\theta_{NO_3^-}} \quad \text{(S32)}$$

From Eq. S29,

$$\left(\frac{\partial\theta_{NO_3^-}}{\partial[NO_3^-]}\right)_{pH} = \frac{K_{NO_3^-}(1-\theta_x)}{(1+K_{NO_3^-}[NO_3^-])^2} \quad \text{(S33)}$$

$$\left(\frac{\partial \log v}{\partial \log\ [NO_3^-]}\right)_{pH} = \left(\frac{\partial \log\ \theta_{NO_3^-}}{\partial \log\ [NO_3^-]}\right)_{pH} = \frac{1-\theta_x-\ \theta_{NO_3^-}}{1-\theta_x} \quad \text{(S34)}$$

Therefore, the reaction order on nitrate concentration is decreased with increasing nitrate adsorption coverage. According to experimental data from **Figure S32**, we indeed observed a fractional reaction order (less than 1) on nitrate concentration.

$$*NO_3^- + H_2O + e^- \xrightarrow{k_{4.4}^0} *NO_2OH^- + OH^- \quad (4.4)$$

If the step 4.2 serves as the RDS, the reaction has a first-order dependence on proton concentration (Eq. S30). Conversely, when the water is the hydrogen source, the reaction should have zero order dependence on proton concentration (Step 4.4 and Eq. S35). $k_{4.4}^0$ and $E_{4.4}^{\theta'}$ are standard rate constant and formal potential of step 4.4, respectively. $\alpha_{4.4}$ is the transfer coefficient.

$$v = k_{4.4}^0\theta_{NO_3^-}\exp\left(-\frac{\alpha_{4.4}F\left(E-E_{4.4}^{\theta'}\right)}{RT}\right) \quad \text{(S35)}$$

We measured the order of proton as 0.70 ± 0.12, exhibiting a deviation from unity. Additionally, our investigation revealed a decrease in the proton order with overpotential, ranging from -0.82 to -0.58 $V_{Ag/AgCl}$, leading to a considerable uncertainty regarding the order value. One possible explanation for this deviation is that the RDS may not exclusively involve H^+/e^-; water could also serve as a source of hydrogen, especially given the elevated pH. Moreover, the acceleration of water dissociation at more negative potentials contributes to this deviation. Nevertheless, considering the proximity of the order to 1 rather than 0, we deduced that protons remain the primary source of hydrogen.

In the case of NO_2RR, the Tafel slope values fit transfer coefficient values as 0.60 and 0.72, when electrolyte pH values are 11.5 and 12.0, respectively (**Figure S41**). The reaction order on proton concentration is also one. Hence, in this pH range, RDS should be PCET activation of nitrite (4.6), similar as the case of NO_3RR. The possible product of this reaction is $*NOOH^-$.[20-21] $*NOOH^-$ is further decomposed to *NO in the presence of proton (4.7).

$$NO_2^- + * \xrightarrow{K_{NO_2^-}} *NO_2^- \quad (4.5)$$

$$*NO_2^- + H^+ + e^- \xrightarrow{k_{4.6}^0} *NOOH^- \quad (4.6)$$

$$*NOOH^- + H^+ \xrightarrow{k_{4.7}^0} *NO + H_2O \quad (4.7)$$

(4.5) is the adsorption equilibrium reaction of nitrite. $K_{NO_2^-}$ is the adsorption equilibrium constant of nitrite. Similar as Eq. S29 and S30, we have:

$$\theta_{NO_2^-} = K_{NO_2^-}[NO_2^-](1 - \theta_{NO_2^-} - \theta_x) \qquad \text{(S36)}$$

$$v = k_{4.6}^0 \theta_{NO_2^-}[H^+] \exp\left(-\frac{\alpha_{4.6}F\left(E - E_{4.6}^{\Theta'}\right)}{RT}\right) \qquad \text{(S37)}$$

$k_{4.6}^0$ and $E_{4.6}^{\Theta'}$ are standard rate constant and formal potential of step 4.6, respectively. $\alpha_{4.6}$ is the transfer coefficient.

Similar as Eq. S31-Eq.S34, we deduced that:

$$\left(\frac{\partial \log v}{\partial \log\ [NO_2^-]}\right)_{pH} = \frac{1-\theta_x - \theta_{NO_2^-}}{1-\theta_x} \qquad \text{(S38)}$$

When further increasing the pH value, the RDS is less possible to be first PCET activation of nitrite, since the transfer coefficient is higher than 0.8, or even more than 1 for pH 12.9, according to Eq. S16 and S19. We noticed that the Tafel slope in pH 12.5 and 12.9 is close to 59 mV/dec (**Figure S41**), which means a chemical step without apparent electron transfer is the RDS (**Supplementary Note 2**). Moreover, there is a one-electron transfer PES prior to the RDS. As water dissociation is more favorable in alkaline condition with higher pH[22], we consider that the PES should be water dissociation that produce adsorbed hydrogen *H (4.8). A subsequent hydrogenation of NO_2^- via *H is the RDS (4.9).

$$^*H_2O + e^- \xrightarrow{k_{4.8}^0} {}^*H + OH^- \qquad (4.8)$$

$$^*NO_2^- + {}^*H \xrightarrow{k_{4.9}^0} {}^*NOOH^- \qquad (4.9)$$

In this case, the reaction rate of NO_2RR can be described as below.

$$v = k_{4.9}^0 \theta_{NO_2^-} \theta_H \qquad \text{(S39)}$$

$k_{4.8}^0$ and $k_{4.9}^0$ are the standard rate constants of steps 4.8 and 4.9, respectively. $\theta_{NO_2^-}$ and θ_H represent the surface coverage of adsorbed NO_2^- and hydrogen. The step 4.8 is in quasi-equilibrium, and then we have Eq. S40.

$$k_{4.8}^0 \theta_{H_2O} \exp\left(-\frac{\alpha_{4.8}F\left(E - E_{4.8}^{\Theta'}\right)}{RT}\right) = k_{4.8}^0 \theta_H K_w/[H^+] \exp\left(\frac{(1-\alpha_{4.8})F\left(E - E_{4.8}^{\Theta'}\right)}{RT}\right) \qquad \text{(S40)}$$

θ_{H_2O} is the the surface concentration of adsorbed water. K_w is the water dissociation constant (10^{-14}). $\alpha_{4.8}$ is the transfer coefficient of the forward reaction. Combining Eq. S39-S40, we have the steady-state velocity expressed as below.

$$v = (k_{4.9}^0 \theta_{H_2O} \theta_{NO_2^-}/K_w)[H^+] \exp\left(-\frac{\left(E - E_{4.8}^{\Theta'}\right)F}{RT}\right) \qquad \text{(S41)}$$

Hence, the reaction has first order dependence on proton concentration and shows a Tafel slope of 59 mV/dec, which is consistent with experimental data.

We noticed that the onset potential of NO_2RR is more negative and more closed to the hydrogen evolution onset, in comparison to NO_3RR using the same catalyst. Therefore, it is possible that water dissociation that produces adsorbed hydrogen (*H) happens in NO_2RR rather than in nitrate reduction to nitrite. However, the potential region for NO_2RR is still quite positive compared to the HER onset. Therefore, the competitive adsorption between *NO_2^- and *H_2O (or *H), can be regarded as negligible, which also means θ_{H_2O} remains as constant and independent of *NO_2^- under Langmuir condition. Similarly, we can deduce that the reaction order of NO_2^- is the same as described in Eq. S38. Indeed, we also observed a fractional reaction order (less than 1) on nitrite concentration as depicted in **Figure S43**.

From the data of scan rate dependence catalytic current and RDE experiments, we identified that in the potential region where ammonia is the main product of NO_3RR, the total electron transfer number is 6, or three times of that at the potential region where NO_2^- is the main product. Moreover, the total electron transfer number of NO_2RR is 4. These results implied that ammonia production undergoes a first 6-electron reduction of nitrate (or 4-electron reduction of nitrite), following by another 2-electron transfer. The most probable products of 6-electron reduction of nitrate is hydroxylamine (NH_2OH). Since NH_2OH is not a stable species under highly negative potential, it can be further reduced to ammonia rapidly.[7, 23] The quasi-infinite Tafel slope of NH_2OH electroreduction indicates the NH_2OH is converted to NH_3 via chemical steps without apparent interfacial electron transfer on metallic Cu surface. Since we observed quasi-reversible *OH desorption/adsorption peaks after the peaks related to catalytic NO_3^-/NO_2^- to NH_2OH, we proposed that the chemical dissociation of *NH_2OH to *NH_2 and *OH is the RDS.

$$^*NH_2OH + {}^* \xrightarrow{k_{4.10}^0} {}^*NH_2 + {}^*OH \qquad (4.10)$$

$$^*NH_2 + H_2O \xrightarrow{k_{4.11}^0} NH_3 + {}^*OH \qquad (4.11)$$

$$^*OH + e^- \xrightarrow{k_{4.12}^0} OH^- \qquad (4.12)$$

$$v = k_{4.10}^0 \theta_{NH_2OH}\left(1 - \theta_{NH_2OH} - \theta_{NH_2} - \theta_{OH}\right) = k_{4.11}^0 \theta_{NH_2} \qquad \text{(S42)}$$

The reaction should be in steady state and reaction 4.10 and 4.11 have the same reaction rate. $k_{4.10}^0$ ~ $k_{4.12}^0$ are the standard rate constants of step 4.10 to 4.12. Due to the surface adsorption/desorption properties of reaction 4.12, the θ_{OH} and [OH^-] are in equilibrium.

$$k_{4.12}^0 \theta_{OH} \exp\left(-\frac{\alpha_{4.12} F\left(E - E_{4.12}^{\theta'}\right)}{RT}\right) = k_{4.12}^0 K_w/[H^+] \exp\left(\frac{(1-\alpha_{4.12}) F\left(E - E_{4.12}^{\theta'}\right)}{RT}\right) \qquad \text{(S43)}$$

$$\theta_{OH} = \exp\left(\frac{F\left(E - E_{4.12}^{\theta'}\right)}{RT}\right) K_w/[H^+] \qquad \text{(S44)}$$

Alternatively, the chemical reduction of NH_2OH could also involve the formation of a short-lived amino radical,[24] generating dissolved Cu species[25]. The detailed process is described as below.

$$Cu(0) + {}^*NH_2OH \longrightarrow Cu^+(aq) + {}^\bullet NH_2 + OH^-$$

$$ {}^\bullet NH_2 + H_2O \longrightarrow NH_3 + {}^\bullet OH$$

$$ {}^\bullet OH + Cu(0) \longrightarrow Cu^+(aq) + OH^-$$

$$Cu^+(aq) + 2NH_3 \longrightarrow Cu(NH_3)_2^+$$

Both Cu^+ and $[Cu(NH_3)_2]^+$ are not stable and can be further oxidized to Cu^{2+} and $[Cu(NH_3)_4]^{2+}$. All of these dissolved Cu species can be redeposited on the catalyst surface under reductive potential[25].

From $^*NOOH^-$, 4 protons and 3 electrons are required for producing $^*NH_2OH$. Whether H^+/e^- or *H is the reduction reagent depends on the pH values. Similar as in NO_2RR, here we proposed that *H should be dominated in electrolyte with higher pH values (benefiting from easier water dissociation in high pH). According to *in situ* Raman spectroscopy data, we proposed that $^*NOOH^-$ is sequentially reduced or protonated to *NO, *HNO (possibly also *NHOH), and finally generating $^*NH_2OH$, which is also consistent with several theoretical studies.[20-21, 26-27]

We also evaluated the performance of the Cu_2O NCs for nitrate reduction in acidic conditions (pH 1, 3, and 4) to check if the catalyst has similar reaction mechanisms in acidic and alkaline conditions. We used a cation exchange membrane instead of the anion exchange membrane in these pH conditions. We identified that the catalyst behaved substantially different with respect to several aspects in acidic condition as compared to that in alkaline conditions (**Figure S55**). (1) The linear scan voltammetry (LSV) curves are quite different for different pH values in acidic conditions. From the current density on the standard hydrogen electrode (SHE) scale (**Figure S55a**), we can find that the current density related to nitrate reduction between -0.4 and -1.2 $V_{Ag/AgCl}$ is initially significantly increased with decreasing pH (increasing proton concentration) and then decreases with increasing proton concentration. On the contrary, the hydrogen evolution related current monotonously increased or the onset shifts anodically with proton concentration. Therefore, the mechanisms do not remain the same within the pH range from pH 1 to pH 4. (2) The selectivity and the activity are much worse in acidic conditions (**Figure S55b**). At pH 3, when applying -0.62 V_{RHE}, the Faradaic Efficiency of ammonia ($FE(NH)_3$) is only 53.6%, while the $FE(NO_2^-)$ is 46.4%. At pH 4, when applying similar potential (-0.67 V_{RHE}), the $FE(NH)_3$ is 58.2% and the $FE(NO_2^-)$ is 40.8%. Although in pH 1 the $FE(NH)_3$ can reach 70.5% and the $FE(NO_2^-)$ is only 1.8% at -0.54 V_{RHE}, the bubbles were clearly observed, indicating strong hydrogen evolution background. Furthermore, the onset overpotential of acidic nitrate reduction is much more cathodic compared to that of the alkaline

nitrate reduction as well (**Figure S55b**). At least ca. -0.1 V_{RHE} is required to observe obvious catalytic current. (3) Moreover, the electrolyte pH values are increased after constant potential electrolysis due to the generation of ammonia, similar to what we have detected in neutral electrolyte. After 2h electrolysis for product quantification, significant pH change (from 4.0 to 11.5) were observed for the pH 4 electrolyte. A slight pH change happened for pH 1 (from 1.0 to 1.3) and pH 3 (from 3.0 to 3.6) conditions as well.

Based on these preliminary experimental results, we can conclude that the reaction mechanisms between acidic and alkaline NO_3RR are probably different. Rigorous and comprehensive mechanistic study for acidic NO_3RR also requires using buffered electrolyte to avoid significant pH change, which is beyond the scope of our current manuscript.

References

1. Arán-Ais, R. M.; Scholten, F.; Kunze, S.; Rizo, R.; Roldan Cuenya, B., The role of in situ generated morphological motifs and Cu(I) species in C_{2+} product selectivity during CO_2 pulsed electroreduction. *Nat. Energy* **2020,** *5* (4), 317-325.

2. Reyter, D.; Belanger, D.; Roue, L., Elaboration of Cu−Pd films by coelectrodeposition: application to nitrate electroreduction. *J. Phy. Chem. C* **2009,** *113* (1), 290-297.

3. Monteiro, M. C. O.; Philips, M. F.; Schouten, K. J. P.; Koper, M. T. M., Efficiency and selectivity of CO_2 reduction to CO on gold gas diffusion electrodes in acidic media. *Nat. Commun.* **2021,** *12* (1), 4943.

4. Islam, M.; Patel, R., Synthesis and physicochemical characterization of Zn/Al chloride layered double hydroxide and evaluation of its nitrate removal efficiency. *Desalination* **2010,** *256* (1-3), 120-128.

5. Ren, D.; Deng, Y.; Handoko, A. D.; Chen, C. S.; Malkhandi, S.; Yeo, B. S., Selective electrochemical reduction of carbon dioxide to ethylene and ethanol on copper (I) oxide catalysts. *ACS Catal.* **2015,** *5* (5), 2814-2821.

6. Ning, H.; Mao, Q.; Wang, W.; Yang, Z.; Wang, X.; Zhao, Q.; Song, Y.; Wu, M., N-doped reduced graphene oxide supported Cu_2O nanocubes as high active catalyst for CO_2 electroreduction to C_2H_4. *J. Alloys Compd.* **2019,** *785*, 7-12.

7. Wang, Y.; Zhou, W.; Jia, R.; Yu, Y.; Zhang, B., Unveiling the Activity Origin of a Copper-based Electrocatalyst for Selective Nitrate Reduction to Ammonia. *Angew. Chem. Int. Ed.* **2020,** *59* (13), 5350-5354.

8. Hu, Q.; Qin, Y.; Wang, X.; Wang, Z.; Huang, X.; Zheng, H.; Gao, K.; Yang, H.; Zhang, P.; Shao, M.; He, C., Reaction intermediate-mediated electrocatalyst synthesis favors specified facet and defect exposure for efficient nitrate–ammonia conversion. *Energy Environ. Sci.* **2021,** *14* (9), 4989-4997.

9. Xu, Y.; Wang, M.; Ren, K.; Ren, T.; Liu, M.; Wang, Z.; Li, X.; Wang, L.; Wang, H., Atomic defects in pothole-rich two-dimensional copper nanoplates triggering enhanced electrocatalytic selective nitrate-to-ammonia transformation. *J. Mater. Chem. A* **2021,** *9* (30), 16411-16417.

10. Chen, F. Y.; Wu, Z. Y.; Gupta, S.; Rivera, D. J.; Lambeets, S. V.; Pecaut, S.; Kim, J. Y. T.; Zhu, P.; Finfrock, Y. Z.; Meira, D. M.; King, G.; Gao, G.; Xu, W.; Cullen, D. A.; Zhou, H.; Han, Y.; Perea, D. E.; Muhich, C. L.; Wang, H., Efficient conversion of low-concentration nitrate sources into ammonia on a Ru-dispersed Cu nanowire electrocatalyst. *Nat. Nanotechnol.* ***2022,*** *17* (3), 759-767.

11. Andersen, S. Z.; Colic, V.; Yang, S.; Schwalbe, J. A.; Nielander, A. C.; McEnaney, J. M.; Enemark-Rasmussen, K.; Baker, J. G.; Singh, A. R.; Rohr, B. A.; Statt, M. J.; Blair, S. J.; Mezzavilla, S.; Kibsgaard, J.; Vesborg, P. C. K.; Cargnello, M.; Bent, S. F.; Jaramillo, T. F.; Stephens, I. E. L.; Norskov, J. K.; Chorkendorff, I., A rigorous electrochemical ammonia synthesis protocol with quantitative isotope measurements. *Nature* **2019,** *570* (7762), 504-508.

12. Nielander, A. C.; McEnaney, J. M.; Schwalbe, J. A.; Baker, J. G.; Blair, S. J.; Wang, L.; Pelton, J. G.; Andersen, S. Z.; Enemark-Rasmussen, K.; Čolić, V.; Yang, S.; Bent, S. F.; Cargnello, M.; Kibsgaard, J.; Vesborg, P. C. K.; Chorkendorff, I.; Jaramillo, T. F., A Versatile Method for Ammonia Detection in a Range of Relevant Electrolytes via Direct Nuclear Magnetic Resonance Techniques. *ACS Catal.* **2019,** *9* (7), 5797-5802.

13. Bard, A. J.; Faulkner, L. R.; Leddy, J.; Zoski, C. G., *Electrochemical methods: fundamentals and applications*. wiley New York: 1980; Vol. 2.

14. Wang, Y.; Wang, C.; Li, M.; Yu, Y.; Zhang, B., Nitrate electroreduction: mechanism insight, in situ characterization, performance evaluation, and challenges. *Chem. Soc. Rev.* **2021,** *50* (12), 6720-6733.

15. Shinagawa, T.; Garcia-Esparza, A. T.; Takanabe, K., Insight on Tafel slopes from a microkinetic analysis of aqueous electrocatalysis for energy conversion. *Sci. Rep.* **2015,** *5* (1), 13801.

16. Bai, L.; Lee, S.; Hu, X., Spectroscopic and electrokinetic evidence for a bifunctional mechanism of the oxygen evolution reaction. *Angew. Chem. Int. Ed.* **2021,** *60* (6), 3095-3103.
17. Bai, L.; Hsu, C.-S.; Alexander, D. T. L.; Chen, H. M.; Hu, X., Double-atom catalysts as a molecular platform for heterogeneous oxygen evolution electrocatalysis. *Nat. Energy* **2021,** *6* (11), 1054-1066.
18. Nicholson, R. S.; Shain, I., Theory of stationary electrode polarography. Single scan and cyclic methods applied to reversible, irreversible, and kinetic systems. *Anal. Chem.* **1964,** *36* (4), 706-723.
19. Levich, V. G., Physicochemical hydrodynamics. **1962**.
20. Wang, Y.; Shao, M., Theoretical Screening of Transition Metal–N_4-Doped Graphene for Electroreduction of Nitrate. *ACS Catal.* **2022,** *12* (9), 5407-5415.
21. Niu, H.; Zhang, Z.; Wang, X.; Wan, X.; Shao, C.; Guo, Y., Theoretical Insights into the Mechanism of Selective Nitrate-to-Ammonia Electroreduction on Single-Atom Catalysts. *Adv. Funct. Mater.* **2020,** *31* (11), 2008533.
22. Goyal, A.; Koper, M. T. M., The Interrelated Effect of Cations and Electrolyte pH on the Hydrogen Evolution Reaction on Gold Electrodes in Alkaline Media. *Angew. Chem. Int. Ed.* **2021,** *60* (24), 13452-13462.
23. Liu, H.; Lang, X.; Zhu, C.; Timoshenko, J.; Ruscher, M.; Bai, L.; Guijarro, N.; Yin, H.; Peng, Y.; Li, J.; Liu, Z.; Wang, W.; Cuenya, B. R.; Luo, J., Efficient Electrochemical Nitrate Reduction to Ammonia with Copper-Supported Rhodium Cluster and Single-Atom Catalysts. *Angew. Chem. Int. Ed.* **2022,** *61* (23), e202202556.
24. Neta, P.; Maruthamuthu, P.; Carton, P.; Fessenden, R., Formation and reactivity of the amino radical. *J. Phys. Chem.* **1978,** *82* (17), 1875-1878.
25. Grosse, P.; Yoon, A.; Rettenmaier, C.; Herzog, A.; Chee, S. W.; Roldan Cuenya, B., Dynamic transformation of cubic copper catalysts during CO_2 electroreduction and its impact on catalytic selectivity. *Nat. Commun.* **2021,** *12* (1), 6736.
26. Hu, T.; Wang, C.; Wang, M.; Li, C. M.; Guo, C., Theoretical Insights into Superior Nitrate Reduction to Ammonia Performance of Copper Catalysts. *ACS Catal.* **2021,** *11* (23), 14417-14427.
27. Wang, Y.; Qin, X.; Shao, M., First-principles mechanistic study on nitrate reduction reactions on copper surfaces: Effects of crystal facets and pH. *J. Catal.* **2021,** *400*, 62-70.
